# Benchmarking taxonomic assignments based on 16S rRNA gene profiling of the microbiota from commonly sampled environments

**DOI:** 10.1093/gigascience/giy054

**Published:** 2018-05-11

**Authors:** Alexandre Almeida, Alex L Mitchell, Aleksandra Tarkowska, Robert D Finn

**Affiliations:** 1 EMBL-EBI European Bioinformatics Institute, Wellcome Genome Campus, Hinxton, Cambridge CB10 1SD, UK; 2 Wellcome Trust Sanger Institute, Wellcome Genome Campus, Hinxton CB10 1SA, UK

**Keywords:** 16S rRNA gene, human gastrointestinal tract, ocean, microbiome, soil, taxonomy

## Abstract

**Background:**

Taxonomic profiling of ribosomal RNA (rRNA) sequences has been the accepted norm for inferring the composition of complex microbial ecosystems. Quantitative Insights Into Microbial Ecology (QIIME) and mothur have been the most widely used taxonomic analysis tools for this purpose, with MAPseq and QIIME 2 being two recently released alternatives. However, no independent and direct comparison between these four main tools has been performed. Here, we compared the default classifiers of MAPseq, mothur, QIIME, and QIIME 2 using synthetic simulated datasets comprised of some of the most abundant genera found in the human gut, ocean, and soil environments. We evaluate their accuracy when paired with both different reference databases and variable sub-regions of the 16S rRNA gene.

**Findings:**

We show that QIIME 2 provided the best recall and F-scores at genus and family levels, together with the lowest distance estimates between the observed and simulated samples. However, MAPseq showed the highest precision, with miscall rates consistently <2%. Notably, QIIME 2 was the most computationally expensive tool, with CPU time and memory usage almost 2 and 30 times higher than MAPseq, respectively. Using the SILVA database generally yielded a higher recall than using Greengenes, while assignment results of different 16S rRNA variable sub-regions varied up to 40% between samples analysed with the same pipeline.

**Conclusions:**

Our results support the use of either QIIME 2 or MAPseq for optimal 16S rRNA gene profiling, and we suggest that the choice between the two should be based on the level of recall, precision, and/or computational performance required.

## Findings

### Background

Genome sequencing has provided an unprecedented view of the microbial diversity of ecosystems from wide-ranging environments. For example, the commensal flora of the human gut has been extensively explored for potential associations with the onset of many human diseases [1-3]. Similarly, the rich microbial diversity of environments such as soil and oceans has been studied in depth, yielding important ecological inferences [4-6]. There are now a substantial number of such microbial community datasets deposited in sequence archives (e.g., the European Nucleotide Archive currently holds over 600,000 environmental samples [7]),and the rate of deposition is increasing. Drawing relevant biological correlations from this vast amount of data requires accurate and reliable tools and methods.

One of the crucial steps in almost all microbiome-based analyses is inference of community composition through taxonomic classification. For a few decades now [8], the common approach for taxonomic assignment of microbial species has been the classification of ribosomal RNA (rRNA) sequences. Currently, the most widely used tools for this purpose are the mothur [9] and Quantitative Insights Into Microbial Ecology (QIIME) software packages [10]. These correspond to large toolsets that are able to process, classify, and perform downstream analyses on individual genetic markers (e.g., the 16S rRNA gene, conserved across the prokaryotic domains). For taxonomic classification, each tool compares a set of queried sequences against a defined reference database, such as Greengenes [11], NCBI [12], RDP [13], or SILVA [14], assigning the most likely taxonomic lineages. Ultimately, the success of these analyses is not only dependent on the breadth and diversity of annotated sequences available in public repositories, but also on the accuracy of the classification algorithms used by each of the tools. By default, QIIME makes use of the UCLUST clustering method [15] to assign biological sequences to a reference database, while mothur reimplements the naïve Bayesian RDP classifier, developed by Wang et al. [16]. Two other tools, MAPseq [17] and QIIME 2 [18], have recently been released, the latter of which has officially replaced QIIME as of January 2018. QIIME 2 also makes use of a naïve Bayes classifier [19], and MAPseq is a *k-mer* search approach that outputs confidence estimates at different taxonomic ranks.

A community-driven initiative known as the “Critical Assessment of Metagenome Interpretation” benchmarked a range of software tools for the analysis of shotgun metagenomic datasets [20]. In regard to amplicon-based approaches, previous studies have mainly evaluated the classification methods of QIIME and mothur, highlighting some of their advantages and pitfalls [21-23]. The recent publication of QIIME 2 also included the assessment of a number of different commonly used classifiers and marker gene regions [19]. However, until now, no independent study has compared the accuracy of MAPseq, mothur, QIIME, and QIIME 2 whilst also taking into account potential differences arising from the use of distinct reference databases. Furthermore, for genotyping the 16S rRNA gene there is also much debate within the scientific community on the most informative variable sub-region to target [24]. Strong arguments have been made towards sequencing specific or combined sub-regions, such as the V4 [25] and V3-V4 [26], while difficulty in amplifying bacterial species, such as those from the *Actinobacteria* group, has prompted the development of more specialized primers [27, 28]. The impact of variable region choice on the taxonomic classification performance of different tools or databases is therefore also important to assess.

The use of mock communities in microbiome studies has revealed that different experimental conditions and methods dramatically affect the quality of the results [29-32]. In contrast, *in silico* benchmarking approaches provide an agnostic view on the efficiency of the computational pipelines—independently of experimental variation and technical biases—but may require further validation in real-world datasets. Hence, for a holistic assessment of the validity of different methodological strategies, using both mock communities and *in silico* simulations is essential to understand the biases and limitations present at each stage of analysis.

In this work we have leveraged a set of simulated 16S rRNA gene sequences representative of genera commonly found in the human gut, ocean, and soil environments to evaluate the accuracy of the default taxonomic classifiers of MAPseq, mothur, QIIME, and QIIME 2. We tested these methods with different reference databases and according to some of the most commonly targeted sub-regions of the 16S rRNA gene. Our results showed that, regardless of the database used, QIIME 2 outperformed all other tools in terms of overall recall at both genus and family levels as well as in distance estimations between the observed and predicted samples. Considerable performance differences were observed between using distinct 16S rRNA gene sub-regions, while limited software-dependent variation was seen between different reference databases. We believe this work will help inform microbial ecologists about important decisions to take when designing new 16S rRNA-based community studies.

### Composition of the simulated datasets

The microbiota colonizing the human gut, ocean, and soil environments are some of the most frequently studied microbial communities. Hence, to provide data with direct practical applications, we focused on simulating datasets containing a diverse set of genera commonly found in these three ecosystems (Additional file: Fig. S1). Representative genera were selected after identifying the 80 most abundant genera across publicly available metagenomes from human gut, ocean, and soil [7]. Then, for each biome, four different communities were generated with two levels of diversity: samples A100 and B100 with a random set of 100 species belonging to these genera, and A500 and B500 with 500 species. Final datasets comprised a total of 66, 66, and 76 different genera from the human gut, oceanic, and soil environments, respectively. For the purpose of this benchmarking, we simulated the datasets with a similar relative abundance per genus to avoid introducing any taxon-specific biases (Additional file: Fig. S1).

To simulate a realistic scenario, where variation can arbitrarily occur and sequences may not have an exact representative in public databases, we randomly mutated 2% of the positions of each 16S rRNA sequence retrieved after extracting each sub-region using commonly used primer sequences [26, 27, 33-35] (Additional file: Table S1). Notably, the percentage of sequences retrieved from the Greengenes, NCBI, RDP, and SILVA databases matching the primers selected for V1-V2 was dramatically lower (30.3%) than that of V3-V4 (90%), V4 (90.9%),and V4-V5 (87.8%) (Additional file: Fig. S2). The 16S rRNA V1 sub-region had been previously found to be truncated in a substantial number of reference sequences [24]. Our results confirm this observation and again raise caution at the use of the 16S V1-V2 rRNA primer sequences for complex and diverse samples due to the reduced number of reference sequences available. Interestingly, the relative number of sequences retrieved from RDP was lower than that of the remaining databases (Additional file: Fig. S2), likely suggesting an overrepresentation of more divergent taxa that did not meet the mismatch threshold used in our *in silico* PCR.

### Taxonomic assignment

Microbiome studies frequently strive to associate microbial diversity signatures with a phenotype of interest. However, focusing solely on high-level taxonomic ranks can severely underestimate the degree of variation observed between sample groups. To circumvent this, highly discriminative approaches are needed to be able to pinpoint the most significant taxa warranting further validation. For assessing the performance of MAPseq, mothur, QIIME, and QIIME 2 with different reference databases (Additional file: Fig. S3), we limited our analyses to classification at the lineage level instead of operational taxonomic units, as it allows a more consistent and easier interpretation of the results. Species assignment of every queried sequence would be the desired outcome, but as was previously shown [21], the limited resolution of the 16S rRNA locus precludes an accurate classification at this level. Furthermore, there is significant inconsistency in species nomenclature across all reference databases (e.g., RDP does not report taxon names below genus). In this work, we calculated the degree of recall and precision at the genus and family ranks, as in our opinion they provide the best compromise between classification accuracy and resolution.

**Figure 1:**
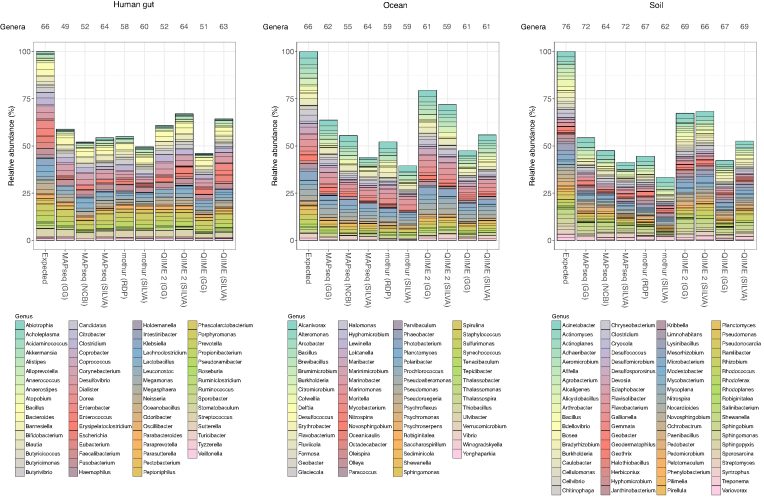
Level of recall at the genus level, represented as taxa relative abundances, obtained with each analysis pipeline for the three different biomes (human gut, ocean, and soil). The number of genera correctly identified by each pipeline is indicated above the graph.

By comparing the level of recall across all software tools, we found that QIIME 2 recovered the largest proportion of sequences from the expected genera (Table 1, Fig. 1, and Additional files: Tables S2–S4). Combined with the SILVA database, this resulted in the highest recall (sensitivity) for human gut (67.0%) and soil samples (68.3%), while the Greengenes database outperformed in the case of the oceanic microbiome (79.5%). In fact, all tools except QIIME saw a decrease in recall when using SILVA specifically for the classification of the oceanic dataset. Globally, however, SILVA most frequently provided a better genus recall than Greengenes (five out of nine comparisons across MAPseq, QIIME, and QIIME 2; Fig. 1). In terms of correctly identified taxa, MAPseq in conjunction with SILVA detected the greatest number of expected genera in all three biomes (Fig. 1). At the family level, all tools presented a substantially higher recall (Table 1), with QIIME 2 reaching 94.3% in the human gut sample, 96.2% with the ocean set, and 91.7% with the soil sample (Additional files: Tables S2–S4).

**Table 1:** Global metrics averaged across the analyses of simulated samples from human gut, ocean, and soil

		Family		Genus					
Software	Database	Recall	Miscalled	Recall	Miscalled	Sub-region^a^	Mean DS	Bray-Curtis	Jaccard
MAPseq	Greengenes	88.3	2.4	58.9	2.4	V3-V4	0.434	0.282	0.440
MAPseq	NCBI	81.7	1.3	51.7	2.0	V3-V4	0.522	0.330	0.495
MAPseq	SILVA	67.2	**0.7**	46.5	**1.0**	V3-V4	0.482	0.373	0.540
mothur	RDP	85.4	3.2	50.5	5.0	V3-V4	0.419	0.356	0.523
mothur	SILVA	82.9	2.4	40.8	5.2	V3-V4	0.492	0.446	0.613
QIIME 2	Greengenes	93.2	1.6	**69.2**	3.4	V3-V4	0.367	**0.210**	**0.342**
QIIME 2	SILVA	**93.6**	1.9	69.0	4.3	V3-V4	**0.331**	0.211	0.348
QIIME	Greengenes	59.4	1.6	45.1	2.5	V4	0.585	0.394	0.564
QIIME	SILVA	66.4	2.1	57.5	6.5	V4	0.432	0.309	0.470

Values in bold denote the best score.

^a^Sub-region with the highest F-score, excluding V1-V2.

**Figure 2:**
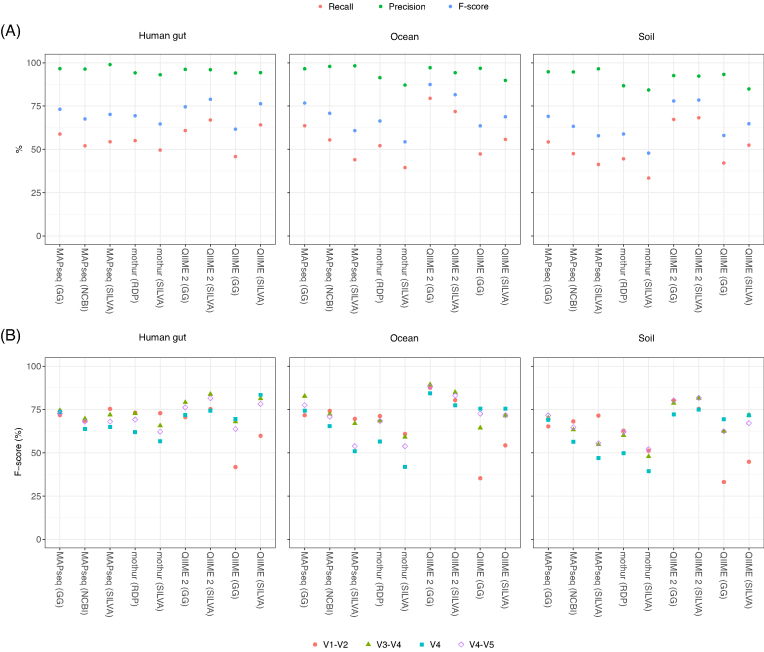
A) Recall, precision, and F-score estimates at the genus level for each tool and database tested. B) F-scores calculated for some of the most commonly tested sub-regions of the 16S rRNA gene: V1-V2, V3-V4, V4, and V4-V5.

Although the level of recall is a crucial metric in choosing the most appropriate taxonomic classification pipeline, it is equally important to ensure a low frequency of false-positive assignments. We evaluated the degree of precision (specificity) by the percentage of sequences assigned to the wrong taxon (Additional files: Tables S5–S7) out of all the detected taxa. Accuracy was high for all the tools, with precision estimates of at least 84% across all analysis pipelines (Fig. 2A). In terms of total number of sequences, this translated to <10% of the reads misassigned at the genus level (Additional files: Tables S2–S4 and Fig. S4). MAPseq with the SILVA database consistently outperformed all other tools, with a precision >96% for the three tested biomes (Fig. 2A), equating to <2% of miscalled sequences.

To combine both recall and precision into a single metric, we calculated the F-score for all taxonomic assignments ( Figs 2A and S5). At both genus and family levels, we found that QIIME 2 had the highest score across the samples representative of the three different biomes, with the SILVA database coming out on top for the human gut (genus: 78.9%, family: 96.8%; Additional file: Table S2) and soil (genus: 78.5%, family: 94.3%; Additional file: Table S4) environments in particular, but the Greengenes database performing better with the oceanic dataset (genus: 87.4%, family: 97.4%; Additional file: Table S3). After fractioning the data according to different sub-regions of the 16S rRNA gene, we then repeated the same analysis (Fig. 2B). This revealed that the performance of each tool varied up to 40% depending on the 16S rRNA sub-region targeted. Notably, the V1-V2 or V3-V4 sub-regions performed the best across most of the pipelines (Fig. 2B). In our study, each synthetic species had a genetically close full-length 16S rRNA sequence represented in the databases, so our tests were probably not significantly affected by the reduced number of V1-V2 reference sequences available.

**Figure 3:**
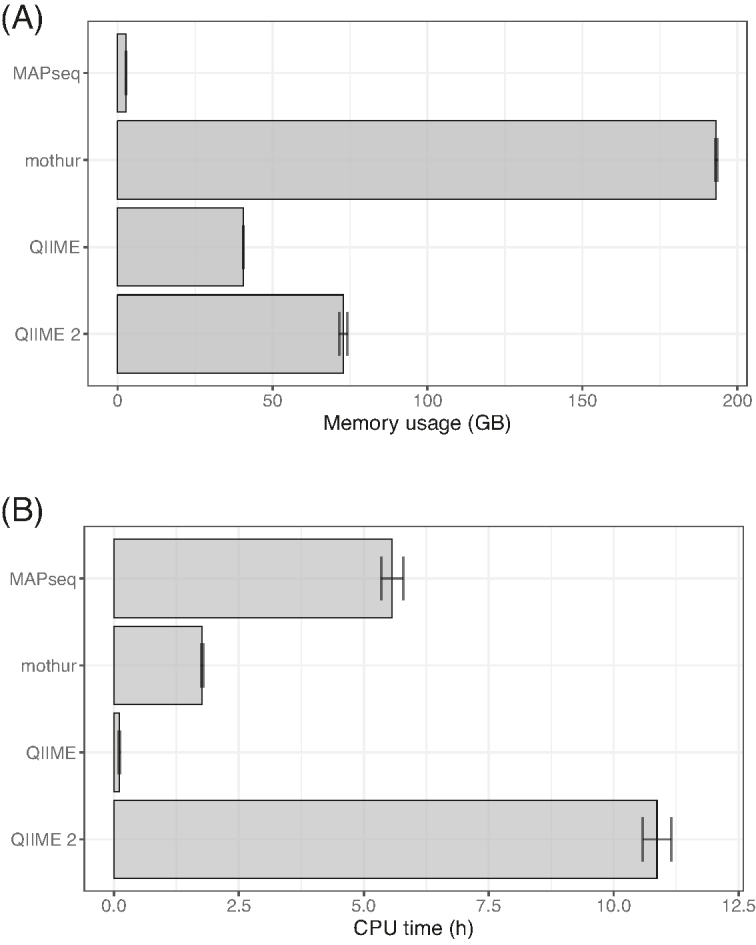
Computational cost of each taxonomy assignment tool, estimated as the total memory usage (A) and CPU time (B) required for the processing and classification of ∼3 million sequences against the SILVA 128 database. Error bars denote standard deviation across the three biomes tested (human gut, ocean, and soil).

The ongoing surge in genome sequencing is producing thousands of novel sequences each year. Therefore, efficient tools that can scale up to provide analysis of tens of thousands of samples is increasingly important. With this in mind, we compared the computational performance of MAPseq, mothur, QIIME, and QIIME 2 throughout the whole classification pipeline of our simulated datasets. We analysed average memory usage and CPU time across the three biomes for the processing and assignment of 3 million quality-filtered sequences against the SILVA 128 database (Fig. 3). MAPseq was the most memory-efficient tool, with mothur, QIIME, and QIIME 2 requiring more than 72, 15, and 27 times more memory resources, respectively (Fig. 3A). CPU time of QIIME 2 was the highest, close to twice that of MAPseq, and almost 100 times longer than QIIME, which was the fastest (Fig. 3B). Of note is that each pipeline has its own processing procedure; both the mothur and QIIME 2 pipelines included a de-replication step of the query sequences prior to taxonomic assignment, which substantially reduced the number of sequences used for classification.

### Relative quantification and beta diversity

**Figure 4:**
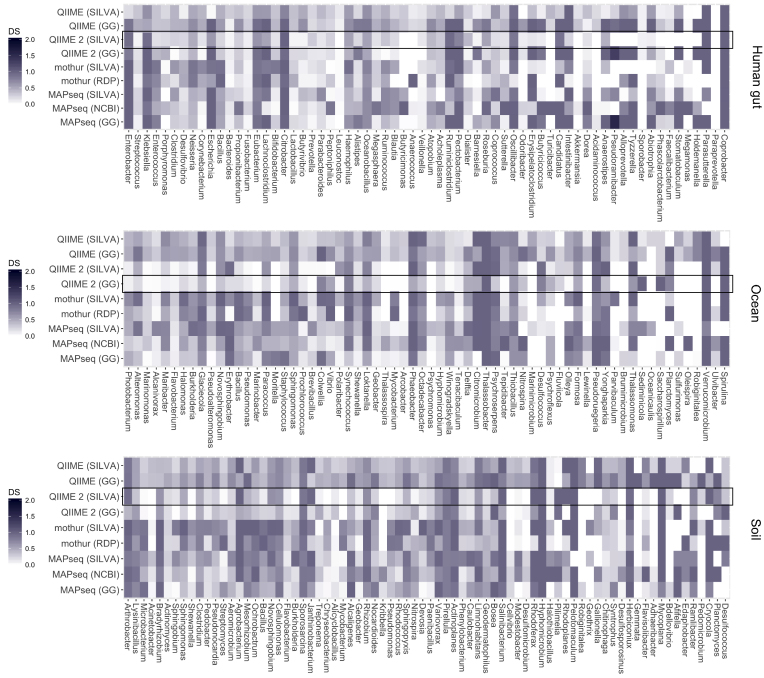
DS calculated for each genus included in the simulated datasets. Lower (brighter) values indicate a closer prediction to the true composition of the original sample. The black outline indicates the overall best scoring analysis pipeline for each environment. Taxa are ordered by decreasing abundance from left to right based on their composition in the simulated sample.

One of the main aspects of any microbiome-based analysis is the assessment of the differential abundance and beta diversity across a set of sample groups. In this respect, accurate estimation of the relative abundance of each taxon is essential to find statistically significant patterns. To assess how accurately each tool was able to predict taxa relative abundances in each sample, we calculated dissimilarity scores for each genus present in the simulated dataset (Fig. 4). Interestingly, QIIME 2 showed the most accurate prediction in relation to the true genera composition, with an average dissimilarity score of 0.33 when used in conjunction with the SILVA database (Table1). In terms of the reference database used, analyses carried out with SILVA yielded more accurate predictions than with the Greengenes database (Additional files: Tables S2–S4). Substantial differences in accuracy were observed across different genera, with sequences from the *Paraprevotella* genus—frequently present in human gut samples—more accurately predicted, in contrast to those from *Coprobacter, Hyphomicrobium*, and *Thalassobacter*, which had the worst results (Fig. 4). These genera might either be underrepresented in the reference databases or have a high degree of conservation with other closely related taxa, making accurate taxonomic assignments more challenging.

**Figure 5:**
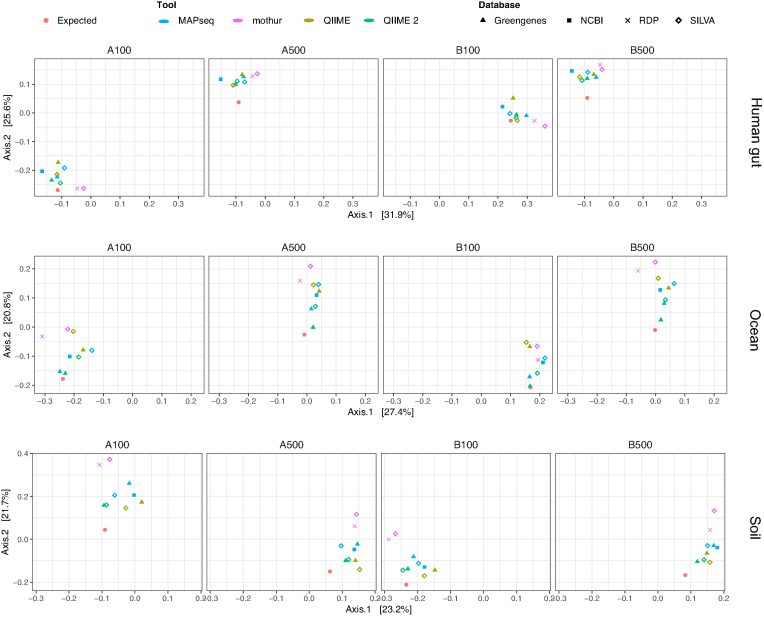
PCoA between all samples analysed in relation to the true, expected dataset, using the Bray-Curtis distance method.

For a global assessment of the beta diversity across samples, we performed a principal coordinates analysis (PCoA) and calculated both Bray-Curtis and Jaccard distances between the observed and expected results. Both distance methods represent complementary approaches, as the Bray-Curtis metric corresponds to a quantitative evaluation of the dissimilarity across samples, whereas the Jaccard index is a qualitative measure of community similarity. We found that samples analysed with QIIME 2 were the closest (i.e., had the lowest distance estimate) to the true simulated datasets, with minor differences between the use of SILVA or Greengenes with both the Bray-Curtis and Jaccard methods (Table 1; Fig. 5).

## Discussion

With the number of tools, databases, and options available for taxonomic classification of marker sequences, it can be a daunting task to decide the optimal approach for analysis of a specific dataset. In this work, we have strived to help guide this decision-making process by independently assessing the performance of the most commonly used taxonomic assignment strategies with simulated samples comprised of genera found in frequently sampled environments.

Overall, we show that all tools we tested performed moderately well, with high precision and modest-to-high recall rates at the genus level. QIIME 2 presents significant improvements over the other tools, particularly over the preceding version of QIIME, in regard to detection sensitivity at both family and genus levels. It should be emphasized that as of January 2018, QIIME has been replaced by QIIME 2 and the former tool is no longer supported by the developers. The superiority of QIIME 2 also held true for the prediction of sample composition, as beta diversity estimates between the analysed and simulated communities were the closest using this method. Therefore, these data support the use of QIIME 2 to obtain the largest proportion of classified sequences at the most accurate relative abundances. Nevertheless, the results also showed MAPseq to be a more conservative and precise approach, meaning that fewer genera were misassigned. In addition, this tool showed considerably better computational performance than QIIME 2, requiring approximately 30 times less memory and almost one-half the CPU time to process the same dataset (even though QIIME 2 classifies substantially fewer query sequences due to a prior de-replication step). These results show that MAPseq provides a credible option if precision and computational performance or scale are a priority.

Selecting a single best software package is not a straightforward affair, and we expect that further differences in performance will be observed with different real-world datasets. Additionally, mothur and QIIME 2 also provide the option of using multiple taxonomic classifiers, so improvements in overall recall and/or precision metrics might be possible with the other available methods, combined with further parameter optimization. We should also stress that, aside from the software packages we tested, other web-based tools such as BioMaS [23] are also available. However, they are usually restricted to the use of specific reference databases, making individual customizations and accurate comparisons more challenging.

In addition to choosing the right tool, combining that with the appropriate reference database is equally important to ensure the best classification performance. Greengenes and SILVA have been the most widely used and readily supported databases. Generally, the SILVA 128 database performed better than Greengenes 13_8 in terms of recall at both genus and family levels as well as in predicting the true taxa composition of the simulated communities. Conversely, there was an almost universal decrease in its performance in the detection of ocean-specific taxa, so special care should be taken in the analysis of datasets sampled from this particular environment. Nonetheless, there are additional advantages to the use of SILVA: it is more frequently updated (Greengenes was last updated in May 2013); it includes rRNA sequences of eukaryotic organisms in addition to archaea and bacterial species; and has been shown to be more easily comparable and mapped to other taxonomies such as the NCBI [36]. In the case of MAPseq and mothur, the NCBI and RDP databases also performed well, with higher recall but slightly lower precision scores compared to SILVA. Therefore, the SILVA, RDP, or NCBI databases are all appropriate choices for a comprehensive and accurate taxonomic analysis.

The choice of primer sequences for taxonomic profiling of the 16S rRNA gene has been a matter of frequent debate. In common with previously reported observations [28], we show that targeting different sub-regions can considerably influence the taxonomic assignment performance (by up to 40% in our analyses). Overall, the V1-V2 and V3-V4 sub-regions performed the best across most of the tools. However, the V1-V2 primers did not match almost 70% of the sequences across the four reference databases, so we discourage its use for classification of complex community samples. As our simulated datasets were generated from close representatives containing full-length 16S rRNA genes, it is reasonable to assume that our analysis of the V1-V2 sub-region was not significantly hampered by this reduced number of reference sequences. Kozich et al. [25] have argued in favour of standardizing the use of the V4 sub-region for Illumina MiSeq sequencing, because it allows complete overlap of paired-end sequences, mitigating sequence errors introduced during PCR amplification or sequencing. Phylogenetic studies have also showed that the V4 sub-region is the closest representative of the phylogenetic signal of the whole 16S rRNA locus [24]. Here, we analysed the performance of some of the most commonly used sub-regions under a purely computational perspective and conclude that amplification of the V3-V4 sub-region is most frequently the best option for a reliable taxonomic inference.

In summary, we have identified the major benefits and drawbacks of the most recent and popular taxonomic classification methods. Importantly, we show that the choice of software, database, and sub-region significantly affects the quality of the classification results. Given the impact of each of these variables, it is imperative to strive for consistency in the analysis of samples not only within individual studies, but across different projects as well. Services like the EBI Metagenomics [7] and MG-RAST [37] help provide a basis for standardization, but additional factors relating to the experimental design are up to individual users to decide. Some attempts have been made to find recommended best practices for 16S microbiome studies among the myriad of options and issues that can arise at each analysis stage [38]. We believe our work presented here further complements these efforts by helping the microbiome research community make more informed decisions about the most appropriate methodological approach to take in their own analysis pipeline.

## Methods

### Generating simulated datasets

Twelve sets of synthetic communities were generated for evaluating the accuracy of the taxonomic assignment pipelines: four each for human gut, ocean, and soil environments. First, the 80 most abundant genera across publicly deposited samples from these biomes were retrieved using the EBI Metagenomics API [7, 39]. This list was then used to randomly select either 100 (datasets A100 and B100) or 500 species (datasets A500 and B500) belonging to these genera, allowing a maximum of 20 and 50 species per genus, respectively. 16S rRNA gene sequences were obtained from the European Nucleotide Archive, and an *in silico* PCR was carried out with a python script [40] to extract commonly used regions for 16S rRNA profiling [26, 27, 33-35] (Additional file: Table S1), allowing a maximum of three mismatches per primer sequence. Subsequently, 2% of the positions in each variable region were randomly mutated to create nucleotide diversity using a custom python script [41]. Sequencing reads were simulated from these amplicon sequences in duplicate using the MiSeq v3 error profile with ART (ART, RRID:SCR_006538) [42], generating ∼ 10,000 and ∼ 200,000 paired-end reads of 250 bp per region to have samples representing both low and high levels of sequencing depth.

### Sequence classification

Initial pre-processing and quality control was performed following the mothur standard operating procedure (SOP) [25] accessed on November 2017. Briefly, the *make.contigs* command was used to align, filter, and merge the paired-end reads into contigs. Subsequently, we used the *screen.seqs* command to filter out any sequences with ambiguous base calls. This final set of quality-controlled sequences was then assigned into taxonomic lineages with MAPseq v1.2.2 [17], mothur v1.39.5 (mothur, RRID:SCR_011947) [9], QIIME 1.9.1 (QIIME, RRID:SCR_008249) [10], and QIIME 2 v2017.11 [18]. For each software, we evaluated the settings and databases most frequently used and recommended for optimal taxonomic classification (Additional file: Fig. S3). With MAPseq, we tested the default NCBI database (mapref 2.2) as well as Greengenes 13_8 and the SILVA 128 database re-mapped to an eight-level taxonomy (available in [44]). Each set of reference sequences was analysed following the internal clustering by MAPseq. Options *-tophits 80* and *-topotus 40* were used in combination with the *-outfmt simple* option. For QIIME 1.9.1, the *pick_closed_reference_otus.py* script was used with the default Greengenes database (13_8) and with SILVA 128, both clustered at 97% identity. Taxonomic assignment with mothur was carried out according to the MiSeq SOP [25], excluding the chimera detection and removal steps, using the available pre-formatted SILVA 128 database for alignment and either the RDP version 16 or SILVA 128 for sequence classification. We did not use the Greengenes alignment database as per the mothur SOP [45]. Lastly, for QIIME 2 we first dereplicated the query sequences using the *vsearch dereplicate-sequences* function and then assigned them to the Greengenes (13_8) or SILVA 128 (99% identity clusters) databases using the *feature-classifier classify-sklearn* function [19].

### Analysis and visualization

TSV and BIOM files were generated from the MAPseq and QIIME 2 outputs and combined with the output BIOM files created by QIIME and mothur (*make.biom* command). Taxonomy names obtained from each individual reference database were normalized so that each genus and family would be assigned to the same lineage. Results were visualized and analysed with the phyloseq (phyloseq, RRID:SCR_013080) [46] and vegan R packages (vegan, RRID:SCR_011950). The recall rate (sensitivity) for each tool and database was estimated as the percentage of sequences assigned to the expected taxa for each biome, while precision (specificity) was calculated as the fraction of sequences from these predicted taxa out of all those from the taxa observed. Finally, the F-score was calculated as follows:

\begin{equation*}
{\rm{F\text{-}score}} = 2 \times \frac{{{\rm{precision}} \times {\rm{recall}}}}{{{\rm{precision}} + {\rm{recall}}}}
\end{equation*}

Distance estimates were calculated with either the Bray-Curtis or Jaccard dissimilarity indices after grouping the taxonomic lineages at the genus level. PCoA were performed with the Bray-Curtis distance method. Dissimilarity scores on the relative abundance (rel.ab) of each expected genus were calculated as:

\begin{equation*}
{\rm{DS}} = \frac{{\left| {{\rm{rel}}.{\rm{ab}}.\left( {{\rm{Observed}}} \right) - {\rm{rel}}.{\rm{ab}}.\left( {{\rm{Expected}}} \right)} \right|}}{{{\rm{rel}}.{\rm{ab}}.\left( {{\rm{Expected}}} \right)}}
\end{equation*}

Memory usage and CPU time were estimated as the total amount required for the processing and assignment of all combined sequences against the SILVA 128 database (clustered at 99%), following the protocols described above.

## Availability of supporting source code and requirements

Project name: Taxonomy benchmarking

Project home page: https://github.com/Finn-Lab/Tax-Benchmarking

Operating system: Platform independent

Programming languages: Python 2.7, R 3.4.1

Other requirements: BioPython module, R libraries (ggplot2, phyloseq, vegan, scales, grid, ape, RColorBrewer, data.table)

License: MIT

## Availability of supporting data

The datasets supporting the conclusions of this article are available in the GigaDB repository [47].

## Additional files

Figure S1. Composition of the synthetic communities per selected environment. Samples A100 and B100 were randomly generated sets of 100 species, while A500 and B500 were simulated from 500 different species.

Figure S2. Percentage of sequences retrieved from the Greengenes, NCBI, RDP, and SILVA databases with an *in silico* PCR targeting different 16S rRNA gene sub-regions.

Figure S3. Tools and databases benchmarked in our study. We tested at least two databases per software tool. The reference databases used were either readily supported by the specific tool and/or recommended by their developers. SILVA was compared across all tools; MAPseq was specifically assessed with the NCBI database, its default reference; mothur was not paired with Greengenes due to its poor-quality alignment [45] and was analysed with RDP instead.

Figure S4. Number of genera misassigned in each analysis pipeline and their overall relative abundance. Names and abundance values of each misclassified taxon are included as additional files (Additional files: Tables S5–S7).

Figure S5. Recall, precision, and F-score estimates at the family level for each tool and database tested.

Table S1. Primer pairs used in the in silico PCR.

Table S2. Global metrics across the analyses of simulated samples from human gut.

Table S3. Global metrics across the analyses of simulated samples from ocean environments.

Table S4. Global metrics across the analyses of simulated samples from soil.

Table S5. Relative abundances (%) of the genera miscalled using simulated human gut samples.

Table S6. Relative abundances (%) of the genera miscalled using simulated oceanic samples.

Table S7. Relative abundances (%) of the genera miscalled using simulated soil samples.

## Abbreviations

CPU: Central Processing Unit; DS: dissimilarity score; GG: Greengenes; OTU: Operational Taxonomic Unit; PCoA: principal coordinates analysis; RDP: Ribosomal Database Project; rRNA: ribosomal rRNA; SOP: standard operating procedure.

## Ethics approval and consent to participate

Not applicable

## Consent for publication

Not applicable

## Competing interests

The authors declare that they have no competing interests.

## Funding

European Molecular Biology Laboratory; European Commission within the Research Infrastructures Programme of Horizon 2020 [676559] (ELIXIR-EXCELERATE)

## Authors' contributions

A.A., A.L.M., A.T., and R.D.F. performed the analyses; A.A., A.L.M., and R.D.F. conceived the study and wrote the manuscript. All authors have read and approved the final manuscript.

## Supplementary Material

GIGA-D-18-00043.pdf

GIGA-D-18-00043_R1.pdf

Response_to_Reviewer_Comments_Original_Submission.pdf

Reviewer_1_Report_(Original_Submission) -- Nicholas Bokulich 2/20/2018 Reviewed

Reviewer_2_Report_(Original_Submission) -- Finlay Maguire 3/19/2018 Reviewed

Supplemental material
