# Supplementary material for: Benchmarking taxonomic assignments based on 16S rRNA gene profiling of the microbiota from commonly sampled environments
Source: Gigascience. 2018 May 11;7(5):giy054. doi: 10.1093/gigascience/giy054 (PMC5967554; doi:10.1093/gigascience/giy054)
Supplement: GIGA-D-18-00043.pdf [file giy054_giga-d-18-00043.pdf]

GigaScience

Benchmarking taxonomic assignments based on 16S rRNA gene profiling of the microbiota from commonly sampled environments

--Manuscript Draft--

Manuscript Number:	GIGA-D-18-00043
Full Title:	Benchmarking taxonomic assignments based on 16S rRNA gene profiling of the microbiota from commonly sampled environments
Article Type:	Technical Note
Funding Information:	European Bioinformatics Institute — Dr Alexandre Almeida<br>H2020 Research Infrastructures (676559) — Not applicable
Abstract:	Background: Taxonomic profiling of ribosomal RNA (rRNA) sequences has been the accepted norm for inferring the composition of complex microbial ecosystems. QIIME and mothur have been the most widely used taxonomic analysis tools for this purpose, with MAPseq and QIIME 2 being two recently released alternatives. However, no independent and direct comparison between these four main tools has been performed. Here, we compared MAPseq, mothur, QIIME, and QIIME 2 using synthetic simulated datasets comprised of some of the most abundant genera found in the human gut, ocean and soil environments. We evaluate their accuracy when paired with both different reference databases and variable sub-regions of the 16S rRNA gene.<br><br>Findings: We show that QIIME 2 with the SILVA 128 database provided the best recall at the genus level, and the lowest distance estimates between the observed and simulated samples. However, MAPseq showed the highest precision, with miscall rates consistently below 2%. Notably, QIIME 2 was the most computationally expensive tool, with CPU time and memory usage almost two and 30 times higher than MAPseq, respectively. Using the SILVA database generally yielded a higher recall than using Greengenes, while assignment results of different 16S rRNA variable sub-regions varied by up to 35% between samples analysed with the same pipeline.<br><br>Conclusions: Our results support the use of either QIIME 2 or MAPseq for optimal 16S rRNA gene profiling, and we suggest that the choice between the two should be based on the level of recall, precision and/or computational performance required.
Corresponding Author:	Alexandre Almeida<br><br>UNITED KINGDOM
Corresponding Author Secondary Information:	
Corresponding Author's Institution:	
Corresponding Author's Secondary Institution:	
First Author:	Alexandre Almeida
First Author Secondary Information:	
Order of Authors:	Alexandre Almeida<br>Alex L Mitchell<br>Aleksandra Tarkowska<br>Robert D Finn
Order of Authors Secondary Information:	
Opposed Reviewers:	
Additional Information:	

Powered by Editorial Manager® and ProduXion Manager® from Aries Systems Corporation

Question	Response
Are you submitting this manuscript to a special series or article collection?	No
**Experimental design and statistics**<br><br>Full details of the experimental design and statistical methods used should be given in the Methods section, as detailed in our Minimum Standards Reporting Checklist. Information essential to interpreting the data presented should be made available in the figure legends.<br><br>Have you included all the information requested in your manuscript?	Yes
**Resources**<br><br>A description of all resources used, including antibodies, cell lines, animals and software tools, with enough information to allow them to be uniquely identified, should be included in the Methods section. Authors are strongly encouraged to cite Research Resource Identifiers (RRIDs) for antibodies, model organisms and tools, where possible.<br><br>Have you included the information requested as detailed in our Minimum Standards Reporting Checklist?	Yes
**Availability of data and materials**<br><br>All datasets and code on which the conclusions of the paper rely must be either included in your submission or deposited in publicly available repositories (where available and ethically appropriate), referencing such data using a unique identifier in the references and in the "Availability of Data and Materials" section of your manuscript.<br><br>Have you have met the above requirement as detailed in our Minimum Standards Reporting Checklist?	Yes

Powered by Editorial Manager® and ProduXion Manager® from Aries Systems Corporation

Manuscript Click here to download Manuscript Manuscript.docx

Benchmarking taxonomic assignments based on 16S rRNA gene profiling of the microbiota from commonly sampled environments

Alexandre Almeida[1,2,*], Alex L. Mitchell[1], Aleksandra Tarkowska[1] and Robert D. Finn[1]

[1]EMBL-EBI European Bioinformatics Institute, Wellcome Genome Campus, Hinxton, Cambridge CB10 1SD, UK; [2]Wellcome Trust Sanger Institute, Wellcome Genome Campus, Hinxton CB10 1SA, UK

[*]Corresponding author:
Alexandre Almeida,
EMBL-EBI European Bioinformatics Institute,
Wellcome Trust Genome Campus, Hinxton, Cambridge CB10 1SD, UK
Tel + 44 (0) 1223 494 468 E-mail: aalmeida@ebi.ac.uk

Keywords: 16S rRNA gene, human gastrointestinal tract, ocean, microbiome, soil, taxonomy

Abstract

Background: Taxonomic profiling of ribosomal RNA (rRNA) sequences has been the accepted norm for inferring the composition of complex microbial ecosystems. QIIME and mothur have been the most widely used taxonomic analysis tools for this purpose, with MAPseq and QIIME 2 being two recently released alternatives. However, no independent and direct comparison between these four main tools has been performed. Here, we compared MAPseq, mothur, QIIME, and QIIME 2 using synthetic simulated datasets comprised of some of the most abundant genera found in the human gut, ocean and soil environments. We evaluate their accuracy when paired with both different reference databases and variable sub-regions of the 16S rRNA gene.

Findings: We show that QIIME 2 with the SILVA 128 database provided the best recall at the genus level, and the lowest distance estimates between the observed and simulated samples. However, MAPseq showed the highest precision, with miscall rates consistently below 2%. Notably, QIIME 2 was the most computationally expensive tool, with CPU time and memory usage almost two and 30 times higher than MAPseq, respectively. Using the SILVA database generally yielded a higher recall than using Greengenes, while assignment results of different 16S rRNA variable sub-regions varied by up to 35% between samples analysed with the same pipeline.

Conclusions: Our results support the use of either QIIME 2 or MAPseq for optimal 16S rRNA gene profiling, and we suggest that the choice between the two should be based on the level of recall, precision and/or computational performance required.

Findings

Background

Genome sequencing has provided an unprecedented view of the microbial diversity of ecosystems from wide-ranging environments. For example, the commensal flora of the human gut has been extensively explored for potential associations with the onset of many human diseases [1–3]. Similarly, the rich microbial diversity of environments such as soil and oceans have been studied in depth, yielding important ecological inferences [4–6]. There are now a substantial number of such microbial community datasets deposited in sequence archives (for example, the European Nucleotide Archive currently holds over 600 000 environmental samples [7]) and the rate of deposition is increasing. Drawing relevant biological correlations from this vast amount of data requires accurate and reliable tools and methods.

One of the crucial steps in almost all microbiome-based analyses is inference of community composition through taxonomic classification. For a few decades now [8], the common approach for taxonomic assignment of microbial species has been the classification of ribosomal RNA (rRNA) sequences. Currently, the most widely used tools for this purpose are the mothur [9] and “Quantitative Insights Into Microbial Ecology” (QIIME) software packages [10]. Both tools take individual genetic markers (e.g. the 16S rRNA gene, conserved across the prokaryotic domains) and compare them to a reference database, assigning a taxonomic lineage to each of the queried sequences. Greengenes [11], NCBI [12], RDP [13] and SILVA [14] are some of the most widely used rRNA sequence databases. Ultimately, the success of these analyses is not only dependent on the breadth and diversity of annotated sequences available in public repositories, but also on the accuracy of the classification algorithms used by each of the tools. By default, QIIME makes use of the

UCLUST clustering method [15] to assign biological sequences to a reference database, while mothur wraps the naïve Bayesian RDP classifier, developed by Wang, *et al*. [16], for sequence classification. Two other tools — MAPseq [17] and QIIME 2 (https://qiime2.org/) — have recently been released, providing additional assignment methods. QIIME 2 also makes use of a naïve Bayes classifier [18], and MAPseq is a *k-mer* search approach that outputs confidence estimates at different taxonomic ranks.

A community-driven initiative known as the “Critical Assessment of Metagenome Interpretation” (CAMI) benchmarked a range of software tools for the analysis of shotgun metagenomic datasets [19]. In regard to amplicon-based approaches, previous studies have mainly evaluated the classification methods of QIIME and mothur, highlighting some of their advantages and pitfalls [20–22]. However, until now, no independent study has compared the accuracy of these methods to MAPseq and QIIME 2 whilst also taking into account potential differences arising from the use of distinct reference databases. Furthermore, for genotyping the 16S rRNA gene there is also much debate within the scientific community on the most informative variable sub-region to target [23]. Strong arguments have been made towards sequencing specific or combined sub-regions, such as the V4 [24] and V3-V4 [25], while difficulty in amplifying bacterial species, such as those from the *Actinobacteria* group, has prompted the development of more specialized primers [26,27]. The impact of variable region choice on the taxonomic classification performance of different tools or databases is therefore also important to assess.

The use of mock communities in microbiome studies has revealed that different experimental conditions and methods dramatically affect the quality of the results [28–31]. In contrast, *in*

silico benchmarking approaches provide an agnostic view on the efficiency of the computational pipelines, independently of experimental variation and technical biases.

Therefore, in this work we have leveraged a set of simulated 16S rRNA gene sequences representative of genera commonly found in the human gut, ocean and soil environments, to evaluate the accuracy of MAPseq, mothur, QIIME and QIIME 2 with different reference databases, and according to some of the most commonly targeted sub-regions of the 16S rRNA gene. We show that, regardless of the database used, QIIME 2 outperformed all other tools in terms of overall recall at both genus and family levels, as well as in distance estimations between the observed and predicted samples. Considerable performance differences were observed between using distinct 16S rRNA gene sub-regions, while limited software-dependent variation was seen between different reference databases. We believe this work will help inform microbial ecologists about important decisions to take when designing new 16S rRNA-based community studies.

Composition of the simulated datasets

The microbiota colonizing the human gut, ocean and soil environments are some of the most frequently studied microbial communities. Hence, to provide data with direct practical applications, we focused on simulating datasets containing a diverse set of genera commonly found in these three ecosystems (Additional file: Fig. S1). Representative genera were selected after identifying the 80 most abundant genera across publicly available metagenomes from human gut, ocean and soil [7]. Then, for each biome, four different communities were generated with two levels of diversity: samples A100 and B100 with a random set of 100 species belonging to these genera; and A500 and B500 with 500 species. Final datasets comprised a total of 66, 66 and 76 different genera from the human gut, oceanic and soil

environments, respectively. For the purpose of this benchmarking, we simulated the datasets with a similar relative abundance per genus to avoid introducing any taxon-specific biases (Additional file: Fig. S1).

To simulate a realistic scenario, where variation can arbitrarily occur and sequences may not have an exact representative in public databases, we randomly mutated 2% of the positions of each 16S rRNA sequence retrieved. Using an *in silico* PCR, we then extracted each sub-region using commonly used primer sequences (Additional file: Table S1). Notably, the percentage of bacterial sequences from the Greengenes and SILVA databases matching the primers selected for V1-V2 was dramatically lower (37.6%) than that of V3-V4 (99.2%), V4 (99.1%) and V4-V5 (99.4%) (Additional file: Fig. S2). The 16S rRNA V1 sub-region had been previously found to be truncated in a substantial number of reference sequences [23]. Our results confirm this observation and again raise caution at the use of the 16S V1-V2 rRNA primer sequences for complex and diverse samples, due to the reduced number of reference sequences available.

Taxonomic assignment

Microbiome studies frequently strive to associate microbial diversity signatures with a phenotype of interest. However, focusing solely on high-level taxonomic ranks can severely underestimate the degree of variation observed between sample groups. To circumvent this, highly discriminative approaches are needed to be able to pinpoint the most significant taxa warranting further validation. For assessing the performance of MAPseq, mothur, QIIME and QIIME 2 with different reference databases (Additional file: Fig. S3), we limited our analyses to classification at the lineage level instead of operational taxonomic units (OTUs), as it allows a more consistent and easier interpretation of the results. Species assignment of every

queried sequence would be the desired outcome, but the limited resolution of the 16S rRNA locus precludes an accurate classification at this level. In fact, databases such as RDP do not report taxon names below genus. In this work, we calculated the degree of recall and precision at the genus and family ranks, as in our opinion they provide the best compromise between classification accuracy and resolution.

By comparing the level of recall across all software tools, we found that QIIME 2 recovered the largest proportion of sequences from the expected genera (Table 1 and Fig. 1). Combined with the SILVA database, this resulted in the highest recall (sensitivity) for human gut (67.1%) and soil samples (67.7%), while the Greengenes database outperformed in the case of the oceanic microbiome (79.8%). In fact, all tools except QIIME saw a decrease in recall when using SILVA specifically for the classification of the oceanic dataset. Globally, however, SILVA most frequently provided a better genus recall than Greengenes (five out of nine comparisons across MAPseq, QIIME and QIIME 2, Fig. 1). In terms of correctly identified taxa, MAPseq in conjunction with SILVA detected the greatest number of expected genera in all three biomes (Fig. 1). At the family level, all tools presented a substantially higher recall (Table 1), with QIIME 2 reaching 93.9% in the human gut sample, 95.8% with the ocean set and 91.3% with the soil sample.

Although the level of recall is a crucial metric in choosing the most appropriate taxonomic classification pipeline, it is equally important to ensure a low frequency of false-positive assignments. We evaluated the degree of precision (specificity) by the percentage of sequences assigned to the wrong taxon out of all the detected taxa. Accuracy was high for all the tools, with precision estimates of at least 85% across all analysis pipelines (Fig. 2A). In terms of total number of sequences, this translated to less than 9% of the reads misassigned at

the genus level (Additional files: Tables S2, S3, S4 and Fig. S4). MAPseq with the SILVA database consistently outperformed all other tools, with a precision above 96% for the three tested biomes (Fig. 2A), equating to less than 2% of miscalled sequences (Additional file: Fig. S4).

To combine both recall and precision into a single metric, we calculated the F-score for all taxonomic assignments (Fig. 2A and Additional file: Fig. S5). At both genus and family levels, we found that QIIME 2 had the highest score across the samples representative of the three different biomes, with the SILVA database coming out on top for the human gut (genus: 78.9%, family: 96.6%) and soil (genus: 78%, family: 94.1%) environments in particular, but the Greengenes database performing better with the oceanic dataset (genus: 87.6%, family: 97.2%). After fractioning the data according to different sub-regions of the 16S rRNA gene, we then repeated the same analysis (Fig. 2B). This revealed that the performance of each tool varied up to 35% depending on the 16S rRNA sub-region targeted. Notably, the V1-V2 or V3-V4 sub-regions performed the best across most of the pipelines (Fig. 2B). In our study, each synthetic species had a genetically close full-length 16S rRNA sequence represented in the databases, so our tests were probably not significantly affected by the reduced number of V1-V2 reference sequences available.

The ongoing surge in genome sequencing is producing thousands of novel sequences each year. Therefore, efficient tools that can scale up to provide analysis of tens of thousands of samples is increasingly important. With this in mind, we compared the computational performance of MAPseq, mothur, QIIME and QIIME 2 throughout the whole classification pipeline of our simulated datasets. We analysed average memory usage and CPU time across the three biomes for the processing and assignment of 3 million quality-filtered sequences

against the SILVA 128 database (Fig. 3). MAPseq was the most memory-efficient tool, with mothur, QIIME and QIIME 2 requiring over 72, 6 and 27 times more memory resources, respectively (Fig. 3A). CPU time of QIIME 2 was the highest, close to twice that of MAPseq, and over 200 times longer than QIIME, which was the fastest (Fig. 3B). Of note is that each pipeline has its own processing procedure; both the mothur and QIIME 2 pipelines included a de-replication step of the query sequences prior to taxonomic assignment, which substantially reduces the number of sequences used for classification.

Relative quantification and beta diversity

One of the main aspects of any microbiome-based analysis is the assessment of the differential abundance and beta diversity across a set of sample groups. In this respect, accurate estimation of the relative abundance of each taxon is essential to find statistically significant patterns. To assess how accurately each tool was able to predict taxa relative abundances in each sample, we calculated dissimilarity scores (DS) for each genus present in the simulated dataset (Fig. 4). Interestingly, QIIME 2 showed the most accurate prediction in relation to the true genera composition, with an average DS of 0.32 when used in conjunction with the SILVA database (Table 1). In terms of the reference database used, analyses carried out with SILVA consistently yielded more accurate predictions than with the Greengenes database. Substantial differences in accuracy were observed across different genera, with sequences from the *Paraprevotella* genus — frequently present in human gut samples — more accurately predicted, in contrast to those from *Hyphomicrobium*, *Thalassobacter* and *Verrucomicrobium* — commonly found in oceanic biomes — which had the worst results (Fig. 4). These genera might either be underrepresented in the reference databases, or have a high degree of conservation with other closely related taxa, making accurate taxonomic assignments more challenging.

For a global assessment of the beta diversity across samples, we performed a principal coordinates analysis (PCoA) and calculated both Bray-Curtis and Jaccard distances between the observed and expected results. Both distance methods represent complementary approaches, as the Bray-Curtis metric corresponds to a quantitative evaluation of the dissimilarity across samples, whereas the Jaccard index is a qualitative measure of community similarity. We found that samples analysed with QIIME 2 were the closest (i.e. had the lowest distance estimate) to the true simulated datasets, with minor differences between the use of SILVA or Greengenes with both the Bray-Curtis and Jaccard methods (Table 1; Fig. 5).

Discussion

With the number of tools, databases and options available for taxonomic classification of marker sequences, it can be a daunting task to decide the optimal approach for analysis of a specific dataset. In this work, we have strived to help guide this decision-making process by independently assessing the performance of the most commonly used taxonomic assignment strategies with simulated samples comprised of genera found in frequently sampled environments.

Overall, we show that all tools we tested performed moderately well, with high precision and modest-to-high recall rates at the genus level. QIIME 2 presents significant improvements over the other tools, particularly over the preceding version of QIIME, in regard to detection sensitivity at both family and genus level. The superiority of QIIME 2 also held true for the prediction of sample composition, as beta diversity estimates between the analysed and simulated communities were the closest using this method. Therefore, these data support the use of QIIME 2 to obtain the largest proportion of classified sequences at the most accurate

relative abundances. Nevertheless, the results did show MAPseq to be a more conservative but specific approach, meaning that fewer genera were misassigned. This tool also showed considerably better computational performance than QIIME 2, requiring approximately 30 times less memory and almost half the CPU time to process the same dataset (even though QIIME 2 classifies substantially fewer query sequences due to a prior de-replication step). These results show that MAPseq provides a credible option if precision and computational performance or scale are a priority. It should be noted that selecting a single best software package is not a straightforward affair, and we expect that differences in performance will be observed with different real-world datasets. Furthermore, aside from the software packages we tested, other web-based tools such as BioMaS [22] are also available. But, they are usually restricted to the use of specific reference databases, making individual customizations and accurate comparisons more challenging.

In addition to choosing the right tool, combining that with the appropriate reference database is equally important to ensure the best classification performance. Greengenes and SILVA have been the most widely-used and readily supported databases. Generally, the SILVA 128 database performed better than Greengenes 13_8 in terms of recall at both family and genus levels, as well as in predicting the true taxa composition of the simulated communities. Conversely, there was an almost universal decrease in its performance in the detection of ocean-specific taxa, so special care should be taken in the analysis of datasets sampled from this particular environment. Nonetheless, there are additional advantages to the use of SILVA: it is more frequently updated (Greengenes was last updated in May 2013); it includes rRNA sequences of eukaryotic organisms in addition to archaea and bacterial species; and has been shown to be more easily comparable and mapped to other taxonomies such as the NCBI [32]. In the case of MAPseq and mothur, the NCBI and RDP databases also performed

well, with higher recall but slightly lower precision scores compared to SILVA. Therefore, the SILVA, RDP or NCBI databases are all appropriate choices for a comprehensive and accurate taxonomic analyses.

The choice of primer sequences for taxonomic profiling of the 16S rRNA gene has been a matter of frequent debate. In common with previously reported observations [27], we show that targeting different sub-regions can considerably influence the taxonomic assignment performance (by up to 35% in our analyses). Overall, the V1-V2 and V3-V4 sub-regions performed the best across most of the tools. However, the V1-V2 primers did not match more than 60% of the reference sequences across SILVA and Greengenes, so we discourage its use for classification of complex community samples. As our simulated datasets were generated from close representatives containing full-length 16S rRNA genes, it is reasonable to assume that our analysis of the V1-V2 sub-region was not significantly hampered by this reduced number of reference sequences. Kozich *et al*. [24] have argued in favour of standardizing the use of the V4 sub-region for Illumina MiSeq sequencing, as it allows complete overlap of paired-end sequences, mitigating sequence errors introduced during PCR amplification or sequencing. Phylogenetic studies have also showed that the V4 sub-region is the closest representative of the phylogenetic signal of the whole 16S rRNA locus [23]. Here, we analysed the performance of some of the most commonly used sub-regions under a purely computational perspective, and conclude that amplification of the V3-V4 sub-region is most frequently the best option for a reliable taxonomic inference.

In summary, we have identified the major benefits and drawbacks of the most recent and popular taxonomic classification methods. Importantly, we show that the choice of software, database and sub-region significantly affects the quality of the classification results. Given

the impact of each of these variables, it is imperative to strive for consistency in the analysis of samples not only within individual studies, but across different projects as well. Services like the EBI metagenomics [7] and MG-RAST [33] help provide a basis for standardization, but additional factors relating to the experimental design are up to individual users to decide. Hence, in this fast-evolving field, we believe the work presented here will help the microbiome research community make more informed decisions about the most appropriate methodological approach to take in their own analysis pipeline.

Methods

Generating simulated datasets

Twelve sets of synthetic communities were generated for evaluating the accuracy of the taxonomic assignment pipelines: four each for human gut, ocean and soil environments. First, the 80 most abundant genera across publicly deposited samples from these biomes were retrieved using the EBI metagenomics API (https://www.ebi.ac.uk/metagenomics/api/) [7]. This list was then used to randomly select either 100 (datasets A100 and B100) or 500 species (datasets A500 and B500) belonging to these genera, allowing a maximum of 20 and 50 species per genus, respectively. 16S rRNA gene sequences were extracted from the European Nucleotide Archive (ENA) and 2% of the positions were randomly mutated to create nucleotide diversity, using a custom python script (https://github.com/Finn-Lab/Tax-Benchmarking). From these mutated sequences, an *in silico* PCR was carried out with an additional python script (https://github.com/simonrharris/in_silico_pcr), targeting commonly used regions for 16S rRNA profiling (Additional file: Table S1): V1-V2, V3-V4, V4 and V4-V5. Sequencing reads were simulated from these amplicon sequences in duplicate with ART [34], generating ~ 10 000 and ~ 200 000 paired-end reads of 250 bp per region to have samples representing both low and high levels of sequencing depth.

Sequence classification

Initial pre-processing and quality control was performed following the mothur standard operating procedure (SOP) [24], accessed on November 2017. Briefly, the *make.contigs* command was used to align, filter and merge the paired-end reads into contigs. Subsequently, we used the *screen.seqs* command to filter out any sequences with ambiguous base calls. This final set of quality controlled sequences was then assigned into taxonomic lineages with MAPseq v1.2.2 [17], mothur v1.39.5 [9], QIIME 1.9.1 [10], and QIIME 2 v2017.11 (https://qiime2.org/). For each software, we evaluated the settings and databases most frequently used and recommended for optimal taxonomic classification (Additional file: Fig. S3). With MAPseq, we tested the default NCBI database (mapref 2.2), as well as Greengenes 13_8 and the SILVA 128 database re-mapped to an eight-level taxonomy (available in ftp://ftp.ebi.ac.uk/pub/databases/metagenomics/mapseq_silva128). Each set of reference sequences was analysed following the internal clustering by MAPseq. Options *-tophits 80* and *-topotus 40* were used in combination with the *-outfmt simple* option. For QIIME 1.9.1, the *pick_closed_reference_otus.py* script was used with both the default Greengenes database (13_8) and with SILVA 128 clustered at 97% identity. Taxonomic assignment with mothur was carried out according to the MiSeq SOP [24], excluding the chimera detection and removal steps, using the available pre-formatted SILVA 128 database for alignment and either the RDP version 16 or SILVA 128 for sequence classification. Lastly, for QIIME 2 we first dereplicated the query sequences using the vsearch dereplicate-sequences function and then assigned them to the Greengenes (13_8) or SILVA 128 (99% identity clusters) databases using the feature-classifier classify-sklearn function [18].

Analysis and visualization

TSV and BIOM files were generated from the MAPseq and QIIME 2 outputs and combined with the output BIOM files created by QIIME and mothur (make.biom command). Taxonomy names obtained from each individual reference database were normalized so that each genus and family would be assigned to the same lineage. Results were visualized and analysed with the phyloseq [35] and vegan R packages. The recall rate (sensitivity) for each tool and database was estimated as the percentage of sequences assigned to the expected taxa for each biome, while precision (specificity) was calculated as the fraction of sequences from these predicted taxa out of all those from the taxa observed. Finally, the F-score was calculated as follows:

$$\mathrm{F\text{-}score} = 2 \times \frac{\mathrm{precision} \times \mathrm{recall}}{\mathrm{precision} + \mathrm{recall}}$$

Distance estimates were calculated with either the Bray-Curtis or Jaccard dissimilarity indices after grouping the taxonomic lineages at the genus level. Principal coordinate analysis (PCoA) were performed with the Bray-Curtis distance method. Dissimilarity scores (DS) on the relative abundance (rel.ab) of each expected genus were calculated as:

$$\mathrm{DS} = \frac{|\mathrm{rel.ab.(Observed)} - \mathrm{rel.ab.(Expected)}|}{\mathrm{rel.ab.(Expected)}}$$

Memory usage and CPU time was estimated as the total amount required for the processing and assignment of all combined sequences against the SILVA 128 database, following the protocols described above.

Availability of supporting source code and requirements

Project name: Taxonomy benchmarking

Project home page: https://github.com/Finn-Lab/Tax-Benchmarking

Operating system: Platform independent

Programming languages: Python 2.7, R 3.4.1

Other requirements: BioPython module, R libraries (ggplot2, phyloseq, vegan, scales, grid, ape, RColorBrewer, data.table)

License: MIT

Availability of supporting data

The datasets supporting the conclusions of this article are available in the GigaDB repository [36].

Declarations

List of abbreviations

DS: Dissimilarity score

GG: Greengenes

PCoA: Principal coordinates analysis

rRNA : ribosomal rRNA

Ethics approval and consent to participate

Not applicable

Consent for publication

Not applicable

Competing interests

The authors declare that they have no competing interests

Funding

European Molecular Biology Laboratory (EMBL); European Commission within the Research Infrastructures Programme of Horizon 2020 [676559] (ELIXIR-EXCELERATE)

Authors' contributions

AA, ALM, AT and RDF performed the analyses. AA, ALM and RDF conceived the study and wrote the manuscript. All authors have read and approved the final manuscript.

Acknowledgements

We thank João Matias Rodrigues for providing useful comments on the utility of MAPseq in relation to the benchmarking.

Figure legends

Figure 1. Level of recall at the genus level, represented as taxa relative abundances, obtained with each analysis pipeline for the three different biomes (human gut, ocean and soil). The number of genera correctly identified by each pipeline is indicated above the graph.

Figure 2. (A) Recall, precision and F-score estimates at the genus level for each tool and database tested. (B) F-scores calculated for some of the most commonly tested sub-regions of the 16S rRNA gene: V1-V2, V3-V4, V4 and V4-V5.

Figure 3. Computational cost of each taxonomy assignment tool, estimated as the total memory usage (A) and CPU time (B) required for the processing and classification of ~ 3 million sequences against the SILVA 128 database.

Figure 4. Dissimilarity scores (DS) calculated for each genus included in the simulated datasets. Lower (brighter) values indicate a closer prediction to the true composition of the original sample. The black outline indicates the overall best scoring analysis pipeline for each environment.

Figure 5. Principal coordinates analysis (PCoA) between all samples analysed in relation to the true, expected dataset, using the Bray-Curtis distance method.

Figure S1. Composition of the synthetic communities per selected environment. Samples A100 and B100 are randomly generated sets of 100 species, while A500 and B500 were simulated from 500 different species.

Figure S2. Percentage of bacterial sequences retrieved from the Greengenes and SILVA databases with an *in silico* PCR targeting different 16S rRNA gene sub-regions.

Figure S3. Tools and databases benchmarked in our study. We tested at least two databases per software tool. The reference databases used were either readily supported by the specific tool and/or recommended by their developers. SILVA was compared across all tools; MAPseq was specifically assessed with the NCBI database, its default reference; mothur was not paired with Greengenes due to its poor-quality alignment and was analysed with RDP instead.

Figure S4. Number of genera misassigned in each analysis pipeline and their overall relative abundance. Names and abundance values of each misclassified taxon are included as additional files (Additional files: Tables S2, S3 and S4).

Figure S5. Recall, precision and F-score estimates at the family level for each tool and database tested.

References

1. Forbes JD, Van Domselaar G, Bernstein CN. The gut microbiota in immune-mediated inflammatory diseases. Front. Microbiol. 2016.

2. Duvallet C, Gibbons SM, Gurry T, Irizarry RA, Alm EJ. Meta-analysis of gut microbiome studies identifies disease-specific and shared responses. Nat. Commun. Nature Publishing Group; 2017;8:1784.

3. Turnbaugh PJ, Ley RE, Mahowald MA, Magrini V, Mardis ER, Gordon JI. An obesity-associated gut microbiome with increased capacity for energy harvest. Nature. Nature Publishing Group; 2006;444:1027–131.

4. Thompson LR, Sanders JG, McDonald D, Amir A, Ladau J, Locey KJ, et al. A communal catalogue reveals Earth's multiscale microbial diversity. Nature. 2017;551:457–63.

5. Yilmaz P, Yarza P, Rapp JZ, Glöckner FO. Expanding the World of Marine Bacterial and Archaeal Clades. Front. Microbiol. 2016;6:1524.

6. Fierer N. Embracing the unknown: disentangling the complexities of the soil microbiome. Nat. Rev. Microbiol. Nature Publishing Group; 2017;15:579–90.

7. Mitchell AL, Scheremetjew M, Denise H, Potter S, Tarkowska A, Qureshi M, et al. EBI Metagenomics in 2017: enriching the analysis of microbial communities, from sequence reads to assemblies. Nucleic Acids Res. 2017;

8. Pace NR, Stahl DA, Lane DJ, Olsen GJ. The Analysis of Natural Microbial Populations by Ribosomal RNA Sequences. Adv. Microb. Ecol. Springer, Boston, MA; 1986. p. 1–55.

9. Schloss PD, Westcott SL, Ryabin T, Hall JR, Hartmann M, Hollister EB, et al. Introducing mothur: open-source, platform-independent, community-supported software for describing and comparing microbial communities. Appl. Environ. Microbiol. American Society for Microbiology; 2009;75:7537–41.

10. Caporaso JG, Kuczynski J, Stombaugh J, Bittinger K, Bushman FD, Costello EK, et al.

QIIME allows analysis of high-throughput community sequencing data. Nat. Methods. Nature Publishing Group; 2010;7:335–6.

11. McDonald D, Price MN, Goodrich J, Nawrocki EP, DeSantis TZ, Probst A, et al. An improved Greengenes taxonomy with explicit ranks for ecological and evolutionary analyses of bacteria and archaea. ISME J. Nature Publishing Group; 2012;6:610–8.

12. Federhen S. The NCBI Taxonomy database. Nucleic Acids Res. Oxford University Press; 2012;40:D136-43.

13. Cole JR, Wang Q, Fish JA, Chai B, McGarrell DM, Sun Y, et al. Ribosomal Database Project: data and tools for high throughput rRNA analysis. Nucleic Acids Res. Oxford University Press; 2014;42:D633–42.

14. Yilmaz P, Parfrey LW, Yarza P, Gerken J, Pruesse E, Quast C, et al. The SILVA and "All-species Living Tree Project (LTP)" taxonomic frameworks. Nucleic Acids Res. Oxford University Press; 2014;42:D643-8.

15. Edgar RC. Search and clustering orders of magnitude faster than BLAST. Bioinformatics. Oxford University Press; 2010;26:2460–1.

16. Wang Q, Garrity GM, Tiedje JM, Cole JR. Naive Bayesian classifier for rapid assignment of rRNA sequences into the new bacterial taxonomy. Appl. Environ. Microbiol. American Society for Microbiology (ASM); 2007;73:5261–7.

17. Matias Rodrigues JF, Schmidt TSB, Tackmann J, von Mering C. MAPseq: highly efficient k-mer search with confidence estimates, for rRNA sequence analysis. Bioinformatics. 2017;

18. Bokulich NA, Kaehler BD, Rideout JR, Dillon M, Bolyen E, Knight R, et al. Optimizing taxonomic classification of marker gene amplicon sequences. PeerJ (preprint). 2018;

19. Sczyrba A, Hofmann P, Belmann P, Koslicki D, Janssen S, Dröge J, et al. Critical Assessment of Metagenome Interpretation—a benchmark of metagenomics software. Nat.

Methods. Nature Publishing Group; 2017;14:1063–71.

20. Golob JL, Margolis E, Hoffman NG, Fredricks DN. Evaluating the accuracy of amplicon-based microbiome computational pipelines on simulated human gut microbial communities. BMC Bioinformatics. BioMed Central; 2017;18:283.

21. Lindgreen S, Adair KL, Gardner PP. An evaluation of the accuracy and speed of metagenome analysis tools. Sci. Rep. 2015;

22. Fosso B, Santamaria M, Marzano M, Alonso-Alemany D, Valiente G, Donvito G, et al. BioMaS: a modular pipeline for Bioinformatic analysis of Metagenomic AmpliconS. BMC Bioinformatics. BioMed Central; 2015;16:203.

23. Yang B, Wang Y, Qian P-Y. Sensitivity and correlation of hypervariable regions in 16S rRNA genes in phylogenetic analysis. BMC Bioinformatics. 2016;

24. Kozich JJ, Westcott SL, Baxter NT, Highlander SK, Schloss PD. Development of a Dual-Index Sequencing Strategy and Curation Pipeline for Analyzing Amplicon Sequence Data on the MiSeq Illumina Sequencing Platform. Appl. Environ. Microbiol. 2013;

25. Klindworth A, Pruesse E, Schweer T, Rg Peplies J, Quast C, Horn M, et al. Evaluation of general 16S ribosomal RNA gene PCR primers for classical and next-generation sequencing-based diversity studies. Nucleic Acids Res. 2012;41:e1.

26. Walker AW, Martin JC, Scott P, Parkhill J, Flint HJ, Scott KP. 16S rRNA gene-based profiling of the human infant gut microbiota is strongly influenced by sample processing and PCR primer choice. Microbiome. BioMed Central; 2015;3:26.

27. Comeau AM, Douglas GM, Langille MGI. Microbiome Helper: a Custom and Streamlined Workflow for Microbiome Research. Eisen J, editor. mSystems. American Society for Microbiology Journals; 2017;2:e00127-16.

28. Brooks JP, Edwards DJ, Harwich MD, Rivera MC, Fettweis JM, Serrano MG, et al. The truth about metagenomics: quantifying and counteracting bias in 16S rRNA studies. BMC

Microbiol. 2015;15:66.

29. Schloss PD, Gevers D, Westcott SL. Reducing the effects of PCR amplification and sequencing Artifacts on 16s rRNA-based studies. PLoS One. 2011;6.

30. Quince C, Walker AW, Simpson JT, Loman NJ, Segata N. Shotgun metagenomics, from sampling to analysis. Nat. Biotechnol. 2017;35:833–44.

31. Shakya M, Quince C, Campbell JH, Yang ZK, Schadt CW, Podar M. Comparative metagenomic and rRNA microbial diversity characterization using archaeal and bacterial synthetic communities. Environ. Microbiol. NIH Public Access; 2013;15:1882–99.

32. Balvočiute M, Huson DH. SILVA, RDP, Greengenes, NCBI and OTT — how do these taxonomies compare? BMC Genomics. 2017;18.

33. Keegan KP, Glass EM, Meyer F. MG-RAST, a Metagenomics Service for Analysis of Microbial Community Structure and Function. Methods Mol. Biol. 2016. p. 207–33.

34. Huang W, Li L, Myers JR, Marth GT. ART: a next-generation sequencing read simulator. Bioinformatics. 2012;28:593–59410.

35. McMurdie PJ, Holmes S, Watson M. phyloseq: An R Package for Reproducible Interactive Analysis and Graphics of Microbiome Census Data. Watson M, editor. PLoS One. Public Library of Science; 2013;8:e61217.

36. Sneddon TP, Li P, Edmunds SC. GigaDB: announcing the GigaScience database. Gigascience. Oxford University Press; 2012;1:11.

Table 1. Global metrics averaged across the analyses of simulated samples from human gut, ocean and soil.

		Family		Genus					
**Software**	**Database**	**Recall**	**Miscalled**	**Recall**	**Miscalled**	**Sub-region[1]**	**Mean DS**	**Bray-Curtis**	**Jaccard**
MAPseq	Greengenes	87.8	2.4	58.6	2.5	V3-V4	0.435	0.284	0.441
MAPseq	NCBI	81.4	1.3	51.2	1.9	V3-V4	0.523	0.333	0.499
MAPseq	SILVA	66.9	**0.7**	46.2	**0.9**	V3-V4	0.484	0.375	0.543
mothur	RDP	84.8	3.2	49.2	4.8	V4-V5	0.430	0.364	0.532
mothur	SILVA	82.2	2.2	40.1	4.5	V4-V5	0.493	0.449	0.617
QIIME 2	Greengenes	92.5	1.8	69.1	3.4	V3-V4	0.372	0.211	**0.344**
QIIME 2	SILVA	**93.3**	1.9	**69.2**	4.4	V3-V4	**0.323**	**0.209**	0.345
QIIME	Greengenes	55.6	1.3	42.3	1.9	V4	0.619	0.420	0.592
QIIME	SILVA	61.8	2.1	53.3	6.4	V3-V4	0.473	0.343	0.508

Values in bold denote the best score.

[1]Sub-region with the highest F-score, excluding V1-V2.

Figure 1

Click here to download Figure Figure_1.pdf

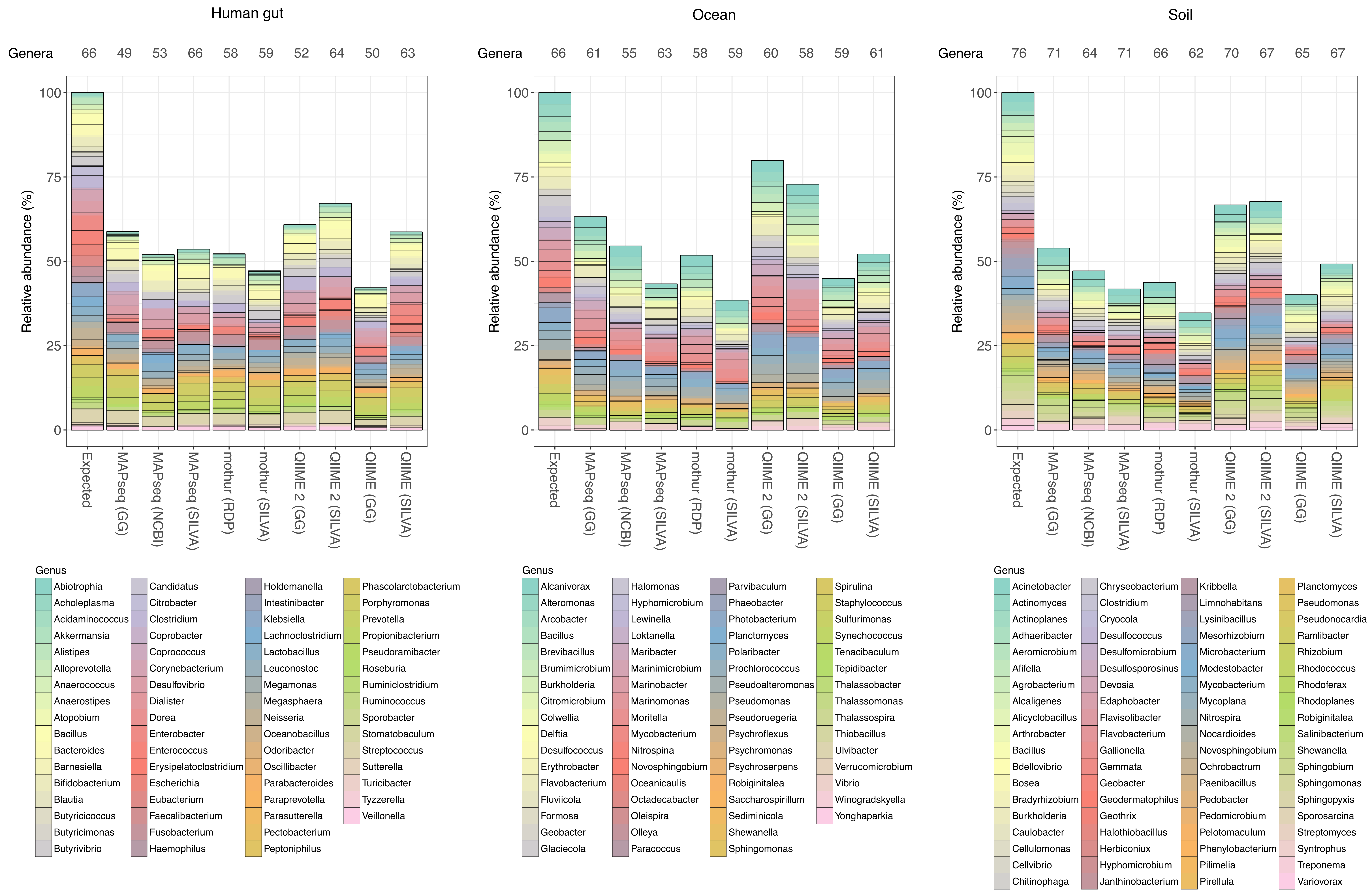

Figure 2

Click here to download Figure Figure_2.pdf

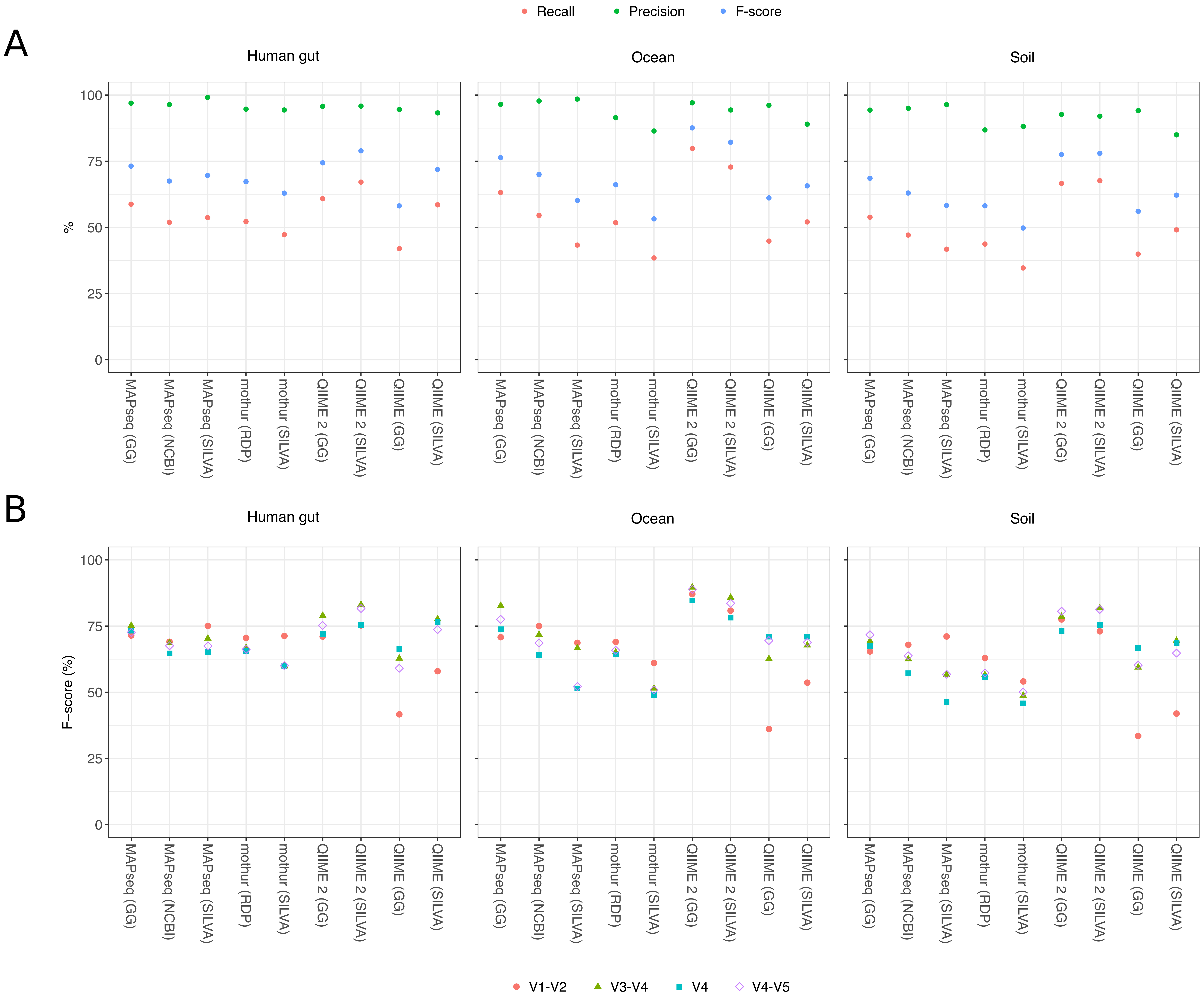

Figure 3

Click here to download Figure Figure_3.pdf

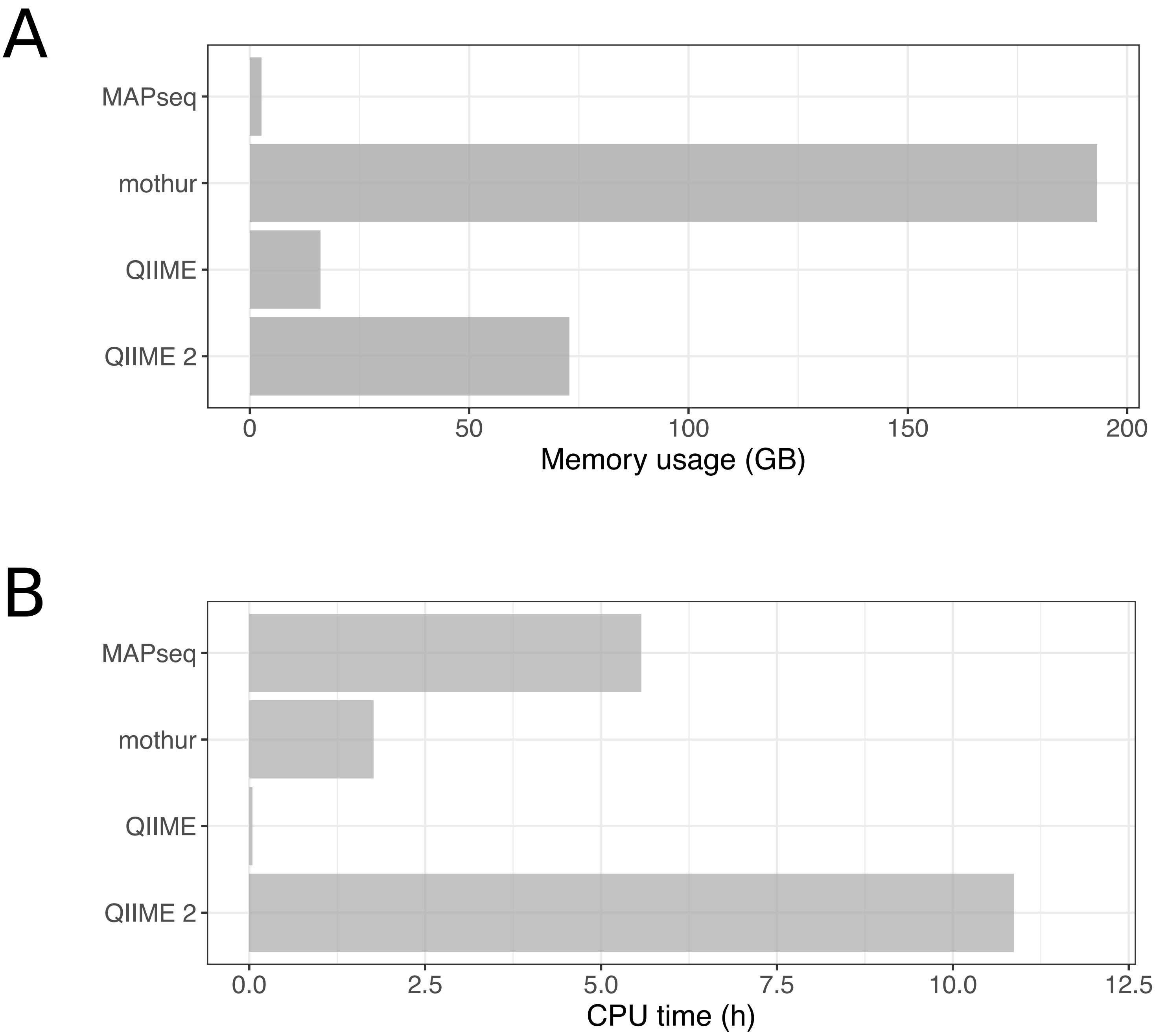

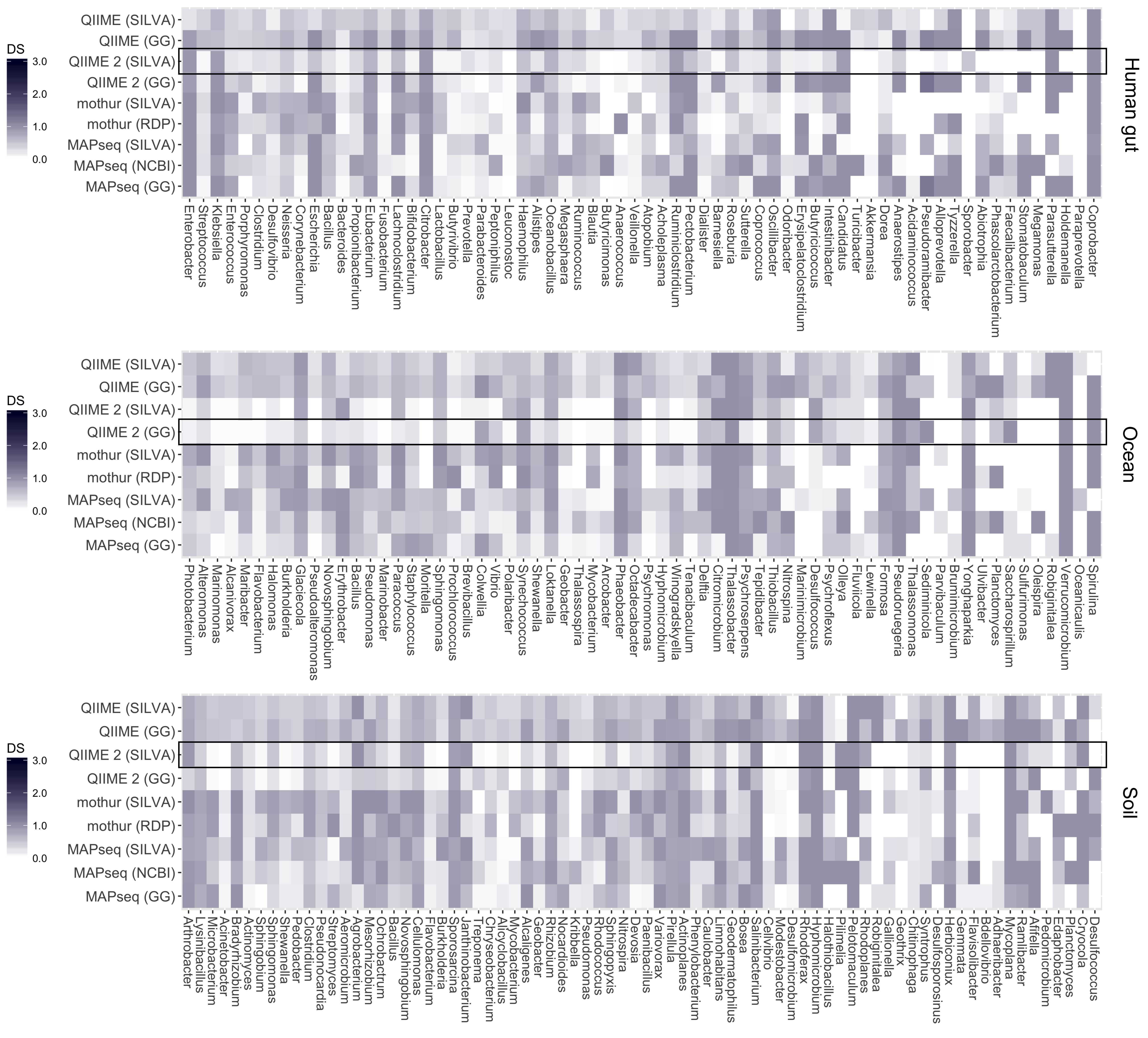
DS
3.0
2.0
1.0
0.0
QIIME (SILVA)
QIIME (GG)
QIIME 2 (SILVA)
QIIME 2 (GG)
mothur (SILVA)
mothur (RDP)
MAPseq (SILVA)
MAPseq (NCBI)
MAPseq (GG)
Human gut
Enterobacter Streptococcus Klebsiella Enterococcus Porphyromonas Clostridium Desulfovibrio Neisseria Corynebacterium Escherichia Bacillus Bacteroides Propionibacterium Eubacterium Fusobacterium Lachnoclostridium Bifidobacterium Citrobacter Lactobacillus Butyrivibrio Prevotella Parabacteroides Peptoniphilus Leuconostoc Haemophilus Alistipes Oceanobacillus Megasphaera Ruminococcus Blautia Butyricimonas Anaerococcus Veillonella Atopobium Acholeplasma Ruminiclostridium Pectobacterium Dialister Barnesiella Roseburia Sutterella Coprococcus Oscillibacter Odoribacter Erysipelatoclostridium Butyricicoccus Intestinibacter Candidatus Turicibacter Akkermansia Dorea Anaerostipes Acidaminococcus Pseudoramibacter Alloprevotella Tyzzerella Sporobacter Abiotrophia Phascolarctobacterium Faecalibacterium Stomatobaculum Megamonas Parasutterella Holdemanella Paraprevotella Coprobacter
Ocean
Photobacterium Alteromonas Marinomonas Alcanivorax Maribacter Flavobacterium Halomonas Burkholderia Glaciecola Pseudoalteromonas Novosphingobium Erythrobacter Bacillus Pseudomonas Marinobacter Paracoccus Staphylococcus Moritella Sphingomonas Prochlorococcus Brevibacillus Colwellia Vibrio Polaribacter Synechococcus Shewanella Loktanella Geobacter Thalassospira Mycobacterium Arcobacter Phaeobacter Octadecabacter Psychromonas Hyphomicrobium Winogradskyella Tenacibaculum Delftia Citromicrobium Thalassobacter Psychroserpens Tepidibacter Thiobacillus Nitrospina Marinimicrobium Desulfococcus Psychroflexus Olleya Fluviicola Lewinella Formosa Pseudoruegeria Thalassomonas Sediminicola Parvibaculum Brumimicrobium Yonghaparkia Ulvibacter Planctomyces Saccharospirillum Sulfurimonas Oleispira Robiginitalea Verrucomicrobium Oceanicaulis Spirulina
Soil
Arthrobacter Lysinibacillus Microbacterium Acinetobacter Bradyrhizobium Actinomyces Sphingobium Sphingomonas Shewanella Pedobacter Clostridium Pseudonocardia Streptomyces Aeromicrobium Agrobacterium Mesorhizobium Ochrobactrum Bacillus Novosphingobium Cellulomonas Flavobacterium Burkholderia Sporosarcina Janthinobacterium Treponema Chryseobacterium Alicyclobacillus Mycobacterium Alcaligenes Geobacter Rhizobium Nocardioides Kribbella Pseudomonas Rhodococcus Sphingopyxis Nitrospira Devosia Paenibacillus Variovorax Pirellula Actinoplanes Phenylobacterium Caulobacter Limnohabitans Geodermatophilus Bosea Salinibacterium Cellvibrio Modestobacter Desulfomicrobium Rhodoferax Hyphomicrobium Halothiobacillus Pilimelia Pelotomaculum Rhodoplanes Robiginitalea Gallionella Geothrix Chitinophaga Syntrophus Desulfosporosinus Herbiconiux Gemmata Flavisolibacter Bdellovibrio Adhaeribacter Mycoplana Ramlibacter Afifella Pedomicrobium Edaphobacter Planctomyces Cryocola Desulfococcus

Figure 5

Click here to download Figure Figure_5.pdf

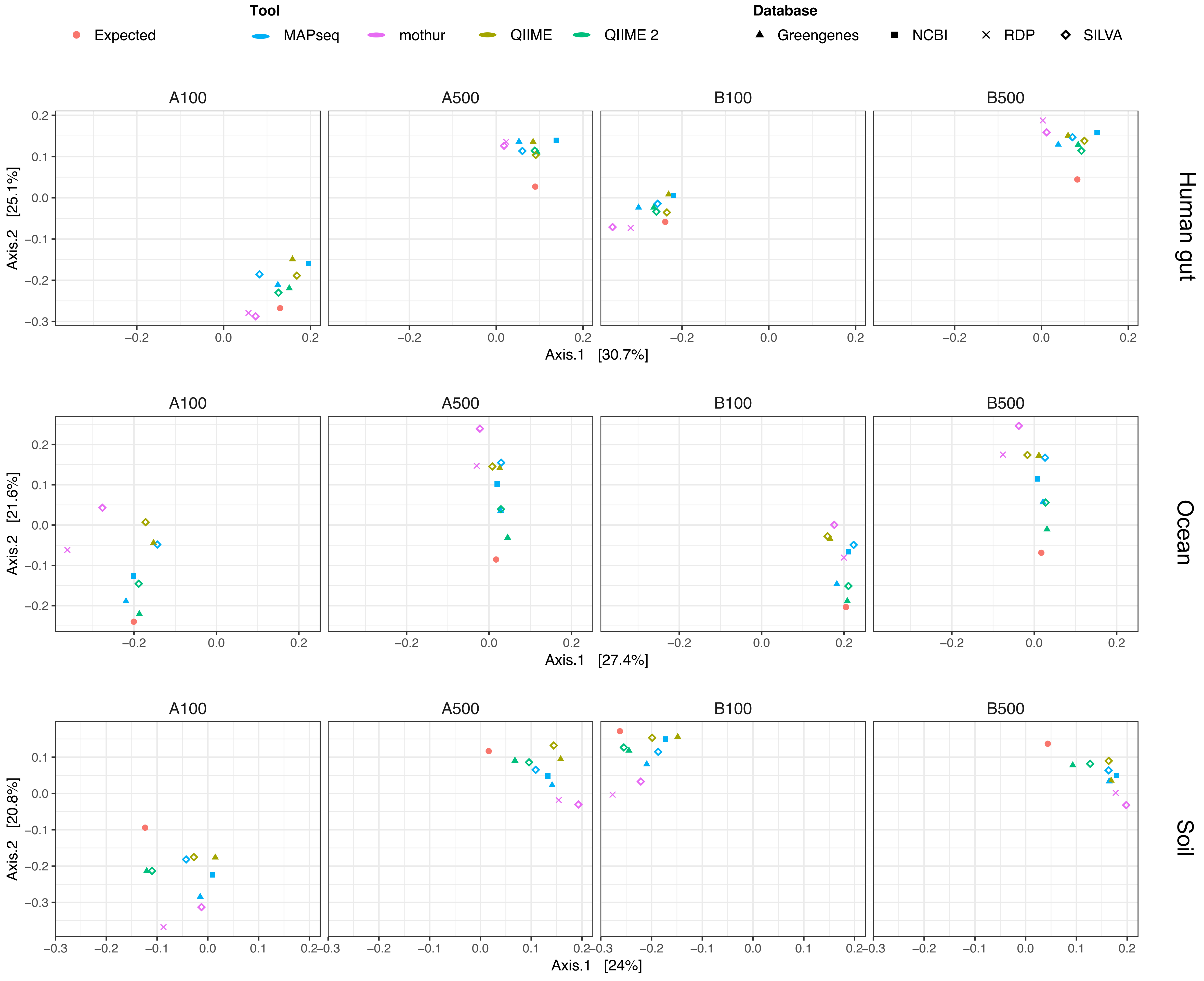

Additional file: Table S1

Click here to access/download
Supplementary Material
Table_S1.xlsx

Additional file: Table S2

Click here to access/download
Supplementary Material
Table_S2.xlsx

Additional file: Table S3

Click here to access/download
Supplementary Material
Table_S3.xlsx

Additional file: Table S4

Click here to access/download
Supplementary Material
Table_S4.xlsx

Additional file: Figure S1

Click here to access/download
Supplementary Material
Figure_S1.pdf

Additional file: Figure S2

Click here to access/download
Supplementary Material
Figure_S2.pdf

Additional file: Figure S3

Click here to access/download

Supplementary Material

Figure_S3.pdf

Additional file: Figure S4

Click here to access/download
Supplementary Material
Figure_S4.pdf

Additional file: Figure S5

Click here to access/download
Supplementary Material
Figure_S5.pdf
