# Supplementary material for: Benchmarking taxonomic assignments based on 16S rRNA gene profiling of the microbiota from commonly sampled environments
Source: Gigascience. 2018 May 11;7(5):giy054. doi: 10.1093/gigascience/giy054 (PMC5967554; doi:10.1093/gigascience/giy054)
Supplement: GIGA-D-18-00043_R1.pdf [file giy054_giga-d-18-00043_r1.pdf]

GigaScience

Benchmarking taxonomic assignments based on 16S rRNA gene profiling of the microbiota from commonly sampled environments

--Manuscript Draft--

Manuscript Number:	GIGA-D-18-00043R1
Full Title:	Benchmarking taxonomic assignments based on 16S rRNA gene profiling of the microbiota from commonly sampled environments
Article Type:	Technical Note
Funding Information:	European Bioinformatics Institute — Dr Alexandre Almeida<br>H2020 Research Infrastructures (676559) — Not applicable
Abstract:	Background: Taxonomic profiling of ribosomal RNA (rRNA) sequences has been the accepted norm for inferring the composition of complex microbial ecosystems. QIIME and mothur have been the most widely used taxonomic analysis tools for this purpose, with MAPseq and QIIME 2 being two recently released alternatives. However, no independent and direct comparison between these four main tools has been performed. Here, we compared the default classifiers of MAPseq, mothur, QIIME, and QIIME 2 using synthetic simulated datasets comprised of some of the most abundant genera found in the human gut, ocean and soil environments. We evaluate their accuracy when paired with both different reference databases and variable sub-regions of the 16S rRNA gene.<br><br>Findings: We show that QIIME 2 provided the best recall and F-scores at genus and family levels, together with the lowest distance estimates between the observed and simulated samples. However, MAPseq showed the highest precision, with miscall rates consistently below 2%. Notably, QIIME 2 was the most computationally expensive tool, with CPU time and memory usage almost two and 30 times higher than MAPseq, respectively. Using the SILVA database generally yielded a higher recall than using Greengenes, while assignment results of different 16S rRNA variable sub-regions varied up to 40% between samples analysed with the same pipeline.<br><br>Conclusions: Our results support the use of either QIIME 2 or MAPseq for optimal 16S rRNA gene profiling, and we suggest that the choice between the two should be based on the level of recall, precision and/or computational performance required.
Corresponding Author:	Alexandre Almeida<br><br>UNITED KINGDOM
Corresponding Author Secondary Information:	
Corresponding Author's Institution:	
Corresponding Author's Secondary Institution:	
First Author:	Alexandre Almeida
First Author Secondary Information:	
Order of Authors:	Alexandre Almeida<br>Alex L Mitchell<br>Aleksandra Tarkowska<br>Robert D Finn
Order of Authors Secondary Information:	
Response to Reviewers:	Reviewer #1<br>========

Powered by Editorial Manager® and ProduXion Manager® from Aries Systems Corporation

Independent benchmarks like this are important for guiding methods choices for researchers. I enjoyed reading this study and feel that it will be valuable to readers. I have a few questions and suggestions below.

R: We appreciate the positive feedback from the reviewer and have addressed all his comments and suggestions below and in the revised manuscript.

ln 20 - QIIME, mothur, and QIIME2 all utilize multiple different taxonomic classifiers. So multiple choices exist within each platform, there is no standard "mothur" or "QIIME" method (their defaults are essentially RDP classifier and a uclust-based classifier). It would be helpful to clarify this information in the text if not the abstract, e.g., the mothur classifier should be called RDP.

R: Based on both reviewers' comments, we have clarified this in the "Abstract" (lines 23-26), "Background" (lines 100-102) and "Discussion" sections (lines 262-264).

ln 29 - QIIME2 also appears to have higher F-measure scores, perhaps this should be mentioned here.

R: We have now included this information in the "Abstract" (lines 29-31).

ln 77-79 - what about the QIIME2 pre-print cited below? it does not cover Mapseq but is a benchmark of a number of different commonly used classifiers and marker-gene regions (albeit not an independent comparison).

R: We have now referenced this paper in the revised manuscript (lines 79-80).

ln 91-93 - what about the strengths of mock communities/weaknesses of simulation? this section seems to imply that mock communities are necessarily inferior, and simulations are not prone to their own limitations.

R: We agree with the reviewer and have now highlighted the importance of using both mock communities and in silico approaches in the "Background" section (lines 91-98).

ln 121 - variation is realistic, but not entirely random variation. Why not mutate simulated sequences after extracting the variable regions? It seems that much of the variation may otherwise fall outside of the variable regions and not impact this simulation.

R: We agree with the reviewer and have re-generated our simulated reads by mutating the sequences after extracting the variable regions instead. The new results are depicted in the revised version of the manuscript but show no significant differences to the original results presented. We have also replaced the original FASTQ files with this new set in the GigaDB FTP site.

ln 141-143 - why not at least show species-level results in the supplement if not main text? It is important to demonstrate why researchers should be cautious about species-level classifications.

R: We decided not to present the species-level assignment due to two main reasons: i) it has already been previously described (Golob, et al. 2017, PMID: 28558684) that 16S rRNA gene classification with amplicon-based sequences is severely limited at the species level, with only ~ 12% of correctly assigned sequences, and miscall rates of ~ 20%; ii) there is significant inconsistency in species nomenclature between the databases we tested (e.g. many species are just labelled "[Genus] sp.", whereas the RDP database does not even output species assignments), which would make an assessment of recall/precision challenging and possibly misleading, especially given the low number of assigned sequences. We have now made this clearer in the revised manuscript as well (lines 149-155).

ln 242 - parameter selection will greatly impact precision/recall scores, and e.g. increasing confidence thresholds for QIIME2 or mothur classifiers will improve precision at the expense of recall. Mapseq may have similar performance tradeoffs —

Powered by Editorial Manager® and ProduXion Manager® from Aries Systems Corporation

but overall I wonder if altering confidence thresholds for these other methods can approach the miscall rate of mapseq. At the very least, this should be mentioned in the discussion. The QIIME2 classifier pre-print cited by this work covers parameter permutations that maximize recall/precision (the default maximizes F-measure).

R: In this paper the aim was to assess the recommended and most widely-used parameters for each tool, as these will be what most users will likely be using in their analyses. We agree that tweaking individual settings might provide improvements to recall/precision estimates for each pipeline, but this was beyond the scope of this work and would have increased the number of comparisons performed exponentially. Therefore, as per the reviewer's suggestion we have now discussed this topic in the "Discussion" section of the revised manuscript (lines 262-264).

Reviewer #2
========

Overview

In this paper the authors have sought to evaluate the performance of the 4 main packages and their default classifiers/settings used in the taxonomic profiling of rRNA sequences. They did this using synthetic simulated read sets representative of 3 commonly studied microbiome environments and investigated the role of locus and reference database selection on classification metrics.

This is well done research that will form a useful benchmark for researchers engaged in rRNA taxonomic profiling to help design and conduct their own studies.

R: We thank the reviewer's positive remarks and have addressed all his comments below and in the revised manuscript.

General Comments

It should be emphasised throughout the manuscript that as of January 1st 2018, QIIME1 is deprecated and no longer supported by the developers (https://qiime.wordpress.com/2018/01/03/qiime-2-has-succeeded-qiime-1/). Therefore, QIIME1 is no longer recommended to be used at all.

R: We have now mentioned this in the "Background" (lines 69-71) and "Discussion" sections (lines 246-248).

Secondly, it is probably worth emhpasising that QIIME1, QIIME2 and mothur are very large toolsets with many parts and functions capable of more than just taxonomic assignment. Even for taxonomic assignment specifically, it could do with being clarified that mothur (RDP port, k-nearest neighbours, wang k-mer method) and QIIME (UCLUST, RDP, rtax, sortmerna, mothur's methods etc) implement a variety of optional alternative taxonomic classifiers. Comparing the performance of the default classifiers with default settings is very useful as that is what most users will end up using but it should be made clear in the manuscript that this work doesn't investigate these package options beyond database selection.

R: We have now included this information in the "Background" (lines 60-64) and "Discussion" sections (lines 262-264).

Minor Comments

Line 59: Possibly should be emhpasised that mothur, QIIME, and QIIME2 are large packages with lots of functions and uses beyond taxonomic assignment.

R: We have now added this information to the revised manuscript (lines 60-62).

Line 68: Although the RDP classifier can also be used optionally within QIIME fairly easily (although as the authors have stated is not default).

Powered by Editorial Manager® and ProduXion Manager® from Aries Systems Corporation

	R: We have now mentioned the possibility of using different classifiers in the "Discussion" section (lines 262-264).<br><br>Line 69: Mothur doesn't wrap RDP but totally reimplements RDP in C++ (http://blog.mothur.org/2016/01/12/mothur-and-qiime/)<br><br>R: We have now clarified this as per the reviewer's suggestion (lines 67-69).<br><br>Line 70: Worth highlighting that QIIME2 is intended to totally replace QIIME.<br><br>R: As mentioned above, we have now included this information in both the "Background" (lines 69-71) and "Discussion" sections (lines 246-248).<br><br>Line 124: Please add a citation for these primers if possible.<br><br>R: References for each primer set have now been added to the text (lines 129 and 329) and the accompanying supplementary table (Table S1).<br><br>Line 125: Can you clarify why RDP and MAPseq NCBI databases weren't used in this primer analysis?<br><br>R: We initially decided to focus on SILVA and Greengenes since they are most frequently used databases. However, we have now included the results for RDP and NCBI as well in the revised manuscript (lines 129-139 and Fig. S2).<br><br>Line 143: Has anyone done an analysis supporting the too limited resolution of this locus for species level classification?<br><br>R: In another benchmarking paper (Golob, et al. 2017, PMID: 28558684) it was shown that QIIME and mothur can only assign ~ 12% of 16S rRNA amplicon sequences to the correct species, while additionally presenting a miscall rate of ~ 21%. We have now cited this reference in the revised manuscript (line 150).<br><br>Line 151: Can you add the microbiome environment specific performance metrics for each tool as a (possibly supplemental) table instead of just the averaged metrics as report in Table 1? Acknowledging this involves some degree of overlap/redundancy to Figure 2.<br><br>R: We have now provided this information in three new supplementary tables (Tables S2, S3 and S4).<br><br>Line 208: As with the previous comment, despite the more detailed heatmap breakdown in Figure 4. It would be nice to see the overall dissimilarity metrics presented unaggregated by method and biome in a supplemental table.<br><br>R: This information has now also been added to the above-mentioned tables (Tables S2, S3 and S4).<br><br>Line 238: It might be good to further emphasis that is support the developer's decision to no longer support QIIME v1, especially with the tendency of outdated bioinformatics to linger and be widely used!<br><br>R: As stated above, we have now mentioned this in the "Background" (lines 69-71) and "Discussion" sections (lines 246-248).<br><br>Line 246: Do you believe this is likely to be due to overhead from QIIME2's zipping and unzipping of input files?<br><br>R: From our experience, QIIME 2's computational demand appears to be more significantly affected by the size of the database. It is possible that this is influenced by the uncompressing and compressing of the QZA files (the proprietary format used by QIIME 2), but we prefer not to speculate on this matter.<br><br>Line 251: Could add emphasis that these unevaluated alternatives includes other

	classifiers and settings within the software packages that were tested in this paper.<br><br>R: We have now mentioned this in the "Discussion" (lines 262-264).<br><br>Line 312: Using this script's default maximum primer mismatch of 3?<br>Line 315: What platform error profile was used when simulating reads with ART? MSv3?<br><br>R: Yes, we used the default primer mismatch of 3 and the MiSeq v3 error profile with ART. We have added this information to the "Methods" section (lines 326-335).<br><br>Line 337: Why was 99% clustered SILVA used for QIIME2 but 97% for QIIME1?<br><br>R: QIIME by default makes use of the Greengenes database clustered at 97%. To make a fair comparison across QIIME, we decided to cluster the SILVA database at the same level. On the other hand, the tutorials and standard operating procedures (SOP) of QIIME 2 advise and provide pre-trained databases of Greengenes and SILVA only at 99%. We hypothesize that these differences in the preferred clustering threshold might be related to the distinct assignment pipelines and default methods between the tools (UCLUST in QIIME vs. the Naïve Bayes classifier in QIIME 2).<br><br>Line 361: Presumably on a system under no other load? Was this run once or rerun a few times to determine variance of memory/cpu usage?<br><br>R: To assess the computational cost we calculated the average CPU time and memory usage across three different data points (one for each biome) after running each analysis in our cluster here at the EBI (which allocates the resources required for each job). We have now added error bars with the standard deviation to Fig. 3, showing the high consistency of these measurements.<br><br>References: Inconsistent capitalisation of titles, inclusion of editors and publisher information (mainly Nature Publishing Group) but others from the same publisher don't e.g. ref 4.<br><br>R: We have now corrected these formatting issues.<br><br>Figure 3 Legend: Is the SILVA database referenced here at different 97-99% clustering levels mentioned?<br><br>R: In the original manuscript we used the 97% clustered SILVA database for QIIME and the 99% one for QIIME 2. We realized that for assessing the computational cost this might be misleading, so we have now modified the analyses to use the same SILVA database across all comparisons (at a 99% clustering threshold). We have now also clarified this in the text (lines 380-382).<br><br>Figure S3: Explain and/or cite not using greengenes due to the alignment issue? It does seem not recommended. The methods section may benefit from inclusion of this database information.<br><br>R: We have now included this information in the revised manuscript (lines 355-356) and in the Fig. S3 legend (lines 457-458), with a citation to the mothur SOP.<br><br>Figure S4: Would be nice to include a key as per Figure 1 instead of needing to cross-reference to the tables.<br><br>R: Although we agree with the reviewer, given that the miscalled taxa correspond to over 100 different genera, it would be very challenging to have a figure key with discernible colours for each genus (especially given how small some of the stacked bars are). We realize it is not an ideal solution, but we have decided to leave that information as separate supplementary tables (now Tables S5, S6 and S7).
**Additional Information:**	
**Question**	**Response**

Powered by Editorial Manager® and ProduXion Manager® from Aries Systems Corporation

Are you submitting this manuscript to a special series or article collection?	No
**Experimental design and statistics**<br><br>Full details of the experimental design and statistical methods used should be given in the Methods section, as detailed in our Minimum Standards Reporting Checklist. Information essential to interpreting the data presented should be made available in the figure legends.<br><br>Have you included all the information requested in your manuscript?	Yes
**Resources**<br><br>A description of all resources used, including antibodies, cell lines, animals and software tools, with enough information to allow them to be uniquely identified, should be included in the Methods section. Authors are strongly encouraged to cite Research Resource Identifiers (RRIDs) for antibodies, model organisms and tools, where possible.<br><br>Have you included the information requested as detailed in our Minimum Standards Reporting Checklist?	Yes
**Availability of data and materials**<br><br>All datasets and code on which the conclusions of the paper rely must be either included in your submission or deposited in publicly available repositories (where available and ethically appropriate), referencing such data using a unique identifier in the references and in the "Availability of Data and Materials" section of your manuscript.<br><br>Have you have met the above requirement as detailed in our Minimum Standards Reporting Checklist?	Yes

Powered by Editorial Manager® and ProduXion Manager® from Aries Systems Corporation

Manuscript Click here to download Manuscript Manuscript(1).docx

Benchmarking taxonomic assignments based on 16S rRNA gene profiling of the microbiota from commonly sampled environments

Alexandre Almeida[1,2,*], Alex L. Mitchell[1], Aleksandra Tarkowska[1] and Robert D. Finn[1]

[1]EMBL-EBI European Bioinformatics Institute, Wellcome Genome Campus, Hinxton, Cambridge CB10 1SD, UK; [2]Wellcome Trust Sanger Institute, Wellcome Genome Campus, Hinxton CB10 1SA, UK

[*]Corresponding author:
Alexandre Almeida,
EMBL-EBI European Bioinformatics Institute,
Wellcome Trust Genome Campus, Hinxton, Cambridge CB10 1SD, UK
Tel + 44 (0) 1223 494 468 E-mail: aalmeida@ebi.ac.uk

ORCID IDs: Alexandre Almeida: 0000-0001-8803-0893; Alex L. Mitchell: 0000-0001-8655-7966; Aleksandra Tarkowska: 0000-0002-3392-3691; Robert D. Finn: 0000-0001-8626-2148

Keywords: 16S rRNA gene, human gastrointestinal tract, ocean, microbiome, soil, taxonomy

Abstract

Background: Taxonomic profiling of ribosomal RNA (rRNA) sequences has been the accepted norm for inferring the composition of complex microbial ecosystems. QIIME and mothur have been the most widely used taxonomic analysis tools for this purpose, with MAPseq and QIIME 2 being two recently released alternatives. However, no independent and direct comparison between these four main tools has been performed. Here, we compared the default classifiers of MAPseq, mothur, QIIME, and QIIME 2 using synthetic simulated datasets comprised of some of the most abundant genera found in the human gut, ocean and soil environments. We evaluate their accuracy when paired with both different reference databases and variable sub-regions of the 16S rRNA gene.

Findings: We show that QIIME 2 provided the best recall and F-scores at genus and family levels, together with the lowest distance estimates between the observed and simulated samples. However, MAPseq showed the highest precision, with miscall rates consistently below 2%. Notably, QIIME 2 was the most computationally expensive tool, with CPU time and memory usage almost two and 30 times higher than MAPseq, respectively. Using the SILVA database generally yielded a higher recall than using Greengenes, while assignment results of different 16S rRNA variable sub-regions varied up to 40% between samples analysed with the same pipeline.

Conclusions: Our results support the use of either QIIME 2 or MAPseq for optimal 16S rRNA gene profiling, and we suggest that the choice between the two should be based on the level of recall, precision and/or computational performance required.

Findings

Background

Genome sequencing has provided an unprecedented view of the microbial diversity of ecosystems from wide-ranging environments. For example, the commensal flora of the human gut has been extensively explored for potential associations with the onset of many human diseases [1–3]. Similarly, the rich microbial diversity of environments such as soil and oceans have been studied in depth, yielding important ecological inferences [4–6]. There are now a substantial number of such microbial community datasets deposited in sequence archives (for example, the European Nucleotide Archive currently holds over 600 000 environmental samples [7]) and the rate of deposition is increasing. Drawing relevant biological correlations from this vast amount of data requires accurate and reliable tools and methods.

One of the crucial steps in almost all microbiome-based analyses is inference of community composition through taxonomic classification. For a few decades now [8], the common approach for taxonomic assignment of microbial species has been the classification of ribosomal RNA (rRNA) sequences. Currently, the most widely used tools for this purpose are the mothur [9] and "Quantitative Insights Into Microbial Ecology" (QIIME) software packages [10]. These correspond to large toolsets that are able to process, classify and perform downstream analyses on individual genetic markers (e.g. the 16S rRNA gene, conserved across the prokaryotic domains). For taxonomic classification, each tool compares a set of queried sequences against a defined reference database, such as Greengenes [11], NCBI [12], RDP [13] or SILVA [14], assigning the most likely taxonomic lineages. Ultimately, the success of these analyses is not only dependent on the breadth and diversity of annotated sequences available in public repositories, but also on the accuracy of the

classification algorithms used by each of the tools. By default, QIIME makes use of the UCLUST clustering method [15] to assign biological sequences to a reference database, while mothur reimplements the naïve Bayesian RDP classifier, developed by Wang, *et al.* [16]. Two other tools — MAPseq [17] and QIIME 2 (https://qiime2.org/) — have recently been released, the latter of which has officially replaced QIIME as of January, 2018. QIIME 2 also makes use of a naïve Bayes classifier [18], and MAPseq is a *k-mer* search approach that outputs confidence estimates at different taxonomic ranks.

A community-driven initiative known as the "Critical Assessment of Metagenome Interpretation" (CAMI) benchmarked a range of software tools for the analysis of shotgun metagenomic datasets [19]. In regard to amplicon-based approaches, previous studies have mainly evaluated the classification methods of QIIME and mothur, highlighting some of their advantages and pitfalls [20–22]. The recent publication of QIIME 2 also included the assessment of a number of different commonly used classifiers and marker gene regions [18]. However, until now, no independent study has compared the accuracy of MAPseq, mothur, QIIME and QIIME 2 whilst also taking into account potential differences arising from the use of distinct reference databases. Furthermore, for genotyping the 16S rRNA gene there is also much debate within the scientific community on the most informative variable sub-region to target [23]. Strong arguments have been made towards sequencing specific or combined sub-regions, such as the V4 [24] and V3-V4 [25], while difficulty in amplifying bacterial species, such as those from the *Actinobacteria* group, has prompted the development of more specialized primers [26,27]. The impact of variable region choice on the taxonomic classification performance of different tools or databases is therefore also important to assess.

The use of mock communities in microbiome studies has revealed that different experimental conditions and methods dramatically affect the quality of the results [28–31]. In contrast, *in silico* benchmarking approaches provide an agnostic view on the efficiency of the computational pipelines — independently of experimental variation and technical biases — but may require further validation in real-world datasets. Hence, for a holistic assessment of the validity of different methodological strategies, using both mock communities and *in silico* simulations is essential to understand the biases and limitations present at each stage of analysis.

In this work we have leveraged a set of simulated 16S rRNA gene sequences representative of genera commonly found in the human gut, ocean and soil environments, to evaluate the accuracy of the default taxonomic classifiers of MAPseq, mothur, QIIME and QIIME 2. We tested these methods with different reference databases, and according to some of the most commonly targeted sub-regions of the 16S rRNA gene. Our results showed that, regardless of the database used, QIIME 2 outperformed all other tools in terms of overall recall at both genus and family levels, as well as in distance estimations between the observed and predicted samples. Considerable performance differences were observed between using distinct 16S rRNA gene sub-regions, while limited software-dependent variation was seen between different reference databases. We believe this work will help inform microbial ecologists about important decisions to take when designing new 16S rRNA-based community studies.

Composition of the simulated datasets

The microbiota colonizing the human gut, ocean and soil environments are some of the most frequently studied microbial communities. Hence, to provide data with direct practical

applications, we focused on simulating datasets containing a diverse set of genera commonly found in these three ecosystems (Additional file: Fig. S1). Representative genera were selected after identifying the 80 most abundant genera across publicly available metagenomes from human gut, ocean and soil [7]. Then, for each biome, four different communities were generated with two levels of diversity: samples A100 and B100 with a random set of 100 species belonging to these genera; and A500 and B500 with 500 species. Final datasets comprised a total of 66, 66 and 76 different genera from the human gut, oceanic and soil environments, respectively. For the purpose of this benchmarking, we simulated the datasets with a similar relative abundance per genus to avoid introducing any taxon-specific biases (Additional file: Fig. S1).

To simulate a realistic scenario, where variation can arbitrarily occur and sequences may not have an exact representative in public databases, we randomly mutated 2% of the positions of each 16S rRNA sequence retrieved after extracting each sub-region using commonly used primer sequences [25,26,32–34] (Additional file: Table S1). Notably, the percentage of sequences retrieved from the Greengenes, NCBI, RDP and SILVA databases matching the primers selected for V1-V2 was dramatically lower (30.3%) than that of V3-V4 (90%), V4 (90.9%) and V4-V5 (87.8%) (Additional file: Fig. S2). The 16S rRNA V1 sub-region had been previously found to be truncated in a substantial number of reference sequences [23]. Our results confirm this observation and again raise caution at the use of the 16S V1-V2 rRNA primer sequences for complex and diverse samples, due to the reduced number of reference sequences available. Interestingly, the relative number of sequences retrieved from RDP was lower than that of the remaining databases (Additional file: Fig. S2), likely suggesting an overrepresentation of more divergent taxa that did not meet the mismatch threshold used in our *in silico* PCR.

Taxonomic assignment

Microbiome studies frequently strive to associate microbial diversity signatures with a phenotype of interest. However, focusing solely on high-level taxonomic ranks can severely underestimate the degree of variation observed between sample groups. To circumvent this, highly discriminative approaches are needed to be able to pinpoint the most significant taxa warranting further validation. For assessing the performance of MAPseq, mothur, QIIME and QIIME 2 with different reference databases (Additional file: Fig. S3), we limited our analyses to classification at the lineage level instead of operational taxonomic units (OTUs), as it allows a more consistent and easier interpretation of the results. Species assignment of every queried sequence would be the desired outcome, but as was previously shown [20], the limited resolution of the 16S rRNA locus precludes an accurate classification at this level. Furthermore, there is significant inconsistency in species nomenclature across all reference databases (e.g. RDP does not report taxon names below genus). In this work, we calculated the degree of recall and precision at the genus and family ranks, as in our opinion they provide the best compromise between classification accuracy and resolution.

By comparing the level of recall across all software tools, we found that QIIME 2 recovered the largest proportion of sequences from the expected genera (Table 1, Fig. 1 and Additional files: Tables S2, S3 and S4). Combined with the SILVA database, this resulted in the highest recall (sensitivity) for human gut (67.0%) and soil samples (68.3%), while the Greengenes database outperformed in the case of the oceanic microbiome (79.5%). In fact, all tools except QIIME saw a decrease in recall when using SILVA specifically for the classification of the oceanic dataset. Globally, however, SILVA most frequently provided a better genus recall than Greengenes (five out of nine comparisons across MAPseq, QIIME and QIIME 2,

Fig. 1). In terms of correctly identified taxa, MAPseq in conjunction with SILVA detected the greatest number of expected genera in all three biomes (Fig. 1). At the family level, all tools presented a substantially higher recall (Table 1), with QIIME 2 reaching 94.3% in the human gut sample, 96.2% with the ocean set and 91.7% with the soil sample (Additional files: Tables S2, S3 and S4).

Although the level of recall is a crucial metric in choosing the most appropriate taxonomic classification pipeline, it is equally important to ensure a low frequency of false-positive assignments. We evaluated the degree of precision (specificity) by the percentage of sequences assigned to the wrong taxon (Additional files: Tables S5, S6, S7) out of all the detected taxa. Accuracy was high for all the tools, with precision estimates of at least 84% across all analysis pipelines (Fig. 2A). In terms of total number of sequences, this translated to less than 10% of the reads misassigned at the genus level (Additional files: Tables S2, S3, S4 and Fig. S4). MAPseq with the SILVA database consistently outperformed all other tools, with a precision above 96% for the three tested biomes (Fig. 2A), equating to less than 2% of miscalled sequences.

To combine both recall and precision into a single metric, we calculated the F-score for all taxonomic assignments (Fig. 2A and and Fig. S5). At both genus and family levels, we found that QIIME 2 had the highest score across the samples representative of the three different biomes, with the SILVA database coming out on top for the human gut (genus: 78.9%, family: 96.8%, Additional file: Table S2) and soil (genus: 78.5%, family: 94.3%, Additional file: Table S4) environments in particular, but the Greengenes database performing better with the oceanic dataset (genus: 87.4%, family: 97.4%, Additional file: Table S3). After fractioning the data according to different sub-regions of the 16S rRNA gene, we then

repeated the same analysis (Fig. 2B). This revealed that the performance of each tool varied up to 40% depending on the 16S rRNA sub-region targeted. Notably, the V1-V2 or V3-V4 sub-regions performed the best across most of the pipelines (Fig. 2B). In our study, each synthetic species had a genetically close full-length 16S rRNA sequence represented in the databases, so our tests were probably not significantly affected by the reduced number of V1-V2 reference sequences available.

The ongoing surge in genome sequencing is producing thousands of novel sequences each year. Therefore, efficient tools that can scale up to provide analysis of tens of thousands of samples is increasingly important. With this in mind, we compared the computational performance of MAPseq, mothur, QIIME and QIIME 2 throughout the whole classification pipeline of our simulated datasets. We analysed average memory usage and CPU time across the three biomes for the processing and assignment of 3 million quality-filtered sequences against the SILVA 128 database (Fig. 3). MAPseq was the most memory-efficient tool, with mothur, QIIME and QIIME 2 requiring over 72, 15 and 27 times more memory resources, respectively (Fig. 3A). CPU time of QIIME 2 was the highest, close to twice that of MAPseq, and almost 100 times longer than QIIME, which was the fastest (Fig. 3B). Of note is that each pipeline has its own processing procedure; both the mothur and QIIME 2 pipelines included a de-replication step of the query sequences prior to taxonomic assignment, which substantially reduced the number of sequences used for classification.

Relative quantification and beta diversity

One of the main aspects of any microbiome-based analysis is the assessment of the differential abundance and beta diversity across a set of sample groups. In this respect, accurate estimation of the relative abundance of each taxon is essential to find statistically

significant patterns. To assess how accurately each tool was able to predict taxa relative abundances in each sample, we calculated dissimilarity scores (DS) for each genus present in the simulated dataset (Fig. 4). Interestingly, QIIME 2 showed the most accurate prediction in relation to the true genera composition, with an average DS of 0.33 when used in conjunction with the SILVA database (Table 1). In terms of the reference database used, analyses carried out with SILVA yielded more accurate predictions than with the Greengenes database (Additional files: Tables S2, S3 and S4). Substantial differences in accuracy were observed across different genera, with sequences from the *Paraprevotella* genus — frequently present in human gut samples — more accurately predicted, in contrast to those from *Coprobacter*, *Hyphomicrobium* and *Thalassobacter*, which had the worst results (Fig. 4). These genera might either be underrepresented in the reference databases or have a high degree of conservation with other closely related taxa, making accurate taxonomic assignments more challenging.

For a global assessment of the beta diversity across samples, we performed a principal coordinates analysis (PCoA) and calculated both Bray-Curtis and Jaccard distances between the observed and expected results. Both distance methods represent complementary approaches, as the Bray-Curtis metric corresponds to a quantitative evaluation of the dissimilarity across samples, whereas the Jaccard index is a qualitative measure of community similarity. We found that samples analysed with QIIME 2 were the closest (i.e. had the lowest distance estimate) to the true simulated datasets, with minor differences between the use of SILVA or Greengenes with both the Bray-Curtis and Jaccard methods (Table 1; Fig. 5).

Discussion

With the number of tools, databases and options available for taxonomic classification of marker sequences, it can be a daunting task to decide the optimal approach for analysis of a specific dataset. In this work, we have strived to help guide this decision-making process by independently assessing the performance of the most commonly used taxonomic assignment strategies with simulated samples comprised of genera found in frequently sampled environments.

Overall, we show that all tools we tested performed moderately well, with high precision and modest-to-high recall rates at the genus level. QIIME 2 presents significant improvements over the other tools, particularly over the preceding version of QIIME, in regard to detection sensitivity at both family and genus level. It should be emphasized that as of January, 2018 QIIME has been replaced by QIIME 2 and the former tool is no longer supported by the developers. The superiority of QIIME 2 also held true for the prediction of sample composition, as beta diversity estimates between the analysed and simulated communities were the closest using this method. Therefore, these data support the use of QIIME 2 to obtain the largest proportion of classified sequences at the most accurate relative abundances. Nevertheless, the results also showed MAPseq to be a more conservative and precise approach, meaning that fewer genera were misassigned. In addition, this tool showed considerably better computational performance than QIIME 2, requiring approximately 30 times less memory and almost half the CPU time to process the same dataset (even though QIIME 2 classifies substantially fewer query sequences due to a prior de-replication step). These results show that MAPseq provides a credible option if precision and computational performance or scale are a priority.

Selecting a single best software package is not a straightforward affair, and we expect that further differences in performance will be observed with different real-world datasets.

Additionally, mothur and QIIME 2 also provide the option of using multiple taxonomic classifiers, so improvements in overall recall and/or precision metrics might be possible with the other available methods, combined with further parameter optimization. We should also stress that, aside from the software packages we tested, other web-based tools such as BioMaS [22] are also available. However, they are usually restricted to the use of specific reference databases, making individual customizations and accurate comparisons more challenging.

In addition to choosing the right tool, combining that with the appropriate reference database is equally important to ensure the best classification performance. Greengenes and SILVA have been the most widely-used and readily supported databases. Generally, the SILVA 128 database performed better than Greengenes 13_8 in terms of recall at both genus and family levels, as well as in predicting the true taxa composition of the simulated communities. Conversely, there was an almost universal decrease in its performance in the detection of ocean-specific taxa, so special care should be taken in the analysis of datasets sampled from this particular environment. Nonetheless, there are additional advantages to the use of SILVA: it is more frequently updated (Greengenes was last updated in May 2013); it includes rRNA sequences of eukaryotic organisms in addition to archaea and bacterial species; and has been shown to be more easily comparable and mapped to other taxonomies such as the NCBI [35]. In the case of MAPseq and mothur, the NCBI and RDP databases also performed well, with higher recall but slightly lower precision scores compared to SILVA. Therefore, the SILVA, RDP or NCBI databases are all appropriate choices for a comprehensive and accurate taxonomic analysis.

The choice of primer sequences for taxonomic profiling of the 16S rRNA gene has been a matter of frequent debate. In common with previously reported observations [27], we show

that targeting different sub-regions can considerably influence the taxonomic assignment performance (by up to 40% in our analyses). Overall, the V1-V2 and V3-V4 sub-regions performed the best across most of the tools. However, the V1-V2 primers did not match almost 70% of the sequences across the four reference databases, so we discourage its use for classification of complex community samples. As our simulated datasets were generated from close representatives containing full-length 16S rRNA genes, it is reasonable to assume that our analysis of the V1-V2 sub-region was not significantly hampered by this reduced number of reference sequences. Kozich *et al*. [24] have argued in favour of standardizing the use of the V4 sub-region for Illumina MiSeq sequencing, as it allows complete overlap of paired-end sequences, mitigating sequence errors introduced during PCR amplification or sequencing. Phylogenetic studies have also showed that the V4 sub-region is the closest representative of the phylogenetic signal of the whole 16S rRNA locus [23]. Here, we analysed the performance of some of the most commonly used sub-regions under a purely computational perspective, and conclude that amplification of the V3-V4 sub-region is most frequently the best option for a reliable taxonomic inference.

In summary, we have identified the major benefits and drawbacks of the most recent and popular taxonomic classification methods. Importantly, we show that the choice of software, database and sub-region significantly affects the quality of the classification results. Given the impact of each of these variables, it is imperative to strive for consistency in the analysis of samples not only within individual studies, but across different projects as well. Services like the EBI Metagenomics [7] and MG-RAST [36] help provide a basis for standardization, but additional factors relating to the experimental design are up to individual users to decide. Some attempts have been made to find recommended best practices for 16S microbiome studies among the myriad of options and issues that can arise at each analysis stage [37]. We

believe our work presented here further complements these efforts by helping the microbiome research community make more informed decisions about the most appropriate methodological approach to take in their own analysis pipeline.

Methods

Generating simulated datasets

Twelve sets of synthetic communities were generated for evaluating the accuracy of the taxonomic assignment pipelines: four each for human gut, ocean and soil environments. First, the 80 most abundant genera across publicly deposited samples from these biomes were retrieved using the EBI Metagenomics API (https://www.ebi.ac.uk/metagenomics/api/) [7]. This list was then used to randomly select either 100 (datasets A100 and B100) or 500 species (datasets A500 and B500) belonging to these genera, allowing a maximum of 20 and 50 species per genus, respectively. 16S rRNA gene sequences were obtained from the European Nucleotide Archive (ENA), and an *in silico* PCR was carried out with a python script (https://github.com/simonrharris/in_silico_pcr) to extract commonly used regions for 16S rRNA profiling [25,26,32–34] (Additional file: Table S1), allowing a maximum of three mismatches per primer sequence. Subsequently, 2% of the positions in each variable region were randomly mutated to create nucleotide diversity, using a custom python script (https://github.com/Finn-Lab/Tax-Benchmarking). Sequencing reads were simulated from these amplicon sequences in duplicate using the MiSeq v3 error profile with ART (ART,

RRID:SCR_006538) [38], generating ~ 10 000 and ~ 200 000 paired-end reads of 250 bp per region to have samples representing both low and high levels of sequencing depth.

Sequence classification

Initial pre-processing and quality control was performed following the mothur standard operating procedure (SOP) [24], accessed on November 2017. Briefly, the *make.contigs* command was used to align, filter and merge the paired-end reads into contigs. Subsequently, we used the *screen.seqs* command to filter out any sequences with ambiguous base calls. This final set of quality controlled sequences was then assigned into taxonomic lineages with MAPseq v1.2.2 [17], mothur v1.39.5 (mothur , RRID:SCR_011947)[9], QIIME 1.9.1 (QIIME, RRID:SCR_008249)[10], and QIIME 2 v2017.11 (https://qiime2.org/). For each software, we evaluated the settings and databases most frequently used and recommended for optimal taxonomic classification (Additional file: Fig. S3). With MAPseq, we tested the default NCBI database (mapref 2.2), as well as Greengenes 13_8 and the SILVA 128 database re-mapped to an eight-level taxonomy (available in ftp://ftp.ebi.ac.uk/pub/databases/metagenomics/mapseq_silva128). Each set of reference sequences was analysed following the internal clustering by MAPseq. Options -*tophits 80* and -*topotus 40* were used in combination with the -*outfmt simple* option. For QIIME 1.9.1, the *pick_closed_reference_otus.py* script was used with the default Greengenes database (13_8) and with SILVA 128, both clustered at 97% identity. Taxonomic assignment with mothur was carried out according to the MiSeq SOP [24], excluding the chimera detection and removal steps, using the available pre-formatted SILVA 128 database for alignment and either the RDP version 16 or SILVA 128 for sequence classification. We did not use the Greengenes alignment database as per the mothur SOP [39]. Lastly, for QIIME 2 we first dereplicated the query sequences using the *vsearch dereplicate-sequences* function and then

assigned them to the Greengenes (13_8) or SILVA 128 (99% identity clusters) databases using the *feature-classifier classify-sklearn* function [18].

Analysis and visualization

TSV and BIOM files were generated from the MAPseq and QIIME 2 outputs and combined with the output BIOM files created by QIIME and mothur (*make.biom* command). Taxonomy names obtained from each individual reference database were normalized so that each genus and family would be assigned to the same lineage. Results were visualized and analysed with the phyloseq (phyloseq, RRID:SCR_013080)[40] and vegan R packages (vegan, RRID:SCR_011950). The recall rate (sensitivity) for each tool and database was estimated as the percentage of sequences assigned to the expected taxa for each biome, while precision (specificity) was calculated as the fraction of sequences from these predicted taxa out of all those from the taxa observed. Finally, the F-score was calculated as follows:

$$\text{F–score} = 2 \times \frac{\text{precision} \times \text{recall}}{\text{precision} + \text{recall}}$$

Distance estimates were calculated with either the Bray-Curtis or Jaccard dissimilarity indices after grouping the taxonomic lineages at the genus level. Principal coordinate analysis (PCoA) were performed with the Bray-Curtis distance method. Dissimilarity scores (DS) on the relative abundance (rel.ab) of each expected genus were calculated as:

$$\text{DS} = \frac{|\text{rel.ab.(Observed)} - \text{rel.ab.(Expected)}|}{\text{rel.ab.(Expected)}}$$

Memory usage and CPU time was estimated as the total amount required for the processing and assignment of all combined sequences against the SILVA 128 database (clustered at 99%), following the protocols described above.

Availability of supporting source code and requirements

Project name: Taxonomy benchmarking

Project home page: https://github.com/Finn-Lab/Tax-Benchmarking

Operating system: Platform independent

Programming languages: Python 2.7, R 3.4.1

Other requirements: BioPython module, R libraries (ggplot2, phyloseq, vegan, scales, grid, ape, RColorBrewer, data.table)

License: MIT

Availability of supporting data

The datasets supporting the conclusions of this article are available in the GigaDB repository [41].

Declarations

List of abbreviations

CPU: Central Processing Unit

DS: Dissimilarity score

ENA: European Nucleotide Archive

GG: Greengenes

OTU: Operational Taxonomic Unit

PCoA: Principal coordinates analysis

RDP: Ribosomal Database Project

rRNA : ribosomal rRNA

Ethics approval and consent to participate

Not applicable

Consent for publication

Not applicable

Competing interests

The authors declare that they have no competing interests

Funding

European Molecular Biology Laboratory (EMBL); European Commission within the Research Infrastructures Programme of Horizon 2020 [676559] (ELIXIR-EXCELERATE)

Authors' contributions

AA, ALM, AT and RDF performed the analyses. AA, ALM and RDF conceived the study and wrote the manuscript. All authors have read and approved the final manuscript.

Acknowledgements

We thank João Matias Rodrigues for providing useful comments on the utility of MAPseq in relation to the benchmarking.

Figure legends

Figure 1. Level of recall at the genus level, represented as taxa relative abundances, obtained with each analysis pipeline for the three different biomes (human gut, ocean and soil). The number of genera correctly identified by each pipeline is indicated above the graph.

Figure 2. (A) Recall, precision and F-score estimates at the genus level for each tool and database tested. (B) F-scores calculated for some of the most commonly tested sub-regions of the 16S rRNA gene: V1-V2, V3-V4, V4 and V4-V5.

Figure 3. Computational cost of each taxonomy assignment tool, estimated as the total memory usage (A) and CPU time (B) required for the processing and classification of ~ 3 million sequences against the SILVA 128 database. Error bars denote standard deviation across the three biomes tested (human gut, ocean and soil).

Figure 4. Dissimilarity scores (DS) calculated for each genus included in the simulated datasets. Lower (brighter) values indicate a closer prediction to the true composition of the original sample. The black outline indicates the overall best scoring analysis pipeline for each environment. Taxa are ordered by decreasing abundance from left to right, based on their composition in the simulated sample.

Figure 5. Principal coordinates analysis (PCoA) between all samples analysed in relation to the true, expected dataset, using the Bray-Curtis distance method.

Figure S1. Composition of the synthetic communities per selected environment. Samples A100 and B100 are randomly generated sets of 100 species, while A500 and B500 were simulated from 500 different species.

Figure S2. Percentage of sequences retrieved from the Greengenes, NCBI, RDP and SILVA databases with an *in silico* PCR targeting different 16S rRNA gene sub-regions.

Figure S3. Tools and databases benchmarked in our study. We tested at least two databases per software tool. The reference databases used were either readily supported by the specific tool and/or recommended by their developers. SILVA was compared across all tools; MAPseq was specifically assessed with the NCBI database, its default reference; mothur was not paired with Greengenes due to its poor-quality alignment [39] and was analysed with RDP instead.

Figure S4. Number of genera misassigned in each analysis pipeline and their overall relative abundance. Names and abundance values of each misclassified taxon are included as additional files (Additional files: Tables S5, S6 and S7).

Figure S5. Recall, precision and F-score estimates at the family level for each tool and database tested.

References

1. Forbes JD, Van Domselaar G, Bernstein CN. The gut microbiota in immune-mediated inflammatory diseases. Front. Microbiol. 2016;7:1081.

2. Duvallet C, Gibbons SM, Gurry T, Irizarry RA, Alm EJ. Meta-analysis of gut microbiome studies identifies disease-specific and shared responses. Nat. Commun. 2017;8:1784.

3. Turnbaugh PJ, Ley RE, Mahowald MA, Magrini V, Mardis ER, Gordon JI. An obesity-associated gut microbiome with increased capacity for energy harvest. Nature. 2006;444:1027–31.

4. Thompson LR, Sanders JG, McDonald D, Amir A, Ladau J, Locey KJ, et al. A communal catalogue reveals Earth's multiscale microbial diversity. Nature. 2017;551:457–63.

5. Yilmaz P, Yarza P, Rapp JZ, Glöckner FO. Expanding the world of marine bacterial and archaeal clades. Front. Microbiol. 2016;6:1524.

6. Fierer N. Embracing the unknown: disentangling the complexities of the soil microbiome. Nat. Rev. Microbiol. 2017;15:579–90.

7. Mitchell AL, Scheremetjew M, Denise H, Potter S, Tarkowska A, Qureshi M, et al. EBI Metagenomics in 2017: enriching the analysis of microbial communities, from sequence reads to assemblies. Nucleic Acids Res. 2017;46:D726–35.

8. Pace NR, Stahl DA, Lane DJ, Olsen GJ. The analysis of natural microbial populations by ribosomal RNA sequences. Adv. Microb. Ecol. 1986. p. 1–55.

9. Schloss PD, Westcott SL, Ryabin T, Hall JR, Hartmann M, Hollister EB, et al. Introducing mothur: open-source, platform-independent, community-supported software for describing and comparing microbial communities. Appl. Environ. Microbiol. 2009;75:7537–41.

10. Caporaso JG, Kuczynski J, Stombaugh J, Bittinger K, Bushman FD, Costello EK, et al. QIIME allows analysis of high-throughput community sequencing data. Nat. Methods. 2010;7:335–6.

11. McDonald D, Price MN, Goodrich J, Nawrocki EP, DeSantis TZ, Probst A, et al. An improved Greengenes taxonomy with explicit ranks for ecological and evolutionary analyses of bacteria and archaea. ISME J. 2012;6:610–618.

12. Federhen S. The NCBI Taxonomy database. Nucleic Acids Res. 2012;40:D136–43.

13. Cole JR, Wang Q, Fish JA, Chai B, McGarrell DM, Sun Y, et al. Ribosomal Database Project: data and tools for high throughput rRNA analysis. Nucleic Acids Res. 2014;42:D633–42.

14. Yilmaz P, Parfrey LW, Yarza P, Gerken J, Pruesse E, Quast C, et al. The SILVA and “All-species Living Tree Project (LTP)” taxonomic frameworks. Nucleic Acids Res. 2014;42:D643–8.

15. Edgar RC. Search and clustering orders of magnitude faster than BLAST. Bioinformatics. 2010;26:2460–1.

16. Wang Q, Garrity GM, Tiedje JM, Cole JR. Naive Bayesian classifier for rapid assignment of rRNA sequences into the new bacterial taxonomy. Appl. Environ. Microbiol. 2007;73:5261–7.

17. Matias Rodrigues JF, Schmidt TSB, Tackmann J, von Mering C. MAPseq: highly efficient k-mer search with confidence estimates, for rRNA sequence analysis. Bioinformatics. 2017;33:3808–10.

18. Bokulich NA, Kaehler BD, Rideout JR, Dillon M, Bolyen E, Knight R, et al. Optimizing taxonomic classification of marker gene amplicon sequences. PeerJ (preprint). 2018;

19. Sczyrba A, Hofmann P, Belmann P, Koslicki D, Janssen S, Dröge J, et al. Critical Assessment of Metagenome Interpretation—a benchmark of metagenomics software. Nat. Methods. 2017;14:1063–71.

20. Golob JL, Margolis E, Hoffman NG, Fredricks DN. Evaluating the accuracy of amplicon-based microbiome computational pipelines on simulated human gut microbial communities.

BMC Bioinformatics. 2017;18:283.

21. Lindgreen S, Adair KL, Gardner PP. An evaluation of the accuracy and speed of metagenome analysis tools. Sci. Rep. 2015;6:19233.

22. Fosso B, Santamaria M, Marzano M, Alonso-Alemany D, Valiente G, Donvito G, et al. BioMaS: a modular pipeline for Bioinformatic analysis of Metagenomic AmpliconS. BMC Bioinformatics. 2015;16:203.

23. Yang B, Wang Y, Qian P-Y. Sensitivity and correlation of hypervariable regions in 16S rRNA genes in phylogenetic analysis. BMC Bioinformatics. 2016;17:135.

24. Kozich JJ, Westcott SL, Baxter NT, Highlander SK, Schloss PD. Development of a dual-index sequencing strategy and curation pipeline for analyzing amplicon sequence data on the MiSeq Illumina sequencing platform. Appl. Environ. Microbiol. 2013;79:5112–20.

25. Klindworth A, Pruesse E, Schweer T, Peplies J, Quast C, Horn M, et al. Evaluation of general 16S ribosomal RNA gene PCR primers for classical and next-generation sequencing-based diversity studies. Nucleic Acids Res. 2012;41:e1.

26. Walker AW, Martin JC, Scott P, Parkhill J, Flint HJ, Scott KP. 16S rRNA gene-based profiling of the human infant gut microbiota is strongly influenced by sample processing and PCR primer choice. Microbiome. 2015;3:26.

27. Comeau AM, Douglas GM, Langille MGI. Microbiome Helper: a custom and streamlined workflow for microbiome research. mSystems. 2017;2:e00127-16.

28. Brooks JP, Edwards DJ, Harwich MD, Rivera MC, Fettweis JM, Serrano MG, et al. The truth about metagenomics: quantifying and counteracting bias in 16S rRNA studies. BMC Microbiol. 2015;15:66.

29. Schloss PD, Gevers D, Westcott SL. Reducing the effects of PCR amplification and sequencing artifacts on 16S rRNA-based studies. PLOS One. 2011;6.

30. Quince C, Walker AW, Simpson JT, Loman NJ, Segata N. Shotgun metagenomics, from

sampling to analysis. Nat. Biotechnol. 2017;35:833–44.

31. Shakya M, Quince C, Campbell JH, Yang ZK, Schadt CW, Podar M. Comparative metagenomic and rRNA microbial diversity characterization using archaeal and bacterial synthetic communities. Environ. Microbiol. 2013;15:1882–99.

32. Mahmoud KK, McNeely D, Elwood C, Koval SF. Design and performance of a 16S rRNA-targeted oligonucleotide probe for detection of members of the genus Bdellovibrio by fluorescence in situ hybridization. Appl. Environ. Microbiol. 2007;73:7488–93.

33. Turner S, Pryer K, Miao V, Palmer J. Investigating deep phylogenetic relationships among cyanobacteria and plastids by small subunit rRNA sequence analysis. J. Eukaryot. Microbiol. 1999;46:327–38.

34. Schloss PD, Jenior ML, Koumpouras CC, Westcott SL, Highlander SK. Sequencing 16S rRNA gene fragments using the PacBio SMRT DNA sequencing system. PeerJ. 2016;4:e1869.

35. Balvočiute M, Huson DH. SILVA, RDP, Greengenes, NCBI and OTT — how do these taxonomies compare? BMC Genomics. 2017;18:114.

36. Keegan KP, Glass EM, Meyer F. MG-RAST, a metagenomics service for analysis of microbial community structure and function. Methods Mol. Biol. 2016. p. 207–33.

37. Pollock J, Glendinning L, Wisedchanwet T, Watson M. The madness of microbiome: attempting to find consensus “best practice” for 16S microbiome studies. Appl. Environ. Microbiol. 2018;84:e02627-17.

38. Huang W, Li L, Myers JR, Marth GT. ART: a next-generation sequencing read simulator. Bioinformatics. 2012;28:593–4.

39. Schloss P. mothur developer webpage. https://www.mothur.org/wiki/Greengenes-formatted_databases. Accessed: November 2017.

40. McMurdie PJ, Holmes S, Watson M. phyloseq: an R package for reproducible interactive

analysis and graphics of microbiome census Data. PLOS One. 2013;8:e61217.

41. Almeida, A; Mitchell, A, L; Tarkowska, A; Finn, R, D (2018): Supporting data for "Benchmarking taxonomic assignments based on 16S rRNA gene profiling of the microbiota from commonly sampled environments" GigaScience Database. http://dx.doi.org/10.5524/100448

Table 1. Global metrics averaged across the analyses of simulated samples from human gut, ocean and soil.

		Family		Genus					
**Software**	**Database**	**Recall**	**Miscalled**	**Recall**	**Miscalled**	**Sub-region[1]**	**Mean DS**	**Bray-Curtis**	**Jaccard**
MAPseq	Greengenes	88.3	2.4	58.9	2.4	V3-V4	0.434	0.282	0.440
MAPseq	NCBI	81.7	1.3	51.7	2.0	V3-V4	0.522	0.330	0.495
MAPseq	SILVA	67.2	**0.7**	46.5	**1.0**	V3-V4	0.482	0.373	0.540
mothur	RDP	85.4	3.2	50.5	5.0	V3-V4	0.419	0.356	0.523
mothur	SILVA	82.9	2.4	40.8	5.2	V3-V4	0.492	0.446	0.613
QIIME 2	Greengenes	93.2	1.6	**69.2**	3.4	V3-V4	0.367	**0.210**	**0.342**
QIIME 2	SILVA	**93.6**	1.9	69.0	4.3	V3-V4	**0.331**	0.211	0.348
QIIME	Greengenes	59.4	1.6	45.1	2.5	V4	0.585	0.394	0.564
QIIME	SILVA	66.4	2.1	57.5	6.5	V4	0.432	0.309	0.470

Values in bold denote the best score.

[1]Sub-region with the highest F-score, excluding V1-V2.

Figure 1

Click here to download Figure Figure_1.pdf

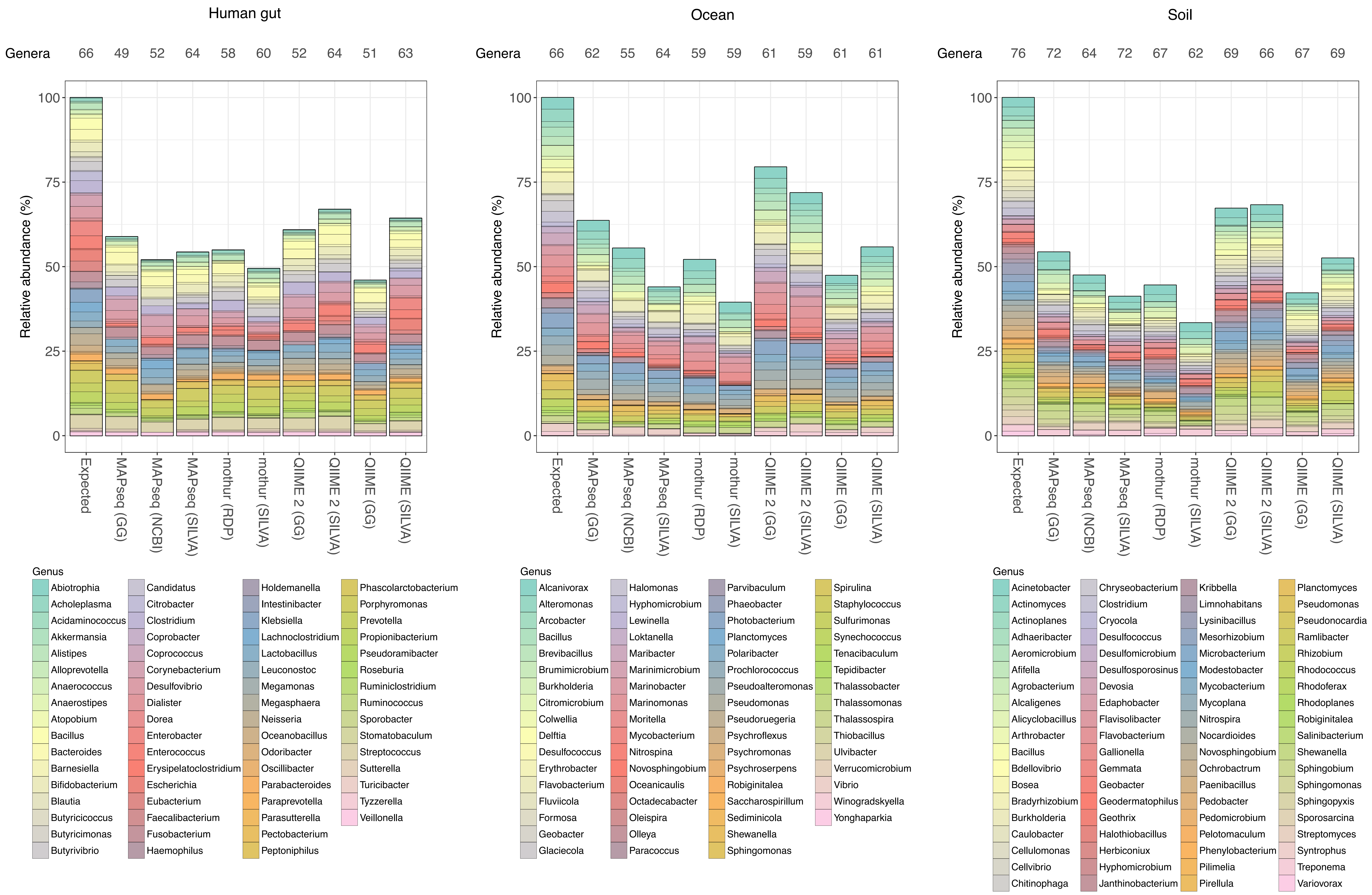

Figure 2

Click here to download Figure Figure_2.pdf

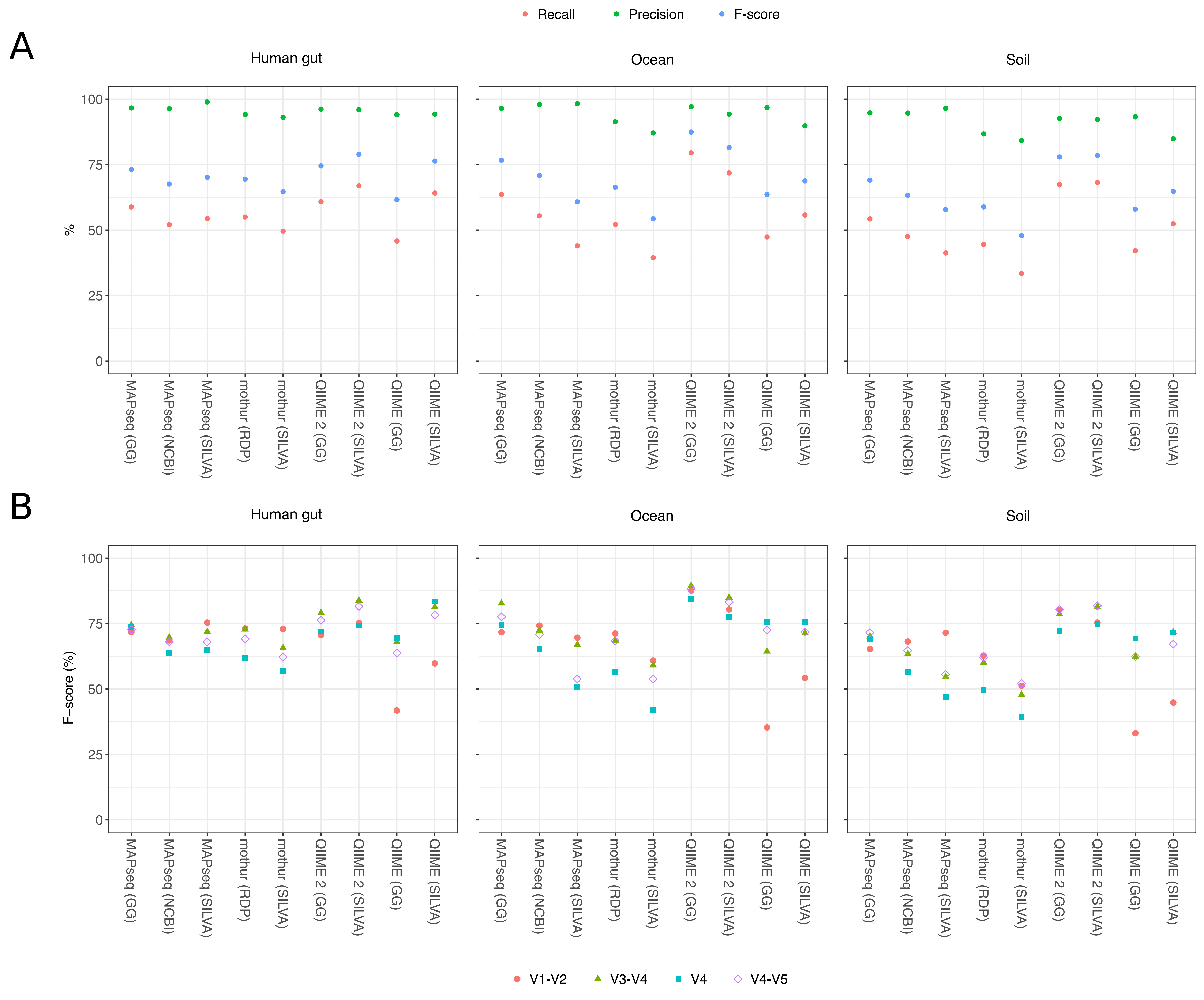

Figure 3 Click here to download Figure Figure_3.pdf

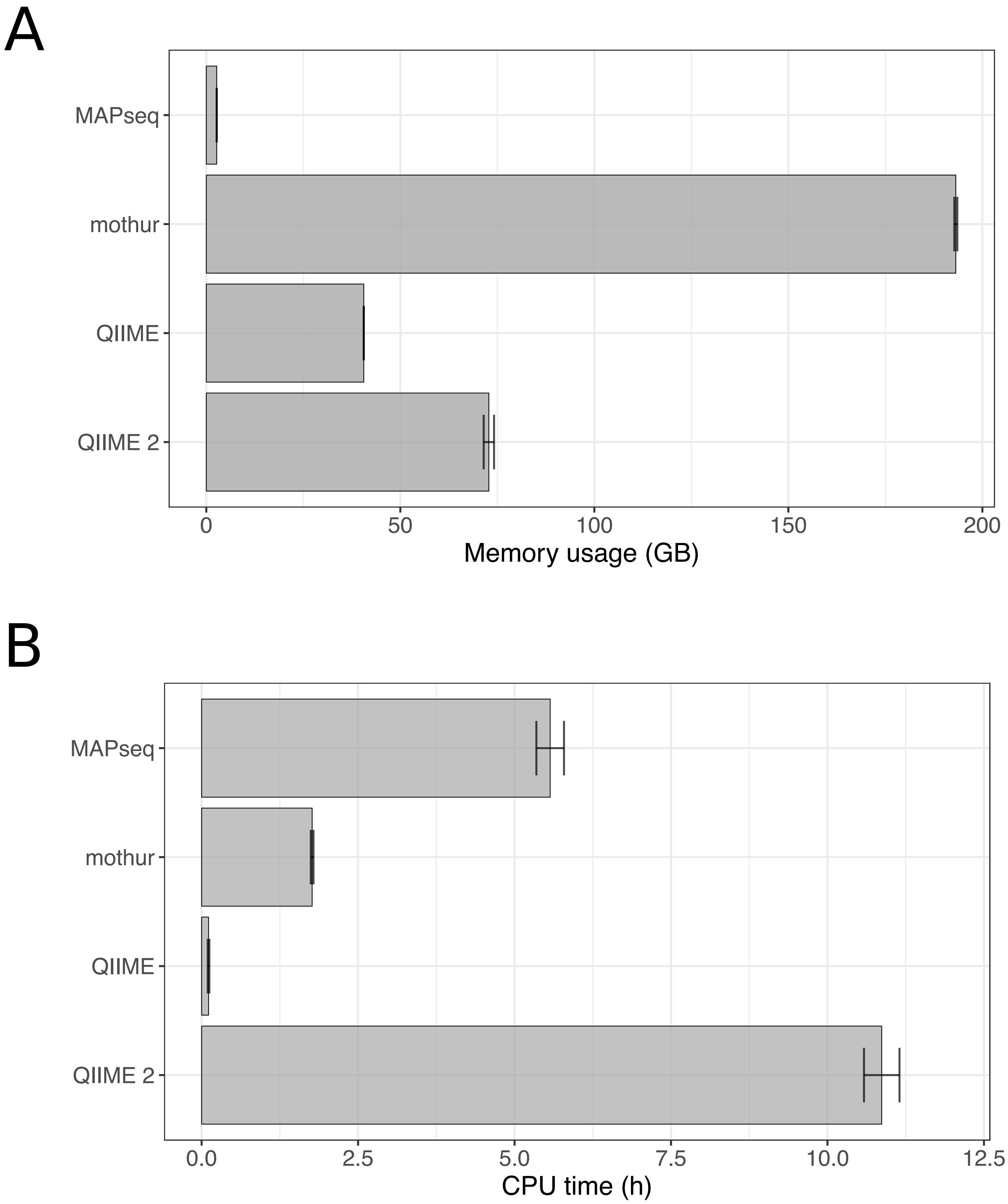

Figure 4

Click here to download Figure Figure_4.pdf

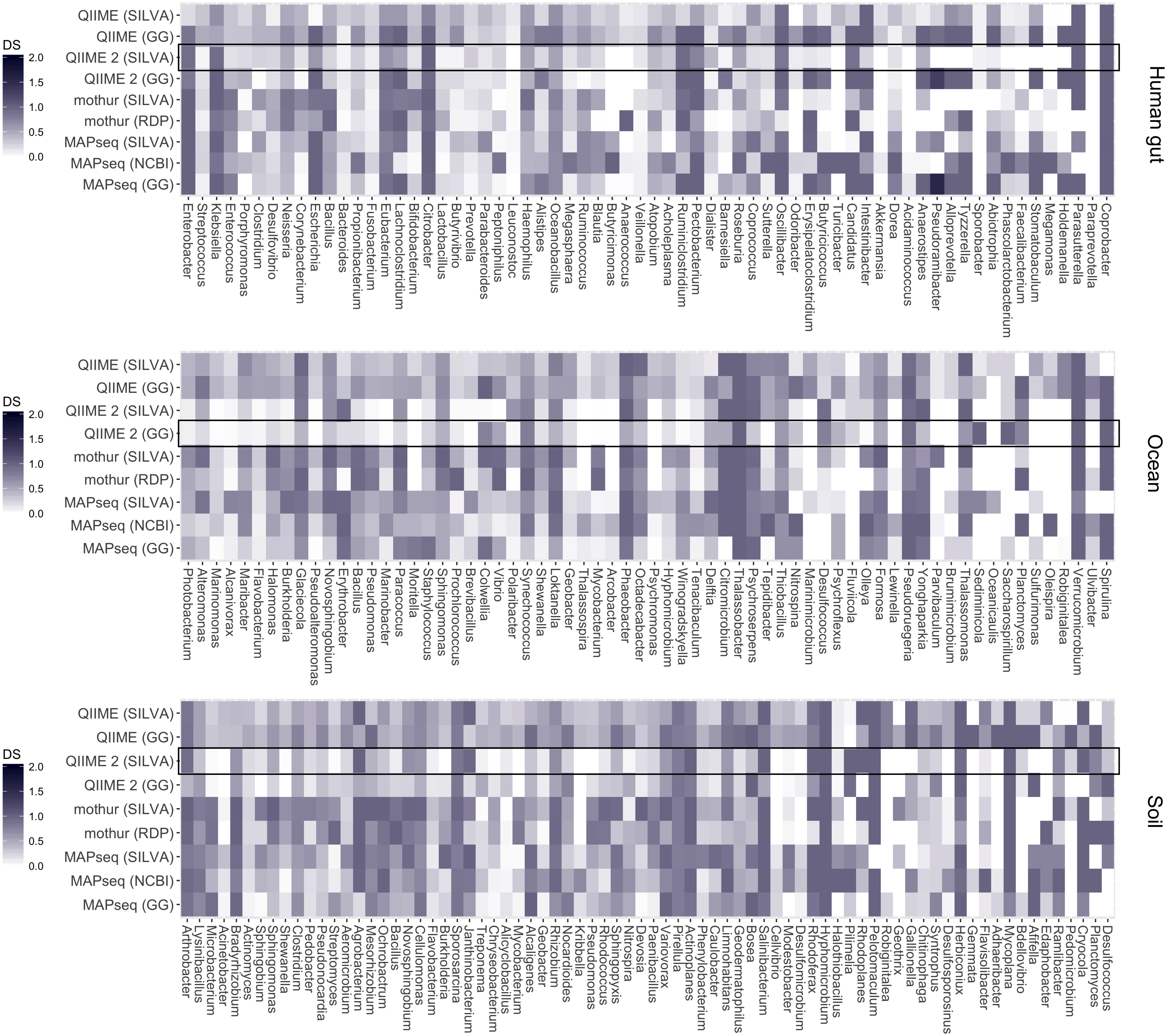

Figure 5

Click here to download Figure Figure_5.pdf

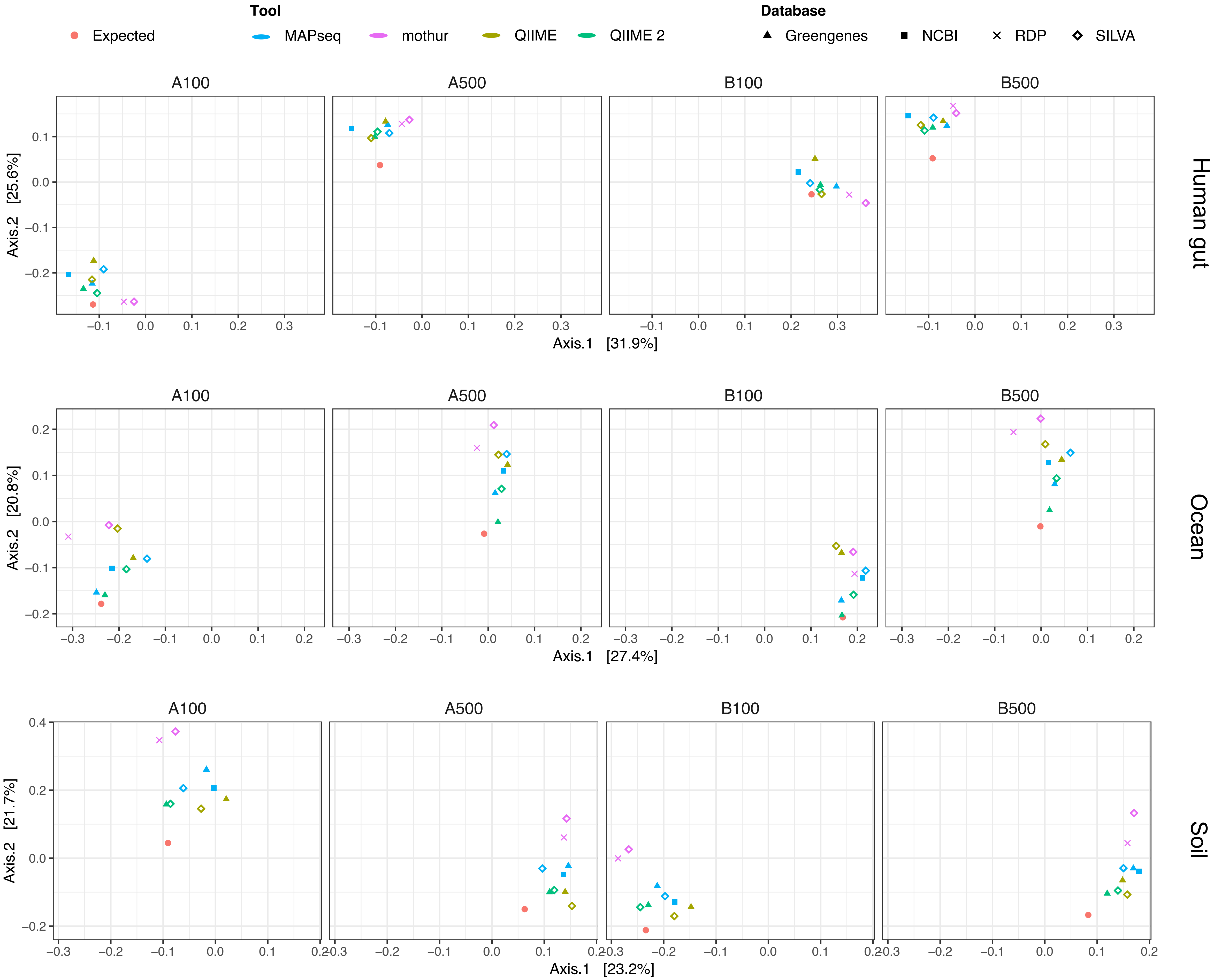

Table S1

Click here to access/download
Supplementary Material
Table_S1.xlsx

Table S2

Click here to access/download
Supplementary Material
Table_S2.xlsx

Table S3

Click here to access/download

Supplementary Material

Table_S3.xlsx

Table S4

Click here to access/download
Supplementary Material
Table_S4.xlsx

Table S5

Click here to access/download
Supplementary Material
Table_S5.xlsx

Table S6

Click here to access/download
Supplementary Material
Table_S6.xlsx

Table S7

Click here to access/download
Supplementary Material
Table_S7.xlsx

Figure S1

Click here to access/download
Supplementary Material
Figure_S1.pdf

Figure S2

Click here to access/download
Supplementary Material
Figure_S2.pdf

Figure S3

Click here to access/download
Supplementary Material
Figure_S3.pdf

Figure S4

Click here to access/download
Supplementary Material
Figure_S4.pdf

Figure S5

Click here to access/download
Supplementary Material
Figure_S5.pdf
