# Supplementary material for: Benchmarking taxonomic assignments based on 16S rRNA gene profiling of the microbiota from commonly sampled environments
Source: Gigascience. 2018 May 11;7(5):giy054. doi: 10.1093/gigascience/giy054 (PMC5967554; doi:10.1093/gigascience/giy054)
Supplement: Supplemental material [file giy054_supp.zip › Figure_S1.pdf]

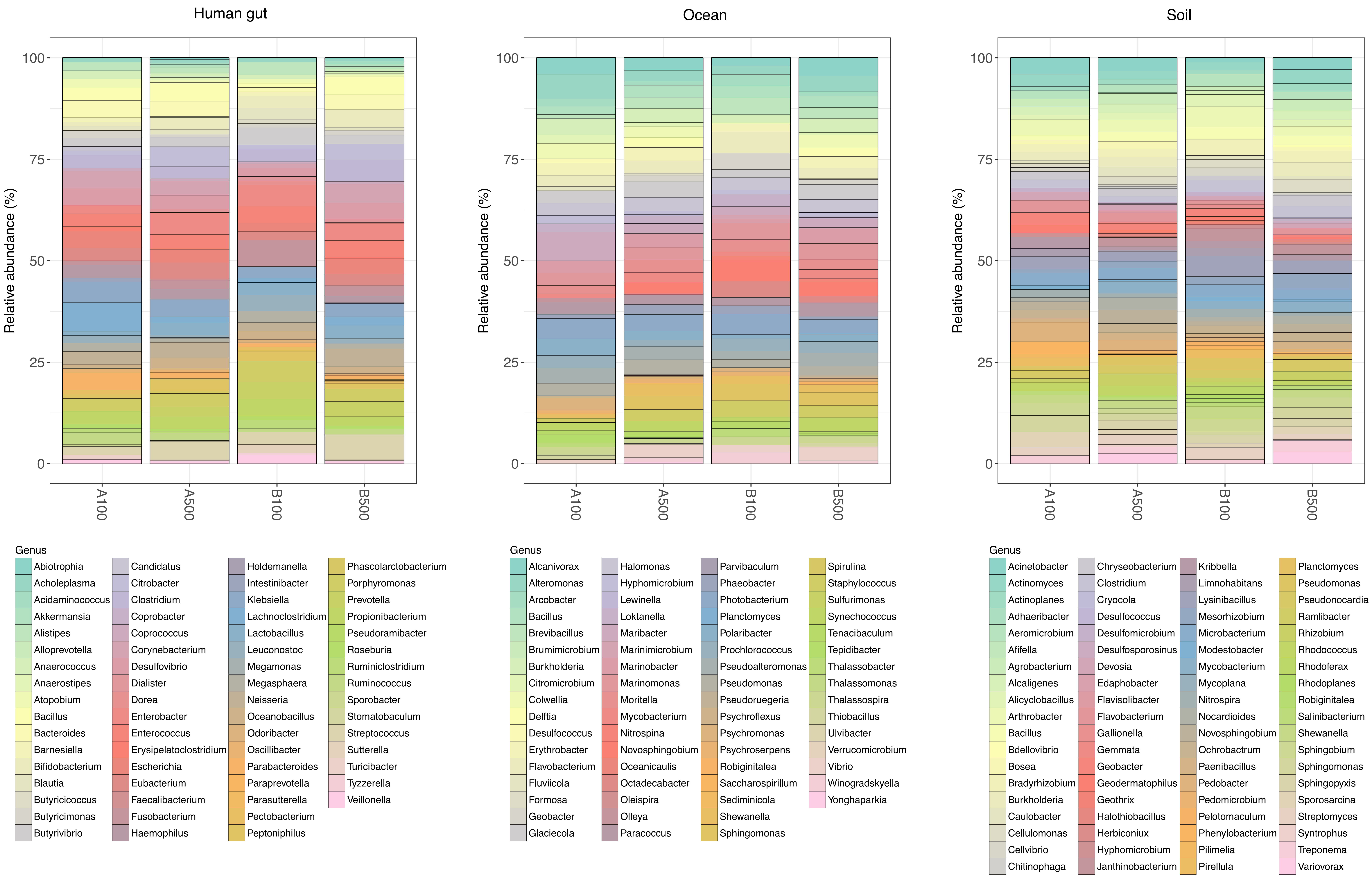
Human gut
Ocean
Soil
Relative abundance (%)
0
25
50
75
100
A100
A500
B100
B500
Genus
Abiotrophia
Acholeplasma
Acidaminococcus
Akkermansia
Alistipes
Alloprevotella
Anaerococcus
Anaerostipes
Atopobium
Bacillus
Bacteroides
Barnesiella
Bifidobacterium
Blautia
Butyricicoccus
Butyricimonas
Butyrivibrio
Candidatus
Citrobacter
Clostridium
Coprobacter
Coprococcus
Corynebacterium
Desulfovibrio
Dialister
Dorea
Enterobacter
Enterococcus
Erysipelatoclostridium
Escherichia
Eubacterium
Faecalibacterium
Fusobacterium
Haemophilus
Holdemanella
Intestinibacter
Klebsiella
Lachnoclostridium
Lactobacillus
Leuconostoc
Megamonas
Megasphaera
Neisseria
Oceanobacillus
Odoribacter
Oscillibacter
Parabacteroides
Paraprevotella
Parasutterella
Pectobacterium
Peptoniphilus
Phascolarctobacterium
Porphyromonas
Prevotella
Propionibacterium
Pseudoramibacter
Roseburia
Ruminiclostridium
Ruminococcus
Sporobacter
Stomatobaculum
Streptococcus
Sutterella
Turicibacter
Tyzzerella
Veillonella
Genus
Alcanivorax
Alteromonas
Arcobacter
Bacillus
Brevibacillus
Brumimicrobium
Burkholderia
Citromicrobium
Colwellia
Delftia
Desulfococcus
Erythrobacter
Flavobacterium
Fluviicola
Formosa
Geobacter
Glaciecola
Halomonas
Hyphomicrobium
Lewinella
Loktanella
Maribacter
Marinimicrobium
Marinobacter
Marinomonas
Moritella
Mycobacterium
Nitrospina
Novosphingobium
Oceanicaulis
Octadecabacter
Oleispira
Olleya
Paracoccus
Parvibaculum
Phaeobacter
Photobacterium
Planctomyces
Polaribacter
Prochlorococcus
Pseudoalteromonas
Pseudomonas
Pseudoruegeria
Psychroflexus
Psychromonas
Psychroserpens
Robiginitalea
Saccharospirillum
Sediminicola
Shewanella
Sphingomonas
Spirulina
Staphylococcus
Sulfurimonas
Synechococcus
Tenacibaculum
Tepidibacter
Thalassobacter
Thalassomonas
Thalassospira
Thiobacillus
Ulvibacter
Verrucomicrobium
Vibrio
Winogradskyella
Yonghaparkia
Genus
Acinetobacter
Actinomyces
Actinoplanes
Adhaeribacter
Aeromicrobium
Afifella
Agrobacterium
Alcaligenes
Alicyclobacillus
Arthrobacter
Bacillus
Bdellovibrio
Bosea
Bradyrhizobium
Burkholderia
Caulobacter
Cellulomonas
Cellvibrio
Chitinophaga
Chryseobacterium
Clostridium
Cryocola
Desulfococcus
Desulfomicrobium
Desulfosporosinus
Devosia
Edaphobacter
Flavisolibacter
Flavobacterium
Gallionella
Gemmata
Geobacter
Geodermatophilus
Geothrix
Halothiobacillus
Herbiconiux
Hyphomicrobium
Janthinobacterium
Kribbella
Limnohabitans
Lysinibacillus
Mesorhizobium
Microbacterium
Modestobacter
Mycobacterium
Mycoplana
Nitrospira
Nocardioides
Novosphingobium
Ochrobactrum
Paenibacillus
Pedobacter
Pedomicrobium
Pelotomaculum
Phenylobacterium
Pilimelia
Pirellula
Planctomyces
Pseudomonas
Pseudonocardia
Ramlibacter
Rhizobium
Rhodococcus
Rhodoferax
Rhodoplanes
Robiginitalea
Salinibacterium
Shewanella
Sphingobium
Sphingomonas
Sphingopyxis
Sporosarcina
Streptomyces
Syntrophus
Treponema
Variovorax
