# Supplementary material for: Benchmarking taxonomic assignments based on 16S rRNA gene profiling of the microbiota from commonly sampled environments
Source: Gigascience. 2018 May 11;7(5):giy054. doi: 10.1093/gigascience/giy054 (PMC5967554; doi:10.1093/gigascience/giy054)
Supplement: Supplemental material [file giy054_supp.zip › Figure_S3.pdf]

Tool \ Database	SILVA 128	Greengenes 13_8	RDP v16	NCBI (mapref 2.2)
MAPseq v1.2.2	x	x		x
mothur v1.39.5	x		x	
QIIME v1.9.1	x	x		
QIIME 2 v2017.11	x	x		
