# Supplementary figures and images for: Benchmarking taxonomic assignments based on 16S rRNA gene profiling of the microbiota from commonly sampled environments
Source: Gigascience. 2018 May 11;7(5):giy054. doi: 10.1093/gigascience/giy054 (PMC5967554; doi:10.1093/gigascience/giy054)

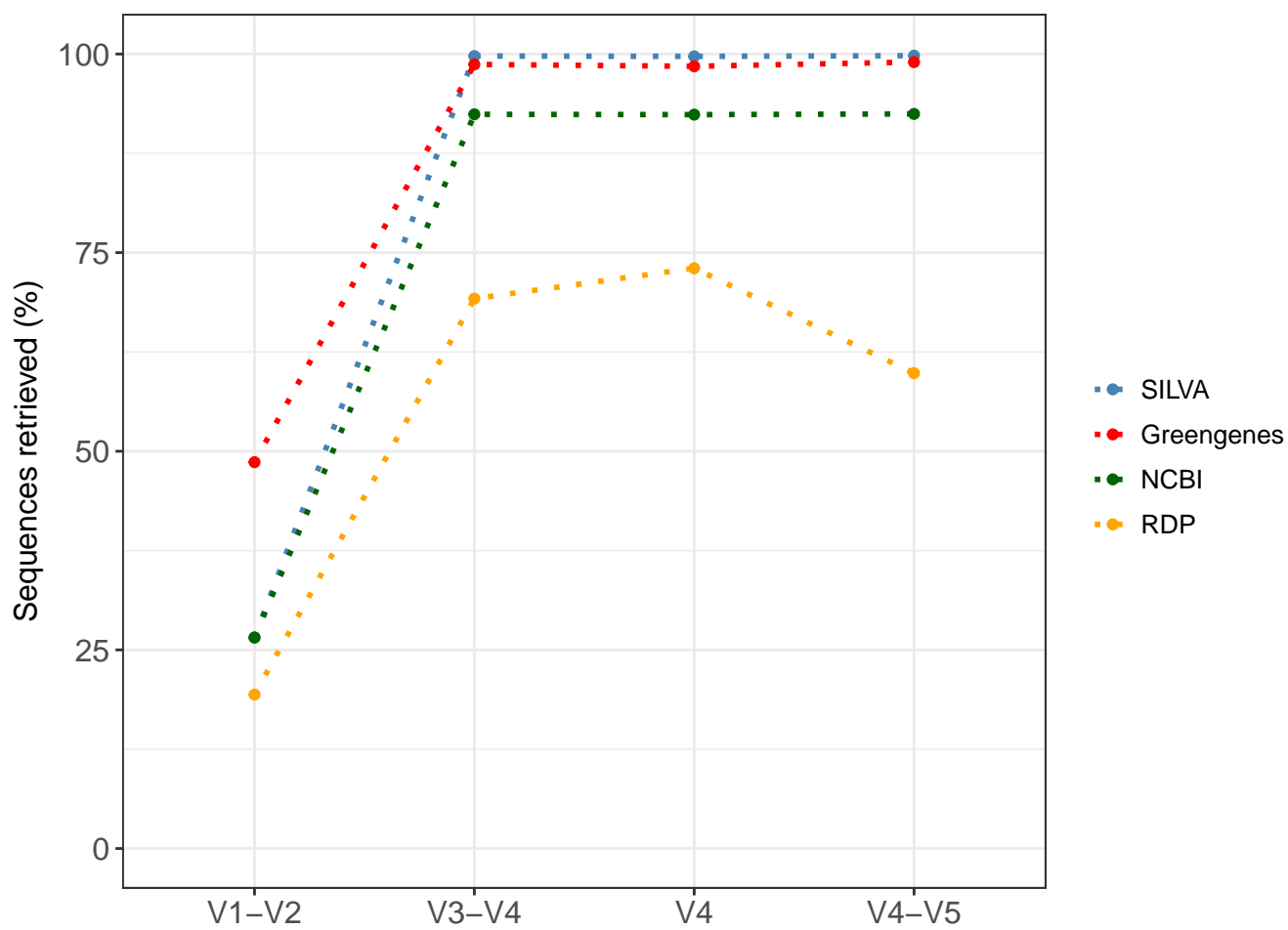

Supplement: Supplemental material [file giy054_supp.zip › Figure_S2.pdf]

Human gut

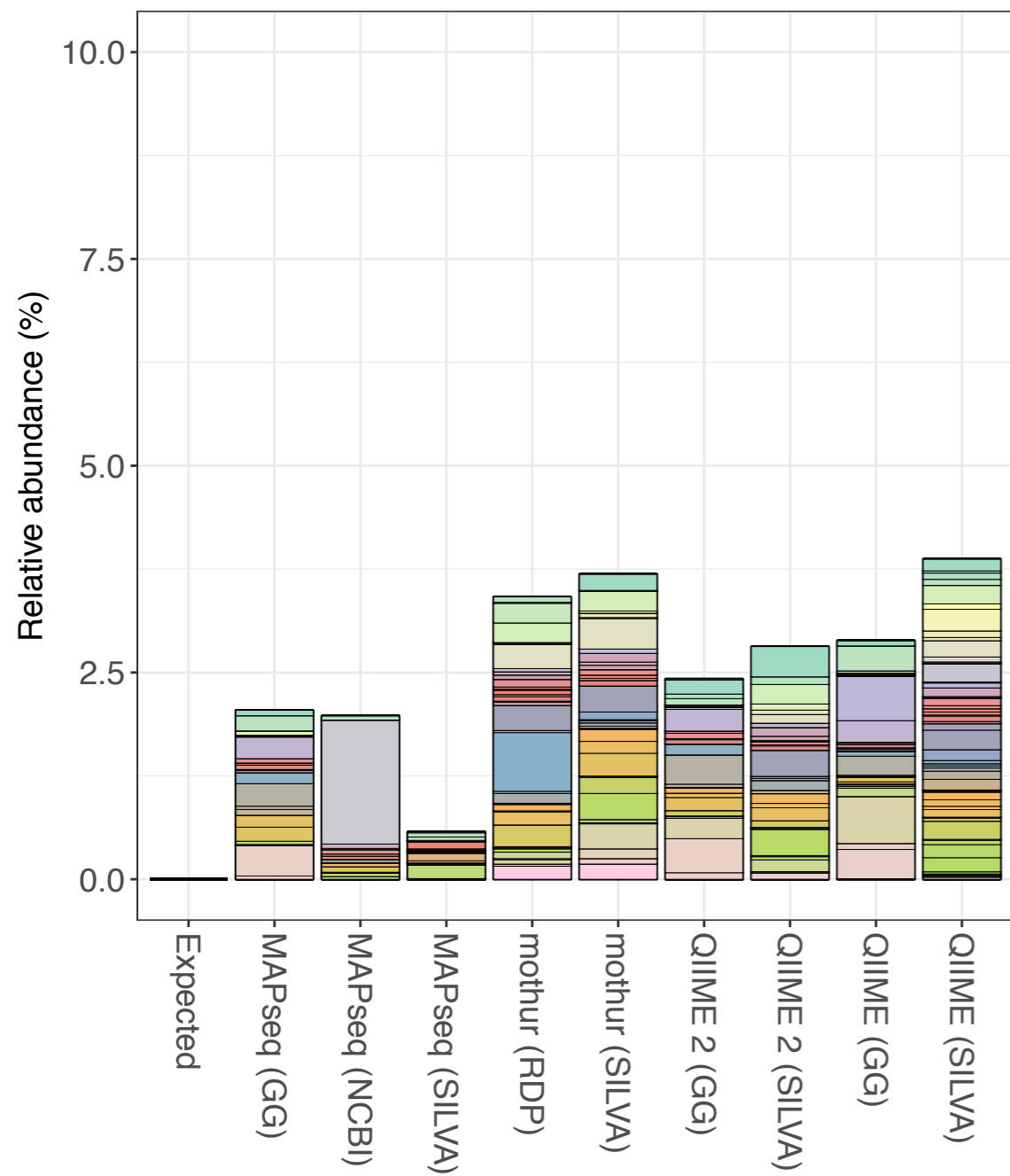

Ocean

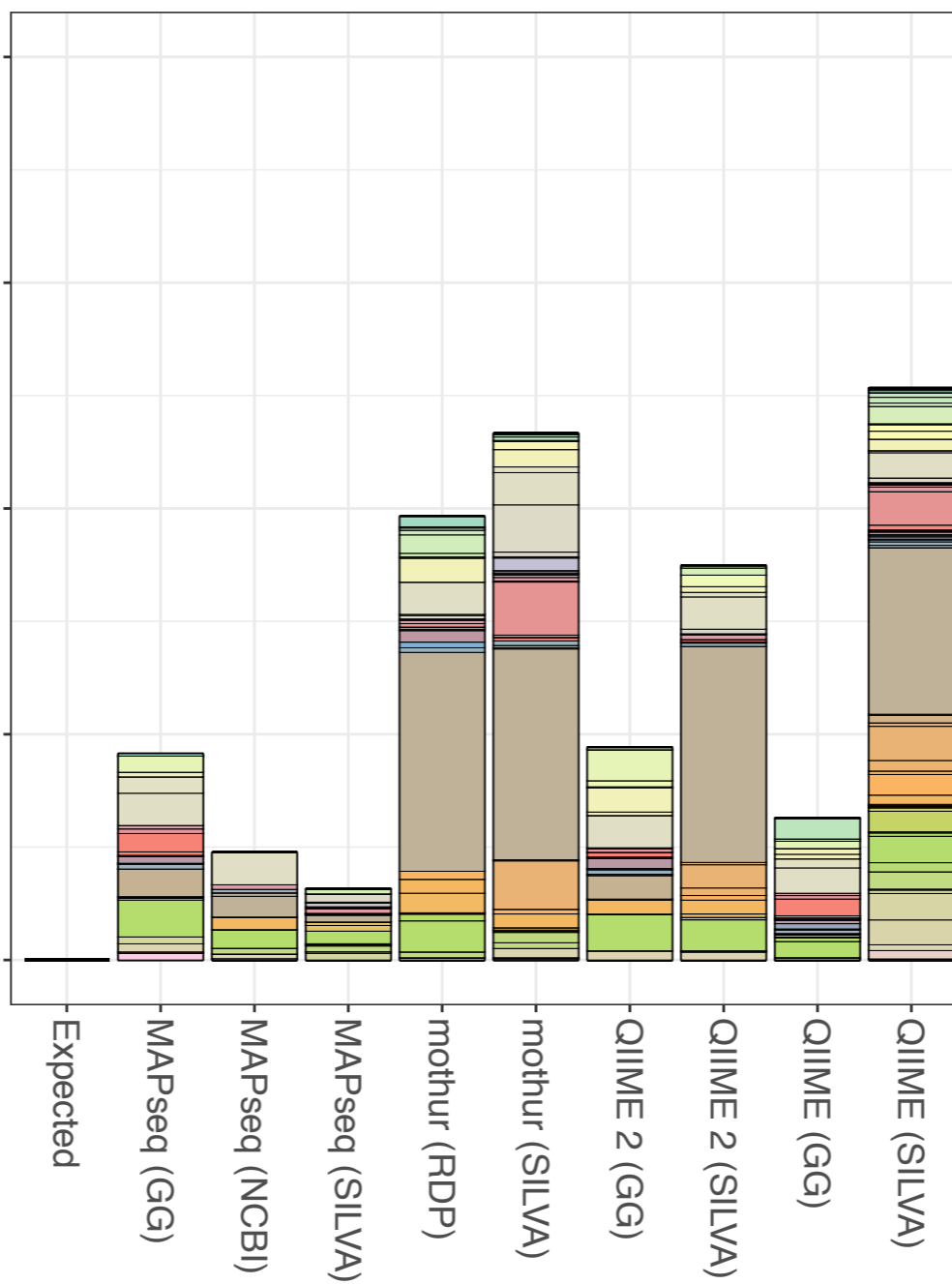

Soil

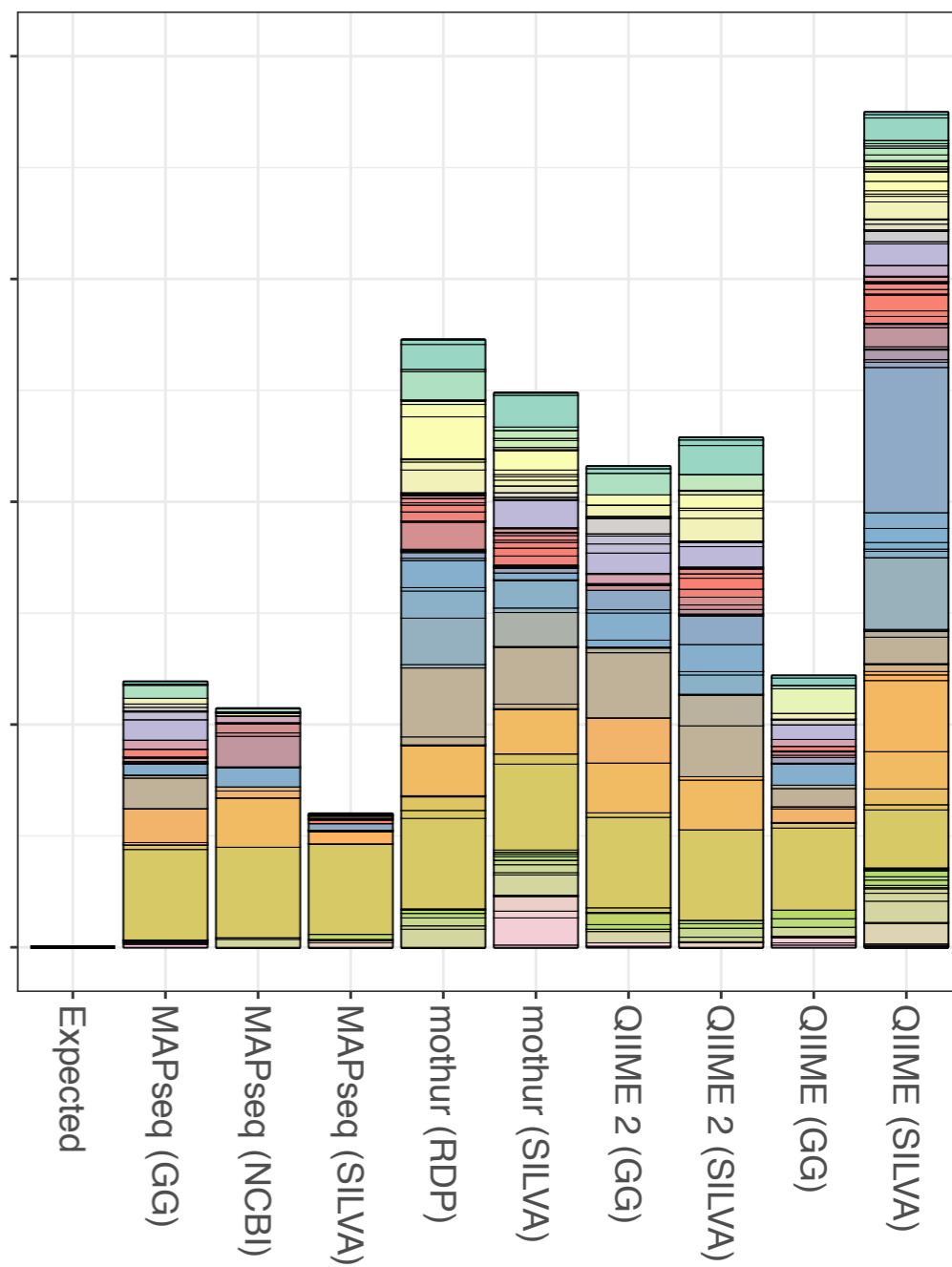

Supplement: Supplemental material [file giy054_supp.zip › Figure_S4.pdf]

Recall Precision F-score

Human gut

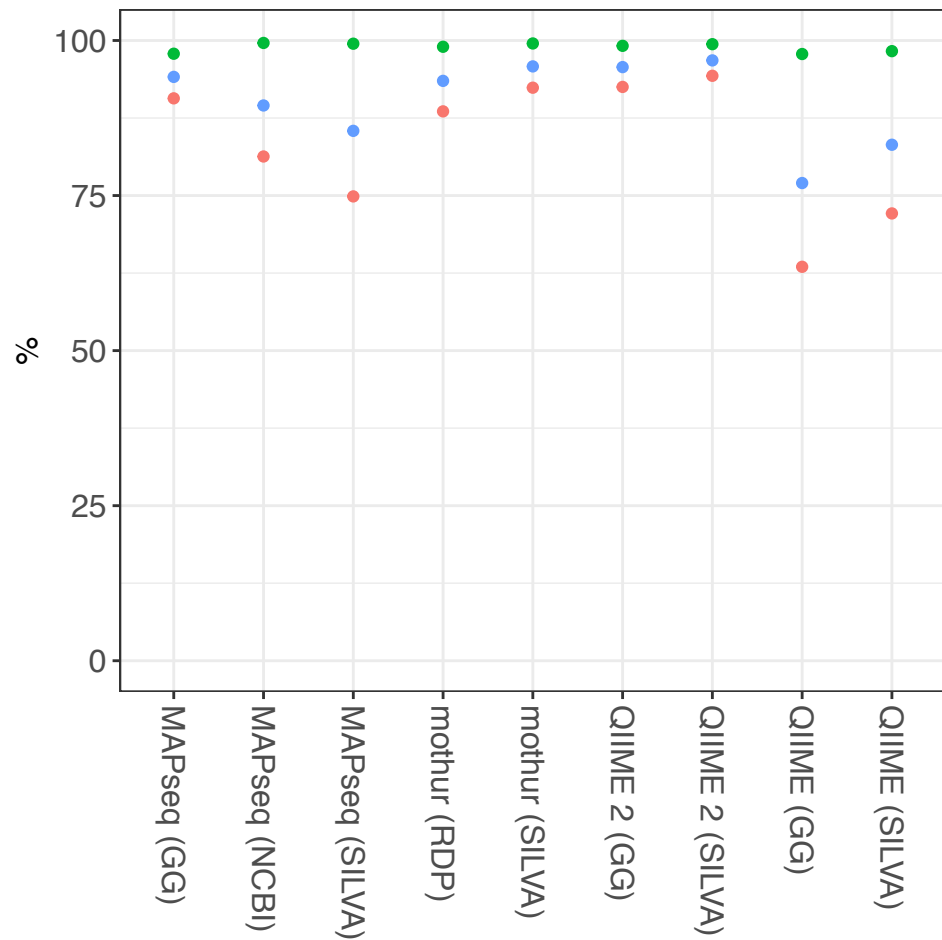

Ocean

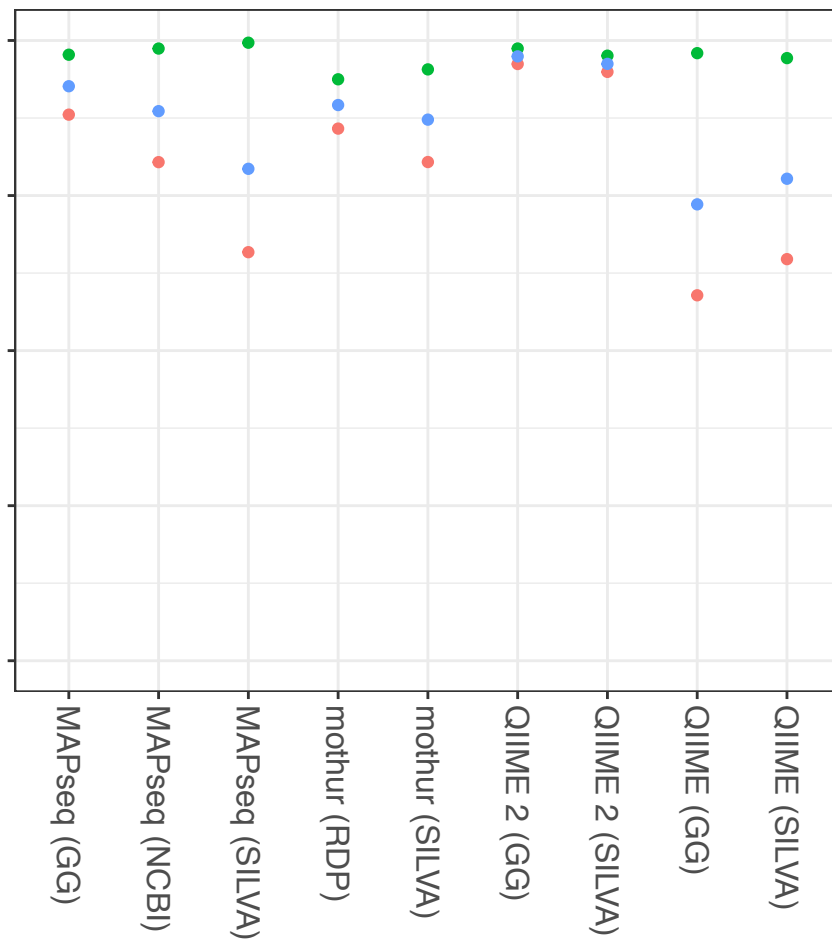

Soil

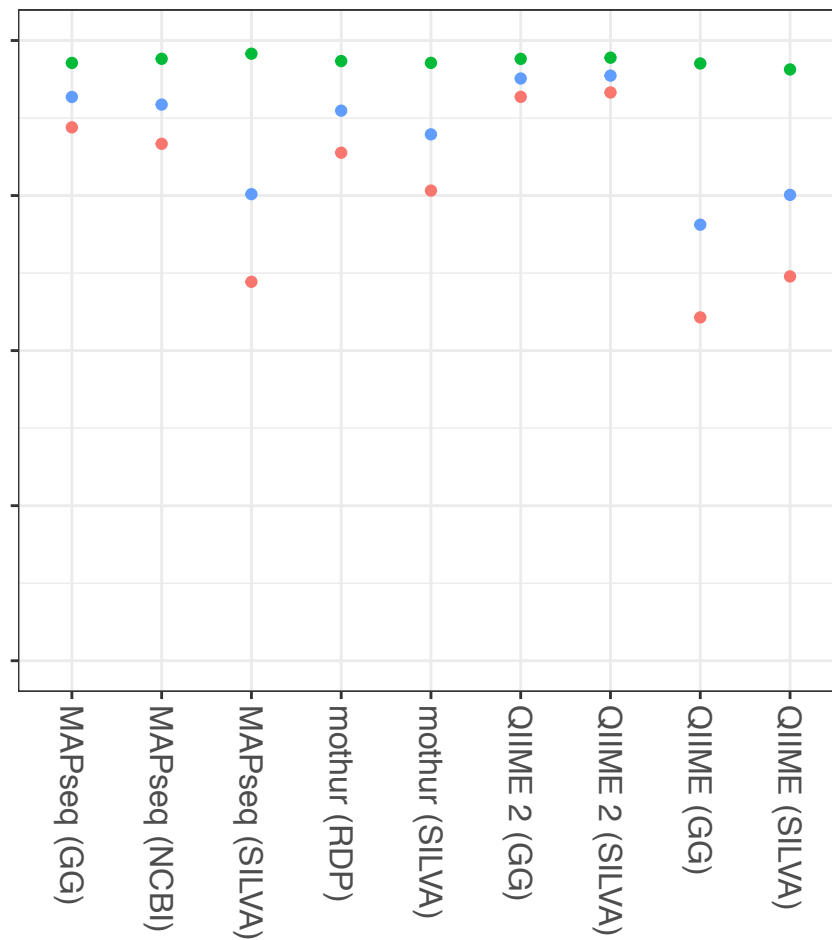

Supplement: Supplemental material [file giy054_supp.zip › Figure_S5.pdf]
