# Supplementary material for: Benchmarking taxonomic assignments based on 16S rRNA gene profiling of the microbiota from commonly sampled environments
Source: Gigascience. 2018 May 11;7(5):giy054. doi: 10.1093/gigascience/giy054 (PMC5967554; doi:10.1093/gigascience/giy054)
Supplement: Response_to_Reviewer_Comments_Original_Submission.pdf [file giy054_response_to_reviewer_comments_original_submission.pdf]

Author's Response To Reviewer Comments

Close

Reviewer #1
========

Independent benchmarks like this are important for guiding methods choices for researchers. I enjoyed reading this study and feel that it will be valuable to readers. I have a few questions and suggestions below.

R: We appreciate the positive feedback from the reviewer and have addressed all his comments and suggestions below and in the revised manuscript.

ln 20 - QIIME, mothur, and QIIME2 all utilize multiple different taxonomic classifiers. So multiple choices exist within each platform, there is no standard "mothur" or "QIIME" method (their defaults are essentially RDP classifier and a uclust-based classifier). It would be helpful to clarify this information in the text if not the abstract, e.g., the mothur classifier should be called RDP.

R: Based on both reviewers' comments, we have clarified this in the "Abstract" (lines 23-26), "Background" (lines 100-102) and "Discussion" sections (lines 262-264).

ln 29 - QIIME2 also appears to have higher F-measure scores, perhaps this should be mentioned here.

R: We have now included this information in the "Abstract" (lines 29-31).

ln 77-79 - what about the QIIME2 pre-print cited below? it does not cover Mapseq but is a benchmark of a number of different commonly used classifiers and marker-gene regions (albeit not an independent comparison).

R: We have now referenced this paper in the revised manuscript (lines 79-80).

ln 91-93 - what about the strengths of mock communities/weaknesses of simulation? this section seems to imply that mock communities are necessarily inferior, and simulations are not prone to their own limitations.

R: We agree with the reviewer and have now highlighted the importance of using both mock communities and in silico approaches in the "Background" section (lines 91-98).

ln 121 - variation is realistic, but not entirely random variation. Why not mutate simulated sequences after extracting the variable regions? It seems that much of the variation may otherwise fall outside of the variable regions and not impact this simulation.

R: We agree with the reviewer and have re-generated our simulated reads by mutating the sequences after extracting the variable regions instead. The new results are depicted in the revised version of the manuscript but show no significant differences to the original results presented. We have also replaced the original FASTQ files with this new set in the GigaDB FTP site.

ln 141-143 - why not at least show species-level results in the supplement if not main text? It is

important to demonstrate why researchers should be cautious about species-level classifications.

R: We decided not to present the species-level assignment due to two main reasons: i) it has already been previously described (Golob, et al. 2017, PMID: 28558684) that 16S rRNA gene classification with amplicon-based sequences is severely limited at the species level, with only ~ 12% of correctly assigned sequences, and miscall rates of ~ 20%; ii) there is significant inconsistency in species nomenclature between the databases we tested (e.g. many species are just labelled "[Genus] sp.", whereas the RDP database does not even output species assignments), which would make an assessment of recall/precision challenging and possibly misleading, especially given the low number of assigned sequences. We have now made this clearer in the revised manuscript as well (lines 149-155).

ln 242 - parameter selection will greatly impact precision/recall scores, and e.g. increasing confidence thresholds for QIIME2 or mothur classifiers will improve precision at the expense of recall. Mapseq may have similar performance tradeoffs — but overall I wonder if altering confidence thresholds for these other methods can approach the miscall rate of mapseq. At the very least, this should be mentioned in the discussion. The QIIME2 classifier pre-print cited by this work covers parameter permutations that maximize recall/precision (the default maximizes F-measure).

R: In this paper the aim was to assess the recommended and most widely-used parameters for each tool, as these will be what most users will likely be using in their analyses. We agree that tweaking individual settings might provide improvements to recall/precision estimates for each pipeline, but this was beyond the scope of this work and would have increased the number of comparisons performed exponentially. Therefore, as per the reviewer's suggestion we have now discussed this topic in the "Discussion" section of the revised manuscript (lines 262-264).

Reviewer #2
========

Overview

In this paper the authors have sought to evaluate the performance of the 4 main packages and their default classifiers/settings used in the taxonomic profiling of rRNA sequences. They did this using synthetic simulated read sets representative of 3 commonly studied microbiome environments and investigated the role of locus and reference database selection on classification metrics.

This is well done research that will form a useful benchmark for researchers engaged in rRNA taxonomic profiling to help design and conduct their own studies.

R: We thank the reviewer's positive remarks and have addressed all his comments below and in the revised manuscript.

General Comments

It should be emphasised throughout the manuscript that as of January 1st 2018, QIIME1 is deprecated and no longer supported by the developers (https://qiime.wordpress.com/2018/01/03/qiime-2-has-succeeded-qiime-1/). Therefore, QIIME1 is no longer recommended to be used at all.

R: We have now mentioned this in the "Background" (lines 69-71) and "Discussion" sections (lines

246-248).

Secondly, it is probably worth emhpasising that QIIME1, QIIME2 and mothur are very large toolsets with many parts and functions capable of more than just taxonomic assignment. Even for taxonomic assignment specifically, it could do with being clarified that mothur (RDP port, k-nearest neighbours, wang k-mer method) and QIIME (UCLUST, RDP, rtax, sortmerna, mothur's methods etc) implement a variety of optional alternative taxonomic classifiers. Comparing the performance of the default classifiers with default settings is very useful as that is what most users will end up using but it should be made clear in the manuscript that this work doesn't investigate these package options beyond database selection.

R: We have now included this information in the “Background” (lines 60-64) and “Discussion” sections (lines 262-264).

Minor Comments

Line 59: Possibly should be emhpasised that mothur, QIIME, and QIIME2 are large packages with lots of functions and uses beyond taxonomic assignment.

R: We have now added this information to the revised manuscript (lines 60-62).

Line 68: Although the RDP classifier can also be used optionally within QIIME fairly easily (although as the authors have stated is not default).

R: We have now mentioned the possibility of using different classifiers in the “Discussion” section (lines 262-264).

Line 69: Mothur doesn't wrap RDP but totally reimplements RDP in C++ (http://blog.mothur.org/2016/01/12/mothur-and-qiime/)

R: We have now clarified this as per the reviewer’s suggestion (lines 67-69).

Line 70: Worth highlighting that QIIME2 is intended to totally replace QIIME.

R: As mentioned above, we have now included this information in both the “Background” (lines 69-71) and “Discussion” sections (lines 246-248).

Line 124: Please add a citation for these primers if possible.

R: References for each primer set have now been added to the text (lines 129 and 329) and the accompanying supplementary table (Table S1).

Line 125: Can you clarify why RDP and MAPseq NCBI databases weren't used in this primer analysis?

R: We initially decided to focus on SILVA and Greengenes since they are most frequently used databases. However, we have now included the results for RDP and NCBI as well in the revised manuscript (lines 129-139 and Fig. S2).

Line 143: Has anyone done an analysis supporting the too limited resolution of this locus for species level classification?

R: In another benchmarking paper (Golob, et al. 2017, PMID: 28558684) it was shown that QIIME and mothur can only assign ~ 12% of 16S rRNA amplicon sequences to the correct species, while additionally presenting a miscall rate of ~ 21%. We have now cited this reference in the revised manuscript (line 150).

Line 151: Can you add the microbiome environment specific performance metrics for each tool as a (possibly supplemental) table instead of just the averaged metrics as report in Table 1? Acknowledging this involves some degree of overlap/redundancy to Figure 2.

R: We have now provided this information in three new supplementary tables (Tables S2, S3 and S4).

Line 208: As with the previous comment, despite the more detailed heatmap breakdown in Figure 4. It would be nice to see the overall dissimilarity metrics presented unaggregated by method and biome in a supplemental table.

R: This information has now also been added to the above-mentioned tables (Tables S2, S3 and S4).

Line 238: It might be good to further emphasis that is support the developer's decision to no longer support QIIME v1, especially with the tendency of outdated bioinformatics to linger and be widely used!

R: As stated above, we have now mentioned this in the "Background" (lines 69-71) and "Discussion" sections (lines 246-248).

Line 246: Do you believe this is likely to be due to overhead from QIIME2's zipping and unzipping of input files?

R: From our experience, QIIME 2's computational demand appears to be more significantly affected by the size of the database. It is possible that this is influenced by the uncompressing and compressing of the QZA files (the proprietary format used by QIIME 2), but we prefer not to speculate on this matter.

Line 251: Could add emphasis that these unevaluated alternatives includes other classifiers and settings within the software packages that were tested in this paper.

R: We have now mentioned this in the "Discussion" (lines 262-264).

Line 312: Using this script's default maximum primer mismatch of 3?
Line 315: What platform error profile was used when simulating reads with ART? MSv3?

R: Yes, we used the default primer mismatch of 3 and the MiSeq v3 error profile with ART. We have added this information to the "Methods" section (lines 326-335).

Line 337: Why was 99% clustered SILVA used for QIIME2 but 97% for QIIME1?

R: QIIME by default makes use of the Greengenes database clustered at 97%. To make a fair comparison across QIIME, we decided to cluster the SILVA database at the same level. On the other hand, the tutorials and standard operating procedures (SOP) of QIIME 2 advise and provide pre-trained databases of Greengenes and SILVA only at 99%. We hypothesize that these differences in the preferred clustering threshold might be related to the distinct assignment pipelines and default

methods between the tools (UCLUST in QIIME vs. the Naïve Bayes classifier in QIIME 2).

Line 361: Presumably on a system under no other load? Was this run once or rerun a few times to determine variance of memory/cpu usage?

R: To assess the computational cost we calculated the average CPU time and memory usage across three different data points (one for each biome) after running each analysis in our cluster here at the EBI (which allocates the resources required for each job). We have now added error bars with the standard deviation to Fig. 3, showing the high consistency of these measurements.

References: Inconsistent capitalisation of titles, inclusion of editors and publisher information (mainly Nature Publishing Group) but others from the same publisher don't e.g. ref 4.

R: We have now corrected these formatting issues.

Figure 3 Legend: Is the SILVA database referenced here at different 97-99% clustering levels mentioned?

R: In the original manuscript we used the 97% clustered SILVA database for QIIME and the 99% one for QIIME 2. We realized that for assessing the computational cost this might be misleading, so we have now modified the analyses to use the same SILVA database across all comparisons (at a 99% clustering threshold). We have now also clarified this in the text (lines 380-382).

Figure S3: Explain and/or cite not using greengenes due to the alignment issue? It does seem not recommended. The methods section may benefit from inclusion of this database information.

R: We have now included this information in the revised manuscript (lines 355-356) and in the Fig. S3 legend (lines 457-458), with a citation to the mothur SOP.

Figure S4: Would be nice to include a key as per Figure 1 instead of needing to cross-reference to the tables.

R: Although we agree with the reviewer, given that the miscalled taxa correspond to over 100 different genera, it would be very challenging to have a figure key with discernible colours for each genus (especially given how small some of the stacked bars are). We realize it is not an ideal solution, but we have decided to leave that information as separate supplementary tables (now Tables S5, S6 and S7).

Close
