# Supplementary material for: Benchmarking taxonomic assignments based on 16S rRNA gene profiling of the microbiota from commonly sampled environments
Source: Gigascience. 2018 May 11;7(5):giy054. doi: 10.1093/gigascience/giy054 (PMC5967554; doi:10.1093/gigascience/giy054)
Supplement: Reviewer_1_Report_(Original_Submission) -- Nicholas Bokulich — 2/20/2018 Reviewed [file giy054_reviewer_1_report_(original_submission).pdf]

Reviewer Report

Title: Benchmarking taxonomic assignments based on 16S rRNA gene profiling of the microbiota from commonly sampled environments

Version: Original Submission **Date:** 2/20/2018

Reviewer name: Nicholas Bokulich

Reviewer Comments to Author:

Independent benchmarks like this are important for guiding methods choices for researchers. I enjoyed reading this study and feel that it will be valuable to readers. I have a few questions and suggestions below.ln 20 - QIIME, mothur, and QIIME2 all utilize multiple different taxonomic classifiers. So multiple choices exist within each platform, there is no standard "mothur" or "QIIME" method (their defaults are essentially RDP classifier and a uclust-based classifier). It would be helpful to clarify this information in the text if not the abstract, e.g., the mothur classifier should be called RDP.ln 29 - QIIME2 also appears to have higher F-measure scores, perhaps this should be mentioned here.ln 77-79 - what about the QIIME2 pre-print cited below? it does not cover Mapseq but is a benchmark of a number of different commonly used classifiers and marker-gene regions (albeit not an independent comparison).ln 91-93 - what about the strengths of mock communities/weaknesses of simulation? this section seems to imply that mock communities are necessarily inferior, and simulations are not prone to their own limitations.ln 121 - variation is realistic, but not entirely random variation. Why not mutate simulated sequences after extracting the variable regions? It seems that much of the variation may otherwise fall outside of the variable regions and not impact this simulation.ln 141-143 - why not at least show species-level results in the supplement if not main text? It is important to demonstrate why researchers should be cautious about species-level classifications.ln 242 - parameter selection will greatly impact precision/recall scores, and e.g. increasing confidence thresholds for QIIME2 or mothur classifiers will improve precision at the expense of recall. Mapseq may have similar performance tradeoffs — but overall I wonder if altering confidence thresholds for these other methods can approach the miscall rate of mapseq. At the very least, this should be mentioned in the discussion. The QIIME2 classifier pre-print cited by this work covers parameter permutations that maximize recall/precision (the default maximizes F-measure).

Level of Interest

Please indicate how interesting you found the manuscript: An article of importance in its field

Quality of Written English

Please indicate the quality of language in the manuscript: Acceptable

Declaration of Competing Interests

Please complete a declaration of competing interests, considering the following questions:

- Have you in the past five years received reimbursements, fees, funding, or salary from an organisation that may in any way gain or lose financially from the publication of this manuscript, either now or in the future?

- Do you hold any stocks or shares in an organisation that may in any way gain or lose financially from the publication of this manuscript, either now or in the future?
- Do you hold or are you currently applying for any patents relating to the content of the manuscript?
- Have you received reimbursements, fees, funding, or salary from an organization that holds or has applied for patents relating to the content of the manuscript?
- Do you have any other financial competing interests?
- Do you have any non-financial competing interests in relation to this paper?

If you can answer no to all of the above, write 'I declare that I have no competing interests' below. If your reply is yes to any, please give details below.

I declare that I have no competing interests

I agree to the open peer review policy of the journal. I understand that my name will be included on my report to the authors and, if the manuscript is accepted for publication, my named report including any attachments I upload will be posted on the website along with the authors' responses. I agree for my report to be made available under an Open Access Creative Commons CC-BY license (http://creativecommons.org/licenses/by/4.0/). I understand that any comments which I do not wish to be included in my named report can be included as confidential comments to the editors, which will not be published.

I agree to the open peer review policy of the journal

To further support our reviewers, we have joined with Publons, where you can gain additional credit to further highlight your hard work (see: https://publons.com/journal/530/gigascience). On publication of this paper, your review will be automatically added to Publons, you can then choose whether or not to claim your Publons credit. I understand this statement.

Yes
