# Supplementary material for: Benchmarking taxonomic assignments based on 16S rRNA gene profiling of the microbiota from commonly sampled environments
Source: Gigascience. 2018 May 11;7(5):giy054. doi: 10.1093/gigascience/giy054 (PMC5967554; doi:10.1093/gigascience/giy054)
Supplement: Reviewer_2_Report_(Original_Submission) -- Finlay Maguire — 3/19/2018 Reviewed [file giy054_reviewer_2_report_(original_submission).pdf]

Reviewer Report

Title: **Benchmarking taxonomic assignments based on 16S rRNA gene profiling of the microbiota from commonly sampled environments**

Version: **Original Submission** **Date:** 3/19/2018

Reviewer name: **Finlay Maguire**

Reviewer Comments to Author:

OverviewIn this paper the authors have sought to evaluate the performance of the 4 main packages and their default classifiers/settings used in the taxonomic profiling of rRNA sequences.They did this using synthetic simulated read sets representative of 3 commonlystudied microbiome environments and investigated the role of locus andreference database selection on classification metrics.This is well done research that will form a useful benchmark for researchers engaged in rRNA taxonomic profiling to help design and conduct their own studies.## General CommentsIt should be emphasised throughout the manuscript that as of January 1st 2018, QIIME1 is deprecated and no longer supported by the developers(https://qiime.wordpress.com/2018/01/03/qiime-2-has-succeeded-qiime-1/).Therefore, QIIME1 is no longer recommended to be used at all.Secondly, it is probably worth emhpasising that QIIME1, QIIME2 and mothurare very large toolsets with many parts and functions capable of more than just taxonomic assignment. Even for taxonomic assignment specifically, it could do with being clarified that mothur (RDP port, k-nearest neighbours, wang k-mer method) and QIIME (UCLUST, RDP, rtax, sortmerna, mothur's methods etc) implement a variety of optionalalternative taxonomic classifiers.Comparing the performance of the default classifiers with default settings is very useful as that is what most users will end up using but it should be made clear in the manuscript that this work doesn't investigate these package options beyond database selection.## Minor CommentsLine 59: Possibly should be emhpasised that mothur, QIIME, and QIIME2 are large packages with lots of functions and uses beyond taxonomic assignment.Line 68: Although the RDP classifier can also be used optionally within QIIME fairly easily (although as the authors have stated is not default).Line 69: Mothur doesn't wrap RDP but totally reimplements RDP in C++ (http://blog.mothur.org/2016/01/12/mothur-and-qiime/)Line 70: Worth highlighting that QIIME2 is intended to totally replaceQIIME.Line 124: Please add a citation for these primers if possible.Line 125: Can you clarify why RDP and MAPseq NCBI databases weren't used in this primer analysis?Line 143: Has anyone done an analysis supporting the too limited resolution ofthis locus for species level classification?Line 151: Can you add the microbiome environment specific performancemetrics for each tool as a (possibly supplemental) table instead of just the averaged metrics as report in Table 1? Acknowledging this involves some degree ofoverlap/redundancy to Figure 2.Line 208: As with the previous comment, despite the more detailed heatmapbreakdown in Figure 4. It would be nice to see the overall dissimilaritymetrics presented unaggregated by method and biome in a supplemental table. Line 238: It might be good to further emphasis that is support the developer's decision to no longer support QIIME v1,especially with the tendency of outdated bioinformatics to linger and be widely used!Line 246: Do you believe this is likely to be due to overhead from QIIME2's zipping and unzippingof input files? Line 251: Could add emphasis that these unevaluated alternatives includes other classifiers and settings within the software packages that were tested in this paper.Line 312: Using this script's default maximum primer mismatch of 3?Line 315: What platform error profile was used when simulating reads with ART? MSv3?Line 337: Why was 99% clustered SILVA used for QIIME2 but 97% for QIIME1? Line 361: Presumably on a system under no other load? Was this run onceor rerun a few times to determine variance of memory/cpu usage?References: Inconsistent capitalisation of titles, inclusion of editors andpublisher information (mainly Nature Publishing Group)but others from the same publisher don't e.g. ref 4.Figure 3 Legend: Is the SILVA database referenced here at different 97-99% clustering levels mentioned?Figure S3: Explain and/or cite not using greengenes due to the alignment issue? It does seem not recommended. The methods section may benefit from inclusionof this database information.Figure S4: Would be nice to include a key as per Figure 1 instead of needing to cross-reference to the tables.

Level of Interest

Please indicate how interesting you found the manuscript: An article of importance in its field

Quality of Written English

Please indicate the quality of language in the manuscript: Acceptable

Declaration of Competing Interests

Please complete a declaration of competing interests, considering the following questions:

- Have you in the past five years received reimbursements, fees, funding, or salary from an organisation that may in any way gain or lose financially from the publication of this manuscript, either now or in the future?
- Do you hold any stocks or shares in an organisation that may in any way gain or lose financially from the publication of this manuscript, either now or in the future?
- Do you hold or are you currently applying for any patents relating to the content of the manuscript?
- Have you received reimbursements, fees, funding, or salary from an organization that holds or has applied for patents relating to the content of the manuscript?
- Do you have any other financial competing interests?
- Do you have any non-financial competing interests in relation to this paper?

If you can answer no to all of the above, write 'I declare that I have no competing interests' below. If your reply is yes to any, please give details below.

I declare that I have no competing interests

I agree to the open peer review policy of the journal. I understand that my name will be included on my report to the authors and, if the manuscript is accepted for publication, my named report including any attachments I upload will be posted on the website along with the authors' responses. I agree for my report to be made available under an Open Access Creative Commons CC-BY license (http://creativecommons.org/licenses/by/4.0/). I understand that any comments which I do not wish to be included in my named report can be included as confidential comments to the editors, which will not be published.

I agree to the open peer review policy of the journal

To further support our reviewers, we have joined with Publons, where you can gain additional credit to further highlight your hard work (see: https://publons.com/journal/530/gigascience). On publication of this paper, your review will be automatically added to Publons, you can then choose whether or not to claim your Publons credit. I understand this statement.

Yes
